# Predictive network modeling of the high-resolution dynamic plant transcriptome in response to nitrate

**DOI:** 10.1186/gb-2010-11-12-r123

**Published:** 2010-12-23

**Authors:** Gabriel Krouk, Piotr Mirowski, Yann LeCun, Dennis E Shasha, Gloria M Coruzzi

**Affiliations:** 1 Center for Genomics and Systems Biology, Department of Biology, New York University, 100 Washington Square East, 1009 Main Building, New York, NY 10003, USA; 2 Biochimie et Physiologie Moléculaire des Plantes, UMR 5004 CNRS/INRA/SupAgro-M/UM2, Institut de Biologie Intégrative des Plantes, Place Viala, 34060 Montpellier Cedex, France; 3 Courant Institute of Mathematical Sciences, New York University, New York, NY 10003, USA

## Abstract

**Background:**

Nitrate, acting as both a nitrogen source and a signaling molecule, controls many aspects of plant development. However, gene networks involved in plant adaptation to fluctuating nitrate environments have not yet been identified.

**Results:**

Here we use time-series transcriptome data to decipher gene relationships and consequently to build core regulatory networks involved in *Arabidopsis *root adaptation to nitrate provision. The experimental approach has been to monitor genome-wide responses to nitrate at 3, 6, 9, 12, 15 and 20 minutes using Affymetrix ATH1 gene chips. This high-resolution time course analysis demonstrated that the previously known primary nitrate response is actually preceded by a very fast gene expression modulation, involving genes and functions needed to prepare plants to use or reduce nitrate. A state-space model inferred from this microarray time-series data successfully predicts gene behavior in unlearnt conditions.

**Conclusions:**

The experiments and methods allow us to propose a temporal working model for nitrate-driven gene networks. This network model is tested both *in silico *and experimentally. For example, the over-expression of a predicted gene hub encoding a transcription factor induced early in the cascade indeed leads to the modification of the kinetic nitrate response of sentinel genes such as *NIR*, *NIA2*, and *NRT1.1*, and several other transcription factors. The potential nitrate/hormone connections implicated by this time-series data are also evaluated.

## Background

Higher plants, which constitute a main entry of nitrogen in to the food chain, acquire nitrogen mainly as nitrate (NO_3_^-^). Soil concentrations of this mineral ion can fluctuate dramatically in the rhizosphere, often resulting in limited growth and yield [1]. Thus, understanding plant adaptation to fluctuating nitrogen levels in the soil is a challenging task with potential consequences for health, the environment, and economies [2-4].

The first genomic studies on NO_3_^- ^responses in plants were published 10 years ago [5]. To date, data monitoring gene expression in response to NO_3_^- ^provision from more than 100 Affymetrix ATH1 chips have been published [5-12]. Meta-analysis of microarray data sets from several different labs demonstrated that at least a tenth of the genome can potentially be regulated by nitrogen provision, depending on the context [2,9,13,14]. Despite these extensive efforts of characterization, only a limited number of molecular actors that alter NO_3_^-^-induced gene regulation have been identified so far. The first molecular actor identified is NRT1.1, a dual affinity NO_3_^- ^transporter that has recently been proposed to also participate in a NO_3_^-^-sensing system by several studies from different laboratories. A mutation in the *NRT1.1 *gene has been shown to alter plant responses to NO_3_^- ^provision by changing lateral root development in NO_3_^-^-rich patches of soil [15,16] and to affect control of gene expression [17-20]. Additionally, mutations in the genes *CIPK8 *and *CIPK23*, encoding kinases, the NIN-like protein gene *NLP7*, and the *LBD37*/*38*/*39 *genes have been shown to alter induction of downstream genes by NO_3_^- ^[20-23]. Other regulatory proteins have been shown to control plant development in response to NO_3_^- ^provision (such as ANR1 for lateral root development), but no evidence has so far demonstrated their role in the control of gene expression in response to NO_3_^- ^provision [24]. Importantly, the downstream networks of genes affected by such regulatory proteins have not been identified.

In this study, our aim is to provide a systems-wide view of NO_3_^- ^signal propagation through dynamic regulatory gene networks. To do so, we generated a high-resolution dynamic NO_3_^- ^transcriptome from plants treated with nitrate from 0 to 20 minutes, and modeled the resulting sequence using a dynamical model. Instead of learning the dynamics directly from the gene expression sequence, we took into account uncertainty and acquisition errors, and used a state-space model (SSM). The latter defined the observed gene expression time series (denoted as **y**(*t*)) as being generated by a hidden 'true' sequence of gene expressions **z**(*t*). This approach enabled us to both incorporate uncertainty about the measured mRNA and model the gene regulation network by simple linear dynamics on the hidden variables **x**(*t*) (so-called 'states'), thus reducing the number of (unknown) free parameters and the associated risk of over-fitting the observed data. We used a specific machine learning algorithm known as 'dynamical factor graphs' [25] with an additional sparsity constraint on the gene regulation network. Interestingly, the coherence of the generated regulatory model is good enough that it is able to predict the direction of gene change (up-regulation or down-regulation) on future data points. This coherence allows us to propose a gene influence network involving transcription factors and 'sentinel genes' involved in the primary NO_3_^- ^response (such as NO_3_^- ^transporters or NO_3_^- ^assimilation genes). The role of a predicted hub in this network is evaluated by over-expressing it, and indeed leads to changes in the NO_3_^-^-driven gene expression of sentinel genes. The initial gene response to NO_3_^- ^is also analyzed and discussed for its insights into molecular physiology.

## Results and discussion

### Molecular physiology: assessing molecular reprogramming preceding the 'primary' nitrate response

**Figure 1:**
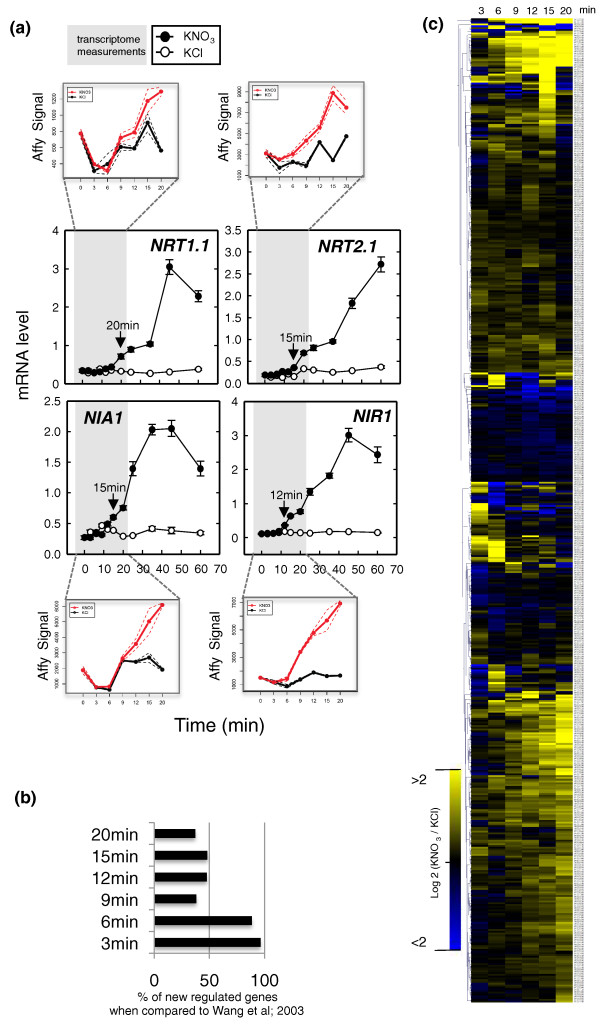
**High-resolution kinetics of transcriptome responses to NO**_**3**_^- ^**treatment**. **(a) **Levels of mRNA for nitrogen-responsive sentinel genes in *Arabidopsis *roots in response to NO_3_^- ^treatment. Fourteen-day-old plants grown in the presence of ammonium succinate were treated with 1 mM KNO_3 _or KCL (as a mock treatment). Plants were collected at 0 minutes (before treatment) and 3, 6, 9, 12, 15, 20, 25, 35, 45, and 60 minutes after treatment. Sentinel transcripts were measured in RNA from roots using RT-QPCR and normalized to two housekeeping genes (see Materials and methods). The insets show the Affymetrix MAS5 normalized signal for the sentinel genes on the 0- to 20-minute samples. The data represent the mean ± standard error of three and two biological replicates for QPCR and Affymetrix measurements, respectively. **(b) **Percentage of genes not detected as NO_3_^- ^regulated in Wang *et al*. [6]. **(c) **Overall behavior (relative expression) of 550 regulated genes (Log base 2(Signal KNO_3_/Signal KCl)) between 0 and 20 minutes. These data correspond to ATH1 measurement of the samples collected for the RT-PCR presented in (a) (grey shades; see also Materials and methods for further details).

To investigate genomic responses that precede the response of sentinel 'primary NO_3_^- ^response' genes (*NIR*, *NRT2.1*, *NIA1*, *NIR1*) to nitrate application, we first generated several time-series experiments (data not shown). These allowed us to identify the earliest time at which we were able to detect unambiguous NO_3_^- ^induction of these sentinel response genes using real time quantitative PCR (RT-QPCR). Figure 1a shows the expression of selected sentinel genes over time (0, 3, 6, 9, 12, 15, 20, 25, 35, 45, 60 minutes) in response to treatment with 1 mM KNO_3 _or controls of 1 mM KCl. These results (Figure 1a) demonstrate that a sentinel gene such as *NRT1.1 *is induced at 20 minutes (compared to KCl controls, and in comparison to gene expression at time 0 minutes). The timing of induction of other sentinel genes involved in the 'primary NO_3_^- ^response' are *NIR1 *at 12 minutes and *NRT2.1 *and *NIA1 *at 15 minutes. Following these preliminary experiments, we next ran Affymetrix ATH1 chips on biological replicates corresponding to the beginning of sentinel gene induction and their preceding time points (0, 3, 6, 9, 12, 15, 20 minutes). Note that we kept the 20-minute time point as a reference, since it was the earliest time point that had previously been studied [6].

**Figure 2:**
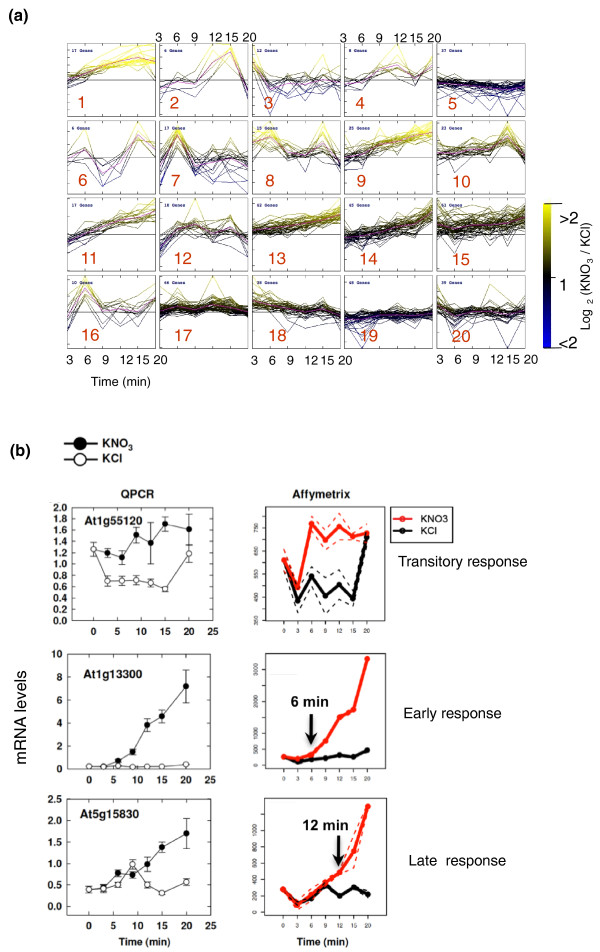
**Clustering analysis and QPCR reveals different patterns of expression in response to short-term NO**_**3**_^- ^**treatment**. **(a) **Cluster analysis of the relative expression of 550 regulated genes (Log base 2(Signal KNO_3_/Signal KCl)) between 0 and 20 minutes. These data correspond to ATH1 measurement of the samples collected for the RT-PCR shown in Figure 1 (see Materials and methods for further details). For clusters including genes with a significant over-representation of biological functions see Additional file 4. **(b) **Examples of three different gene behaviors (transitory, early, late responses) after NO_3_^- ^provision.

The resulting nitrate-responsive transcriptome kinetic dataset corresponded to 26 ATH1 chips with 22,810 probes each. A sequential analysis involving linear modeling (detailed in Materials and methods) was carried out to identify genes regulated at each particular time point with highly stringent criteria (including control of the false discovery rate (FDR)). We detected 83, 192, 55, 149, 190, and 229 genes significantly regulated by nitrate treatment at the 3, 6, 9, 12, 15 and 20 minute time points, respectively (Additional file 1). The union of these gene lists corresponds to 550 distinct nitrate-responsive genes. We demonstrate that a large majority of the newly identified NO_3_^-^-regulated genes are controlled at the earliest time points (3 and 6 minutes), which have never before been assayed (Figure 1b). In order to support these new findings, 15 genes have been validated by QPCR (Additional file 2) on three replicates (two were used for the microarray chips and one for QPCR only). The predicted behaviors of these genes were validated by the QPCR approach, as follows. One set of genes is shown to have a transient response to NO_3_^- ^(for example, *At1g55120*, *At3g50750*, *At1g64370*, *At4g16780*, *At1g27900*, *At1g22640*, *At1g52060*, and *At2g42200*). While a second gene set is validated to be very early responsive genes (for example, *At1g13300*, *At1g49000*, *At4g31910*, *At5g15830*, *At2g27830*, *At3g25790*, and *At5g65210*). Quantitatively, the correlation between the NO_3_^- ^induction (KNO_3_/KCl ratio) detected by both approaches (ATH1 chip and QPCR) is R^2 ^> 0.5 for 8 genes, 0.5 > R^2 ^> 0.4 for 3 genes, R^2 ^< 0.4 for 4 genes. It is noteworthy that for the genes having a low correlation, their overall behavior is validated by QPCR (for example, constant versus transient induction by NO_3_^-^; Figure 2b; Additional file 2).

To probe the biological significance of these kinetic patterns of nitrate regulation of gene expression, we determined the functional categories that are over-represented in the lists of nitrate-regulated genes at each time point, separating the induced and repressed gene lists (Additional file 2). Interestingly, the biological functions induced earliest after nitrate addition do not concern nitrogen directly. Instead, within 3 minutes, the very first statistically significant over-represented functional category is ribosomal proteins (*P*-value 6.58e^-6^). This finding generates the hypothesis that nitrogen could trigger a transient and very rapid reprogramming of key elements of the translation machinery needed to synthesize new proteins required for nitrogen acquisition. This idea might be further supported by the fact that many more genes are induced by the addition of nitrate than are repressed (see below). Moreover, later on in the time-course (as early as 9 minutes), the next biological function to be significantly induced is the oxidative pentose-phosphate-pathway, a function that is known to be a critical step providing reductants needed to assimilate NO_3_^- ^[26]. The oxidative pentose-phosphate-pathway has also been shown to generate a signal controlling key effectors of the NO_3_^- ^response, such as *NRT2.1*, *NRT2.4*, *NRT1.1*, *NRT1.5*, and *AMT1.3 *[27]. Taken together, these observations suggest that the early nitrate response involves mechanisms needed to prepare the plant to respond to nitrate rather than mechanisms that relate directly to nitrogen. Such mechanisms - for example, nitrate transport and amino acid metabolism - are regulated later on in the time series (Additional file 3).

To begin to decipher the pattern of nitrate-regulated gene expression over the entire time series, we first clustered the gene expression ratio (Log_2_(Signal KNO_3_/Signal KCl) of the 550 significantly regulated genes) in order to gain insight into the genomic reprogramming during the first 20 minutes of KNO_3 _treatment (Figure 1c). The vast majority of the reprogramming is an induction of gene expression by NO_3_^-^, rather than a repression. To quantify this observation, the numbers of genes that are detected as significantly induced by NO_3_^- ^at 3, 6, 9, 12, 15, and 20 minutes are 63 (76% of regulated genes), 146 (76% of regulated genes), 54 (98% of regulated genes), 123 (82% of regulated genes), 164 (87% of regulated genes), and 209 (92% of regulated genes), respectively. One interpretation is that NO_3_^- ^induces an adaptation program that is on 'stand-by' in NO_3_^-^-free conditions, rather than a shut-down of a putative 'N-free-condition' program. Clustering analysis also allowed us to sort gene responses according to their overall behavior. This analysis demonstrated that rapid gene expression responses to nitrate could be classified into up to 20 clusters (according to figure of merit (FOM) analysis; see Materials and methods; Figure 2). Considering each cluster independently, we were able to identify over-represented biological functions for eight clusters, including chloroplast, the oxidative pentose-phosphate-pathway, and ribosomal proteins (Figure 2; see Additional file 4 for details).

Moreover, we identified and analyzed 146 genes that were consistently induced over the 20 minutes of nitrate treatment (corresponding to clusters 1, 9, 11, 13, and 14). This group of consistently nitrate-induced genes includes over-represented biological functions such as oxidoreduction coenzyme process (*P*-value = 0.00027), nicotinamide metabolic process (*P*-value = 6.50e-05), regulation of transcription (*P*-value = 0.00167), pentose phosphate shunt (*P*-value = 0.00073). We also identified 219 genes showing responses to nitrate that seem to represent a general pattern of transient regulation (clusters 2, 3, 4, 6, 7, 8, 10, 12, 16, 17, and 18). Interestingly, the oxygen and redox state of the cell seems to be a general function that is transiently adapted by KNO_3 _treatment. Indeed, Munich Information Center for Protein Sequence (MIPS) functions such as oxygen radical detoxification (*P*-value = 0.00018), peroxidase reaction (*P*-value = 0.01479), and superoxide metabolism (*P*-value = 0.02472) are over-represented gene ontology terms in this group. This observation might indicate the effect of NO_3_^- ^on the redox state of the cell. Finally, we show that 124 genes are repressed by NO_3_^- ^treatment, transiently or otherwise (corresponding to clusters 5, 19, and 20). The common function overrepresented in this group is transcription (*P*-value = 0.00312). This could result from the extinction of the pre-existing transcriptome program preceding the NO_3_^- ^treatment. Since the plants had been nitrogen starved for 24 hours before NO_3_^- ^treatment, this might correspond to genes that are up-regulated by the pre-treatment (nitrogen starvation) and down-regulated by NO_3_^- ^provision. To statistically test this hypothesis, we set up a randomization test (see Materials and methods) to quantify whether the genes that are down-regulated in our conditions correspond to genes that were up-regulated by nitrogen starvation in Peng *et al*. [28]; this occurred with a *P*-value of 0.0089. Conversely, no significant overlap was detected for clusters induced bi NO_3_^- ^(clusters 1, 2, 4, 9, 10, 11, 13, 14). This finding validates the idea that NO_3_^-^-down-regulated clusters correspond to genes involved in the response of plants to the pre-treatment conditions. In summary, a large part of the NO_3_^- ^gene expression reprogramming has been missed by previous genomic studies. The time-varying expression modulation newly identified here involves physiological functions that could be components of the nitrate signaling system itself.

**Figure 3:**
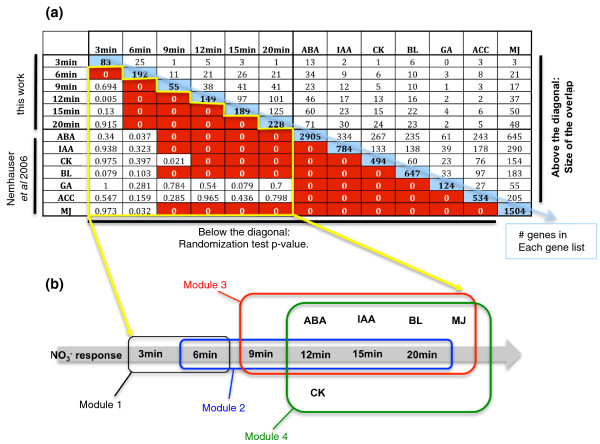
**Identification of NO**_**3**_^- ^**response and hormonal cross-talk modules**. **(a) **For each pair of gene lists (NO_3_^- ^responsive (this work) or hormone responsive [29]), a *P*-value (randomization test; see Materials and methods) was computed and is shown in the table below the blue diagonal. Entries in the blue diagonal give the gene list size in number of genes. Above the diagonal the size of the intersection of each pair of studied gene lists is given. Note that *P*-value = 0 means *P*-value < 0.001. Analysis of the *P*-values included within the yellow outline led to the building of gene modules depicted in the conceptual model provided in **(b)**. ABA, absisic acid; ACC, 1-aminocyclopropane-1-carboxylic-acid (ethylene precursor); BL, brassinolides; CK, cytokinins; GA, gibberellic acid; IAA, indole acetic acid (auxin); MJ, methyl jasmonate.

In order to further document the potential of this dynamic transcriptome response to mediate cross-talk between nitrate signaling and other well-studied signaling pathways in plants, we evaluated if the gene sets regulated by NO_3_^- ^at the different time points in our analysis overlap more than expected by chance with genes regulated by hormones using data generated by the Chory lab [29]. To do this, we compared the nitrate-regulated gene lists (over six time points) with the lists of hormone-regulated genes [29] and generated a matrix that assembled the randomization test *P*-values (see Materials and methods) between each pair of gene lists. The lists included genes regulated by NO_3_^- ^across each of the six time points (our study), and lists of genes regulated by seven different hormones by the Chory lab (abscisic acid, cytokinins, auxin (IAA), methyl jasmonate, brassinolides, gibberellic acid, ethylene)] [29]. These results (Figure 3) lead to three main conclusions supporting the existence of gene modules responding to nitrate and hormone signaling.

First, we considered only the overlap between the NO_3_^-^-responsive gene lists at different time points. We found evidence for two linked 'modules' of nitrate-regulated gene expression (modules 1 and 2 in Figure 3b). The first nitrate-regulated module consists of the nitrate-regulated genes in the union of the 3- and 6-minute gene lists. The overlap between these two lists is far beyond what we would expect by chance (*P*-value < 0.001). However, the 3-minute gene list overlaps very little with the rest of the nitrate-regulated genes in the time-course study. As such, the second nitrate-regulated module is made up of the union of the 6-, 9-, 12-, 15-, and 20-minute gene lists (these gene lists overlap significantly more than random). The 6-minute gene list acts as the link between the very early nitrate-response genes (before 6 minutes) and the more delayed ones (after 6 minutes).

Second, the overlap of the nitrate-regulated genes with the hormone-regulated genes (modules 3 and 4 in Figure 3b) is significantly higher than expected at the 9-minute nitrate time point for abscisic acid-, indole acetic acid-, brassinolide- and methyl jasmonate-regulated genes, while the 12-minute nitrate time point overlaps significantly with cytokinin-regulated genes. This suggests that the interaction of nitrate signaling with other hormone signals is likely to involve the genes regulated by nitrate after 9 minutes. This leads to the hypothesis that, from 0 to 6 minutes, the genomic reprogramming concerns a pure NO_3_^- ^signaling pathway, and thereafter (for example, 9 minutes after nitrate treatment) interactions with developmental signals such as hormones occur (Figure 3). This enables us to derive the hypothesis that the early nitrate controllers (for example, transcription factors, kinases, and so on) regulated at 3, 6, and 9 minutes are involved in the control of the nitrate signaling itself, rather than in the interaction between NO_3_^- ^and other signals such as hormones.

Third, this analysis shows that the different hormonal treatments control largely overlapping gene modules, as has been described previously [29].

In conclusion, connections between NO_3_^- ^and hormone-related signaling are common features of plant molecular networks at several layers of integration (for a review, see [30]). For instance, transcriptional connections have been identified where genes involved in a NO_3_^-^-responsive 'biomodule' have been shown to be more responsive to NO_3_^- ^if they are also strongly regulated by hormones [13]. More recently, we provided a mechanistic hypothesis to explain the role of NRT1.1 as a NO_3_^- ^sensor controlling lateral root development. Indeed, NRT1.1 is a transceptor able to transport both auxin and nitrate. The sensing mechanism results from the ability of nitrate to inhibit auxin transport by NRT1.1, leading to low lateral root development at low nitrate concentrations [16]. To determine whether this mechanism is also involved in the transcriptional induction studied in the present work will require further investigation. However, the fact that hormones can be involved at the beginning of NO_3_^- ^sensing mechanisms [13,16] and downstream of NO_3_^- ^transcriptional activation (this analysis) is an intriguing observation that deserves further investigation to understand what is the purpose of such signal entanglement.

### Machine learning approach: modeling of regulatory gene influences through predictive models

#### Dynamical predictive modeling of regulatory gene networks

Time-series datasets of gene expression levels, as measured by microarrays, can provide us with a detailed picture of the behavior of the genetic network over time, but they contain this information in a highly noisy form requiring reverse engineering [31]. An additional challenge of systems biology is to be able to model systems precisely enough that they can predict untested conditions, especially given the paucity of data relative to the number of possible connections.

Among the several approaches to this modeling problem, dynamical models have gained prominence as they simultaneously encode the topology of the gene interaction graph and its functional evolution model. Such a model can in turn be used for predictive modeling of gene expression at later time points or upon perturbation. Such dynamical models essentially consist of a mathematical function that governs the transitions of the state of a gene regulatory network over time. Typically, dynamical models of mRNA concentrations consist of ordinary differential equations (ODEs) [31]. For a given gene *i*, ODEs can, for instance, define the rate of change of mRNA concentration *y*_*i*_(*t*) (with a kinetic constant *τ*), as a function *g*_*i *_of the influences of transcription factors (which we assume in this article to consist of the vectors **y**(*t*) of all observed mRNA measures, because protein levels are unavailable to us), with an optional mRNA's degradation term, as in the equation below:

$$\tau \frac{\text{d}y_{i}(t)}{\text{d}t}=g_{i}(\text{y}(t))-y_{i}(t)$$

In our study, we have considered dynamics with the mRNA degradation term (the so-called 'kinetic' model [32,33]) and without it (the so-called 'Brownian motion' model [34]). Assuming degradation (kinetic ODE) worked better.

Since microarray data are discretely sampled over time, the above equation is linearized; hence, it explains how gene expressions at time *t *influence gene expressions at time *t *+ 1.

In our study, the sequence of microarrays contained seven full-genome mRNA measures (with two replicates) at 0, 3, 6, 9, 12, 15 and 20 minutes; in the cross-validation leave-out-last study, we used measures between 0 and 15 minutes to fit the model for each gene *i *(by tuning the parameters of associated dynamical functions), and tested the fitted model on the last time point (prediction of the mRNA level at 20 minutes).

#### Choosing the model

In a review article, Jaeger and Monk [31] pointed out that the inference of biological networks in the presence of few time-point measurements, many genes, measurement errors and random fluctuations in the environment is inherently difficult. Because of this limitation, methods for computational inference of gene regulation networks can be crudely divided into two approaches: non-linear or state-space based modeling of the complex interactions between a restricted number of genes (typically ten) with hidden protein transcription factors; or simpler, but linear, models of transcription factor-gene interactions [32-35], relying on larger (hundreds to thousands) numbers of microarray measurements.

State-space models (SSM) are a general category of machine learning algorithms that model the dynamics of a sequence of data by encoding the joint likelihood of observed and hidden variables. A popular probabilistic example of SSMs that have been applied to gene expression data are dynamical bayesian networks [36], such as linear dynamical systems [37,38]. SSMs assume an observed sequence **y**(*t*) (in our case, gene expression data) to be generated from an underlying unknown sequence **z**(*t*), also called 'hidden states'. Consecutive hidden states form a Markov chain {**z**(0), **z**(1), ..., **z**(T-2), **z**(*T*-1)} (in our case, the sequence contains seven states at 0, 3, 6, 9, 12, 15 and 20 minutes); each transition in the chain corresponds to the same stationary (that is, time invariant) dynamical model *f*.

As a first example of complex SSMs, Zhang *et al*. used gaussian processes dynamical models with nonlinear dynamics to infer the profile of a single transcription factor (the tumor suppressor p53) and explained the activity of a large collection of genes using that transcription factor only (without any other transcription factor-gene interaction) [39]. Another example is the linear dynamical system, which Beal *et al*. [37] as well as Angus *et al*. [38] used to infer the profiles of 14 hidden transcription factors for 10 observed genes only, either without predictive cross-validation [37], or on synthetically generated data [38].

Examples of first-order linear dynamical models for gene expression include the Inferelator by Bonneau *et al*. [32,33]. The Inferelator consists of a kinetic ODE that follows the Wahde and Hertz equation [40] and where transcription factors contribute linearly. This ODE also includes an mRNA degradation term. Some instances of the Inferelator introduce nonlinear AND, OR and XOR relationships between pairs of genes, based on a previous bi-clustering of genes. One has to note that the Inferelator has been mostly applied to datasets with hundreds of data-points (for example, *Halobacterium*).

Other examples include the first-order vector autoregressive model VAR(1) [35] and the 'Brownian motion' model (which is a VAR(1) model of changes in mRNA concentration) [34]. Lozano *et al*. [41] suggested using a dynamic dependency on the past 2, 3, or 4 time points, but this was impractical in our case given the relatively small number of microarray measurements in our experiments.

Two microarray replicates were acquired in this study. Since each replicate is independent of all microarrays preceding and following in time, there were four possible transitions between any two time points *t *and *t *+ 1, and we therefore used four replicate sequences to train the machine learning algorithm.

#### A noise reduction approach to state-space modeling of regulatory gene networks

In a departure from previous SSM frameworks, our noise-reduction approach uses the hidden variables to represent an idealized, 'true' sequence of gene expressions **z**(*t*) that would be measured if there were no noise. The set of all genes at time *t *is modeled by a 'latent' (that is, hidden but correct) variable (denoted **z**(*t*)), about which noisy observations **y**(*t*) are made.

**Figure 4:**
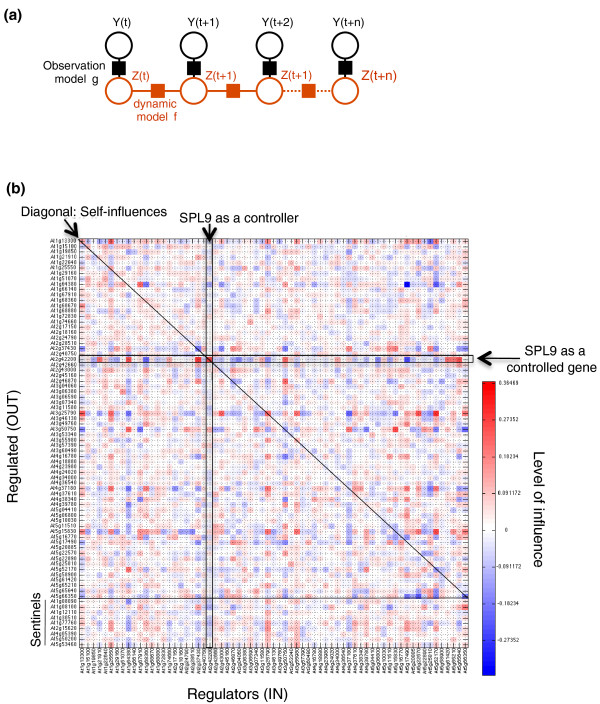
**State space modeling predicts transcription factor influence**. **(a) **Conceptual scheme of the state space modeling. An unknown function f (red square) relates the values of latent variables Z(t) and Z(t + 1) (for all t) corresponding to consecutive time measurements. Learning algorithms iteratively optimize the function f mapping latent values of transcription factors to changes to target genes (and transcription factors themselves at time t + 1). **(b) **The whole dataset (from 0 to 20 minutes of KNO_3 _treatment) has been learnt by state space modeling (validated to be predictive in a leave-one-last approach; Table 2). The resulting *f *function has learnt possible connections and can be displayed as an influence matrix. SPL9 is a transcription factor predicted to be a potential bottleneck and is further experimentally studied.

Specifically, we a) model the dynamics on hidden states **z**(*t*) instead of modeling them directly on the Affymetrix data **y**(*t*), as well as b) have the hidden sequence **z**(*t*) generate the actual observed sequence **y**(*t*) of mRNA, while incorporating measurement uncertainty. Such an approach has been used in robotics to cope with errors coming from sensors. Our proposed SSM is depicted in Figure 4a, where each node **y**(*t*) or **z**(*t*) represents a vector of all gene expressions at a particular time point, and where latent variables are represented by large red circles, and observed variables by large black circles.

Our goal is to learn the function *f *that determines the change in expression of a target gene *z*_*j*_, as a linear combination of the expression of a relatively small number of transcription factors, and that relates the values of latent variables **z**(*t*) and **z**(*t *+ 1) corresponding to consecutive time measurements (function *f *is represented by a red square in Figure 4a). The relationship between latent and observed variables is assumed to be the identity function *h *with added Gaussian noise (represented by a black square in Figure 4a).

The function *f *is modeled as a linear dynamical system (that is, a matrix **F**). This linear Markovian model, which represents a kinetic (RNA degrades) or Brownian motion (RNA does not degrade) ODE, is the simplest and requires the fewest parameters (there is one parameter per transcription factor-gene interaction, and an additional offset for each target gene). This model thus helps to avoid over-fitting scarce gene data. The linear model operates on hidden variables, which become a smoothed version of the observed gene expression data.

Because our noise reduction state-space modeling algorithm is efficient, simple and tractable, as explained in the Materials and methods section, it can handle larger numbers of genes (we focused on 76 genes) than other SSM approaches, given enough genes [37-39].

#### Comparative study of state-space model optimization

Out of the 550 nitrogen-regulated genes, we extracted 67 genes that correspond to all the predicted transcription factors and 9 N-regulated target genes that belong to the primary nitrogen assimilation pathway. The transcription factors have been used as explanatory variables (inputs to *f*) as well as explained values (output from *f*) (Figure 4b), whereas the nitrogen assimilation target genes are only explained values. We then optimized our SSM, using different algorithms, in order to fit it to the observed data matrix, and compare all our results in Table 1. We also compared our SSM approach to non-SSM approaches [32-35,42,43] (Table 2).

**Table 1:** The kinetic ODE and both the conjugate gradient and LARS optimization algorithms obtain the best fit to the 0 to 15 minutes data, with good leave-out-last predictions

			Best hyperparameters (with respect to SNR on leave-1 training dataset)			Performed on training set:	Performed on test set:
Dynamics	Normalization	Optimization	Gamma (state-space coefficient)	Tau (kinetic time constant)	Lambda (regularization parameter)	SNR (in dB) on leave-1 training dataset	percentage of correct signs on leave-1 test dataset
Kinetic	MAS5	Gradient	1	3	0.0001	32.4	68%
Kinetic	MAS5	LARS	0.1	3	0.1	32.4	74%
Kinetic	MAS5	Elastic Nets	0.1	7	0.05	32.2	71%
Brownian	MAS5	Gradient	0.1	NA	0.0001	32.1	65%
Brownian	MAS5	LARS	0	NA	0.05	32.1	63%
Brownian	MAS5	Elastic Nets	0	NA	0.05	32.1	63%
Naïve trend prediction	MAS5		NA	NA	NA	NA	52%

Each line in the table represents the type of ODE for the dynamical model of transcription factor-gene regulation (either kinetic, with mRNA degradation, or 'Brownian motion', without mRNA degradation), the type of microarray data normalization, and the optimization algorithm for learning the parameters of the dynamical model. For each of these, we selected the best hyperparameters, namely the state-space coefficient gamma, the kinetic time constant (in minutes) and the parameter regularization coefficient lambda, based on the quality of fit to the training data (from 0 to 15 minutes), as measured by the signal-to-noise ratio (SNR), in dB. We then performed a leave-out-last (leave-1) prediction and counted the number of times the sign of the mRNA change between 15 minutes and 20 minutes was correct. We compared these results to a naïve extrapolation (based on the trend between 12 and 15 minutes) and obtained statistically significant results at *P *= 0.0145.

**Table 2:** The quality of fit of our state-space model approach slightly outperforms the non-SSM approaches

			Best hyper parameters (with respect to SNR on leave-1 training dataset)			Performed on training set:	Performed on test set:	
Dynamics	Normalization	Optimization	Gamma (state-space coefficient)	Tau (kinetic time constant)	Lambda (regularization parameter)	SNR (in dB) on leave-1 training dataset	percentage of correct signs on leave-1 test dataset	Reference
Kinetic	MAS5	Gradient	1	3	0.0001	32.4	68%	This work
Kinetic	MAS5	LARS	0.1	3	0.1	32.4	74%	This work
Kinetic	MAS5	LARS	0	3	0.05	32.1	74%	[33]
kinetic	MAS5	Elastic Nets	0	3	0.05	32.1	74%	[35]
Brownian	MAS5	Gradient	0	NA	0.005	32.1	66%	[34]
Brownian	MAS5	LARS	0	NA	0.05	32.1	63%	[34]
Brownian	MAS5	Elastic Nets	0	NA	0.05	32.1	63%	[34]
Naïve trend prediction	MAS5		NA	NA	NA	NA	52%	

We compared our SSM-based technique (with a non-zero SSM parameter gamma) to previously published algorithms for learning gene regulation networks by enforcing gamma = 0 (see Materials and methods). We notice that the LARS algorithm [42], used in the Inferelator by Bonneau *et al*. [32,33], as well as Elastic Nets [35,43], obtain a slightly worse quality of fit (signal-to-noise ratio (SNR), in dB) than when combined with our state-space modeling for the same leave-out-last (leave-1) performance as our SSM plus LARS. Not using an mRNA degradation term, as in Wang *et al*. [34], degrades the leave-out-last performance.

Iterative learning algorithms, described in this study, alternate between two steps: learning the function *f *mapping latent values of transcription factors at time *t *to changes to target genes (and transcription factors themselves) at time *t *+ 1; and recomputing (inferring) the values of the latent variables. In the first step, learning the function *f *corresponds to finding parameters of **F **that minimize the prediction error and that involve few transcription factors, thanks to a sparsity constraint on **F**. In the second step, the sum of quadratic errors on functions *f *and *g *is minimized with respect to latent variables **z**(*t*) by gradient descent in the hidden variable space [25]. The learning procedure is repeated (learning model parameters, inferring latent variables) on training data until **F **stabilizes (see Materials and methods). Using a bootstrapping approach based on random initialization of latent variables **z**(*t*), we further repeat the SSM iterative procedure 20 times and take the final average network **F **(see Materials and methods).

Three hyper-parameters were explored in our learning experiments: the kinetic time constant *τ *(unless the ODE was 'Brownian motion'), the amount of L1-norm regularization *λ *(explained in Materials and methods), and a variable *γ *linked to the SSM.

When the state-space coefficient is *γ *= 0, we can recover non-SSM algorithms: LARS (least-angle regression and shrinkage) [42], as used for instance by Bonneau *et al*. [32,33] and Elastic Nets [43], as used, for instance, by Shimamura *et al*. [35]. LARS is a fast implementation of Tibshirani's popular LASSO (least absolute shrinkage and selection operator) regression with L1-norm regularization [44]. Elastic Nets are an improvement over LARS and LASSO, and their main advantage is to group variables (in our case genes) as opposed to choosing one gene and leaving out correlated ones. Moreover, if we do not use the mRNA degradation term in the kinetic ODE, and use instead 'Brownian motion' dynamics, and if we set the state-space coefficient to *γ *= 0, we recover an approach comparable to the one published by Wang *et al*. [34] (although their optimization algorithm was based on the singular value decomposition (SVD) of the microarray data).

For each type of ODE (kinetic or 'Brownian motion') and type of optimization algorithm, we exhaustively explored the space of hyper-parameters (*τ*, *γ*, *λ*) in order to optimize the quality of fit of each model to the first six time points (0, 3, 6, 9, 12 and 15 minutes). We repeated the experiment using two different ways of normalizing microarray gene expression data: MAS5 and RMA (Robust Multi-array Average). Interestingly, it appears that machine learning (ML) approaches better fit MAS5 data (32.4 db) compared to RMA data (30.8 db). Thus, the study was continued on MAS5 normalized data, as it was for the vast majority of the studies that we reviewed in this work. As can be seen in Table 1, we identified the SSM relying on the kinetic ODE, and with either LARS or conjugate gradient optimizations, as the two best (having the highest signal-to-noise ratio) optimization algorithms on the MAS5 training datasets. The signal-to-noise ratio is a monotonic function of the normalized mean square error on the predicted values of mRNA; all algorithms used in this article aim at minimizing the normalized mean square error, that is, at maximizing the signal-to-noise ratio.

Having chosen the two best algorithms using all time points up to and including 15 minutes as training data, we performed a 'leave-out-last' test, consisting of predicting both the direction and magnitude of the change of the gene expression states between 15 and 20 minutes. Using those algorithms with those parameter settings, we made predictions about whether gene expression levels would be increased (positive sign) or decreased (negative sign) at 20 minutes compared with at 15 minutes.

As Table 1 shows, a SSM relying on the kinetic ODE and with LARS optimization (kinetic LARS) gives correct results 74% of the time on a set of 53 genes (47 transcription factors and 6 nitrogen assimilation genes) that are 'consistent' among the two biological replicates in their behavior (consistently up- or down-regulated in both replicates) for the transition from 15 minutes to 20 minutes. When we considered all 76 genes, regardless of their 'consistency' across replicates, kinetic LARS still gave correct results 71% of the time. The other chosen algorithm (kinetic ODE with conjugate gradient optimization), yielded 68% correct results on both the 53 consistent genes and on all 76 genes. By contrast, a naïve 'trend forecast' algorithm to extrapolate the trend between 12 minutes and 15 minutes was correct for only 52% of the consistent genes, just slightly better than random (this result implies that 48% of the consistent genes changed 'direction' at 15 minutes). Thus, our SSM does significantly better (*P*-value = 0.0145) than the naïve trend forecast based on a binomial test (see Materials and methods) on a coin that is biased to be correct 52% of the time.

Using the hyper-parameters (*τ*, *γ*, *λ*) corresponding to the two best solutions (kinetic LARS and kinetic conjugate gradient), we retrained two SSMs on all the available data (0 to 20 minutes) to obtain corresponding gene regulatory networks. Finally, we performed a statistical analysis of the bootstrap networks in order to retain transcription factor-gene links that were statistically significant at *P *= 0.001 (see Materials and methods). We ultimately selected the conjugate gradient-optimized network as it gave a less sparse solution (394 links) than the LARS-optimized gene regulatory network (GRN) (22 links). We used this network (next section) to analyze the NO_3_^- ^response of sentinel genes to transcription factors.

We are confident in dynamical modeling, and in our SSM in particular, because in the leave-out-last tests, we were able to learn the system well enough to predict the direction of changes to gene expression. This suggests that we might have learnt some consistent and biologically meaningful networks involved in NO_3_^- ^response pathway. Since function *f *models the gene regulation network learned during the leave-out-last test, we conclude by presenting the function *f *obtained from the full time sequence 0 to 20 minutes. This function *f *can be displayed as an influence matrix (Figure 4b). The study of this network/matrix as a whole system is discussed below.

### Over-expression of a potential network hub (SPL9) modifies the NO_3_^- ^response of sentinel and transcription factor genes

**Figure 5:**
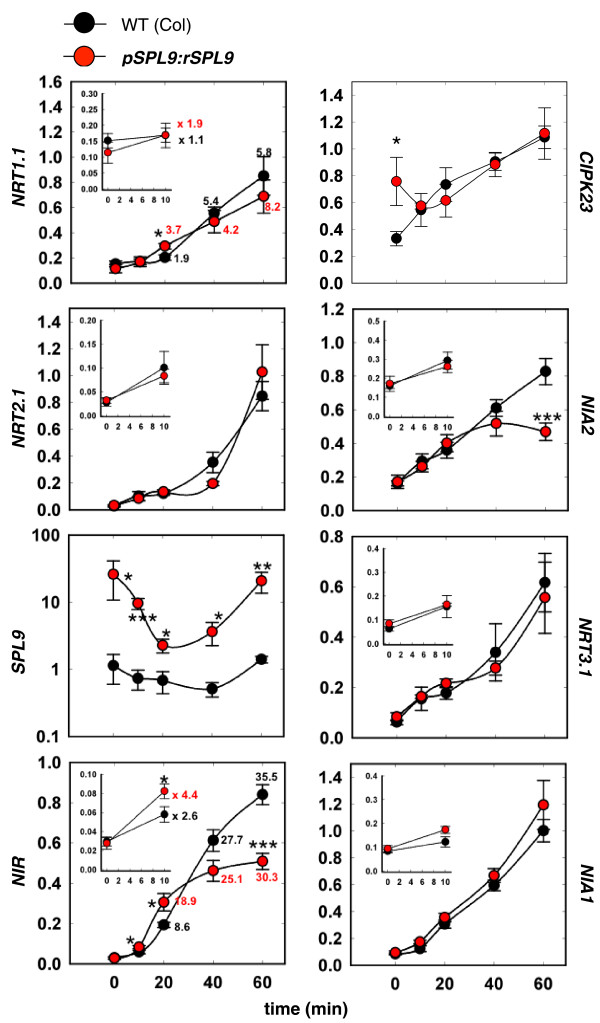
**rSPL9 over-expression modifies NO**_**3**_^- ^**kinetic responses**. Sentinel gene mRNA levels in roots of wild type (WT) and transgenic pSPL9:rSPL9 plants in response to NO_3_^- ^treatment. Fourteen-day-old plants grown in ammonium succinate were treated with 1 mM KNO_3 _versus KCl. Roots were collected at 0 minutes (before treatment) and 10, 20, 40, and 60 minutes after the treatment. Sentinel transcripts were measured in roots using RT-QPCR and normalized to two housekeeping genes (see Materials and methods). The data represent the mean ± standard error of three biological replicates (three independent experiments). Differences between the two genotypes are statistically significant at *P < 0.05; **P < 0.01 and ***P < 0.001 (*t*-test). When a gene presents significant differences, the NO_3_^- ^induction ratio compared to time 0 is indicated by the numbers close to the plots. The inserts display a zoom of the early time points. More genes (transcription factors) are displayed in Additional file 5.

In order to probe the role of a transcription factor/hub in the predicted regulatory network presented in Figure 4b, transgenic plants (pSLP9:rSPL9) expressing an altered version of the mRNA for the SPL9 transcription factor were compared to wild-type plants for their response to NO_3_^- ^provision. This gene has been selected for several reasons: (i) it is induced at very early time points (3 and 6 minutes); (ii) the inferred network predicts that SPL9 potentially controls at least six genes, including two nitrogen assimilation sentinel genes - this places it as the third-most influential transcription factor on the nitrogen assimilation gene sentinels; (iii) it is also the most strongly influenced gene in both its number of 'in' connections, and by the magnitude of the regulations controlling it (Figure 4b); and (iv) SPL9 displays some strong correlations with key regulators across the NASC (Nottingham Arabidopsis Stock Centre) array dataset (see below). As such, SPL9 constitutes a potential crucial bottleneck in the flux of information mediated by the proposed nitrogen regulatory network (Figure 4). We first considered SPL9 mutants and monitored sentinel expression in this genetic background. However, even if some defects have been observed, no consistent phenotype can be reported (data not shown). This may be readily explained by the topological redundancy of the network (Figure 4). Thus, one could expect that over-expression of *SPL9 *mRNA would trigger a detectable effect on the predicted sentinel targets and on the network behavior. SPL9 is a miR156-targeted SBP-box transcription factor identified to control shoot development [45] and flowering transition [46,47], and it also appears as a potential central regulator in the network derived from the SSM (Figure 4). SBP-box transcription factors are potentially redundant, but the use of miR156-resistant SPL9 transgenic plants (resulting in over-expression/gain of function plants) has been extensively used to decipher their role. In our experimental set-up, transgenic *SPL9 *mRNA is over-expressed 4- to 20-fold in the miR156-resistant plants compared to wild type (Figure 5). Moreover, although the *rSPL9 *mRNA resulting from the modified gene is resistant to degradation by miR156 (explaining the over-expression) the transgene is still under the control of its native promoter. Thus, this over-expression reflects, at least in part, the promoter activity or the effect of other post-transcriptional controls (independent of miR156). This demonstrates that the *SPL9 *promoter activity is potentially also under the effect of NO_3_^-^, since *rSPL9 *mRNA exhibits a transitory depression in the transgenic plants (Figure 5).

mRNA transcription levels of several sentinel genes have been followed in this pSPL9:rSPL9 transgenic line. The most dramatic effect recorded is for the *NIR *target sentinel gene. Interestingly, the *NIR *gene has previously been demonstrated to be one of the most robustly NO_3_^-^-regulated genes based on a meta-analysis of microarray data from nitrogen treated plants [14]. In support of this, over-expression of the *SPL9 *gene leads to a significant advance in the *NIR *NO_3_^- ^response by about 10 minutes, and attenuates its magnitude of regulation at later time points (60 minutes). Less dramatic but still significant effects (over three independent experiments) have been recorded for *NRT1.1*/*CIPK23 *genes, belonging to the NO_3_^- ^sensing module [20], and for the *NIA2 *gene. These results demonstrate a role of the SPL9 transcription factor in the control of genes involved in the NO_3_^- ^primary response. We also further investigated the role of SPL9 over-expression on the transcription levels of genes in the network over time (Figure 4b). Interestingly, SPL9 seems to have an effect on the vast majority of the genes in the regulatory network that we have tested (Additional file 5). The diversity of the misregulation of gene expression is high. For instance, 4 out of the 14 genes tested display an early effect (between 0 and 20 minutes) after the SPL9 over-expression. However, 11 genes display modified gene expression at later time points (40 and 60 minutes).

An observation needs to be made at this point. We compared the predicted effect of SPL9 on its putative targets (inferred by ML), with the actual effect of over-expression of SPL9 on these genes measured by Q-PCR. The only systematic effect that we found is that genes predicted to be negatively regulated by SPL9 display an early induction in the pSPL9:rSPL9 line and are indeed less induced at later time points (this is an interesting feature found for four genes, including *NIR*, At1g13300, At5g10030, and At5g65210). However, the opposite is not true, since the genes predicted to be up-regulated by SPL9 also display a 'down-regulation' at later time points (by 60 minutes), but no 'up-regulation' at early time points (10 to 20 minutes), in response to rSPL9 over-expression. This relative absence of logic can be very easily explained by the predicted functional redundancy found in the network (also discussed below). The question about predicting the over-expression of a network hub is intriguing and will need further investigation, including the generation of transcriptome data to probe and learn how the whole system is perturbed by gene over-expression.

On the other hand, it is noteworthy that with its intrinsically highly connected topology, the network is able to amplify effects (such as SPL9 over-expression) across steps/time. Indeed, consider a simple positive feedback loop where A→B and also B→A; if the coefficient of A→B and B→A is only 10%, this is enough that when the expression of A and B is 100 at time 0, it will reach 160 after 5 steps. Here, we hypothesize that this is what happens for SPL9; that it takes time (more than 20 minutes) to amplify the differences recorded for the 11 genes with modified gene expression at later time points.

This analysis defined SPL9 as a potential hub for short time scale regulatory behavior, with small-amplitude regulatory effects (36% maximum). Those effects are going to be amplified across consecutive time steps and have longer-term (beyond 40 minutes) effects on additional genes. Thus, the interesting part of machine learning approach is that it can be used to predict the eventual behavior at time points that were not used in the ML process.

Interestingly, in our experimental setup, pSPL9:rSPL9 does not display any obvious developmental phenotype, contrary to what was described by Wang *et al*. [48]. These diverging results may be explained by the different plant growth conditions used in the two studies. In particular, in our pre-treatment conditions, plants were grown for 14 days in ammonium succinate without any NO_3_^-^. By contrast, in the Wang *et al*. studies the phenotypes were observed in plants grown on nitrate as a nitrogen source. The hypothesis that the phenotypes are nitrate-dependent is supported by the fact that the majority of the pSPL9:rSPL9 gene regulation phenotypes (Figure 5; Additional file 5) are triggered by NO_3_^- ^provision. Out of the 18 genes displaying a mis-regulation in the transgenic plants, only the *CIPK23 *gene displays a phenotype before nitrate treatment. For the 17 other genes, NO_3_^- ^is necessary to promote the rSPL9 over-expression effect.

In order to test the hypothesis that nitrate induces the phenotypes in transgenic plants, plants (wild type and pSPL9:rSPL9) were plated on the same background media used for the kinetic experiments (see Materials and methods) and complemented with 0.5 mM (NH_4_)_2_-succinate or 1 mM KNO_3_. Following 8 days after germination, different root traits were scored. These results show that indeed the presence of NO_3_^- ^enhances pSPL9:rSPL9 phenotypes (Additional file 6). Overall, only minor developmental differences were recorded on the (NH_4_)_2_-succinate media that cannot explain the distinct molecular phenotypes shown in Figure 5 and Additional file 5.

From a biological point of view, the modification of the nitrogen status of the plant (induced by nitrogen deprivation) has been shown to increase pri-miR156 accumulation [49] and mature miR156 in Hsieh *et al*. [50]. However, it is noteworthy that miR156 induction is not restricted to nitrogen deprivation, as phosphorus deprivation can also induce miR156 accumulation [50]. On the SPL9 side, we found that its expression across the Affymetrix NASC dataset is either positively or negatively correlated with key molecular actors in NO_3_^- ^sensing, metabolism and development (NRT1.1, -0.68; NRT1.2, -0.55; ARF8, 0.73). These results provide additional support for a role for the mir156/SPL9 partners in the control of the nitrogen response in plants and potentially in its coordination with other signals, such as phosphorus status.

### A highly complex connected network: causes and consequences?

Our machine learning approach (state-space modeling) proposes a regulatory network learned from a high-resolution dynamic transcriptome analysis made in response to KNO_3 _provision. A first interesting feature of this regulatory network is that it predicts a high level of connectivity (Figure 4b). Indeed, for the 76 studied genes, 60 have 500 significant connections (*P*-value < 0.001; see Materials and methods). This high level of connectivity (favoring functional redundancy) may explain why, to date, experimental analyses have uncovered only few molecular actors specifically involved in the control of NO_3_^-^-induced gene expression (NRT1.1, CIPK8, CIPK23, NLP7, LBD37/LBD38/LBD39) [2,20-23]. Moreover, this level of potential transcription factor redundancy can be a cause of the variable and conditional NO_3_^- ^genomic responses registered over different laboratories and discussed in Gutierrez *et al*. [14] and Krouk *et al*. [2].

### Predicted regulatory network influences

The identification of regulatory networks is a major aim of systems biology. Relatively few studies have determined regulatory networks precisely enough so that the model can predict behavior in untested conditions; the successful studies concern unicellular organisms such as *Halobacterium salinarum *[32,33]. Our regulatory network model is far more simple; it includes less than a hundred genes and is much less predictive than the one developed for *Halobacterium *since our model is fed only with transcriptomic data and vastly fewer experiments. Interestingly, however, both approaches predict rather low transcription factor influences. Indeed, the maximum predicted influence of one transcription factor on one gene of our model ranges between 10% and 30% (positive or negative influences). Low influence rate might reinforce the notion that functional redundancy is a built-in feature of regulatory networks that helps organisms adapt themselves to the context of interacting environmental and/or evolutionary forces. Also, the fact that *Arabidopsis *is a multicellular organism suggests a potential for some 'hidden' regulatory networks, as cell-specific studies have suggested [10]. This level of complexity, combined with the fact that genes in plants are not organized in functional clusters (as they are in bacteria), are likely to be some of the numerous reasons why our model is less predictive compared to that for *Halobacterium*. Thus, the next challenge of the plant systems approach will be to reach a level of understanding able to infer regulatory networks at several levels of integration [3].

### Comparative study of state-space model optimization versus non-state-space methods

In order to measure the improvement of our SSM over the non-state-space methods, given our *Arabidopsis *dataset, we also ran our SSM with the state-space coefficient set to *γ *= 0 for the following methods: 1) kinetic ODE with LARS optimization, as in Bonneau *et al*. [32,33]; 2) first-order vector-autoregressive model with Elastic Nets optimization, as in Shimamura *et al*. [35]; and 3) 'Brownian motion' ODE, without mRNA degradation, similar in principle to the method used in Wang *et al*. [34].

As Table 2 shows, the quality of fit (signal-to-noise ratio) on the training data was slightly worse (32.1 dB) in all non-SSM methods than in our SSM (32.4 dB). The predictive performance on the training data set was 90% of correct signs for all methods. The leave-out-last performance of 3) was worse (below 65% correct signs on the test set), while it was the same for 1) and 2) as in the LARS-based SSM, that is, 74% correct signs on the test set.

The non-SSM approaches that we considered in Table 2 would each give a unique solution, while our SSM would give slightly different solutions over consecutive runs, thus enabling a bootstrapping procedure. For this reason, our SSM offers a more principled way to deal with uncertainty and avoid over-fitting in microarray measurements than non-SSM methods. Second, our SSM is flexible because it enables adding unobserved variables as additional transcription factors.

## Conclusions

This systems biology study uses machine learning on time series of transcriptome data to generate testable hypotheses for the potential mechanisms underlying the NO_3_^- ^transduction signal. We demonstrate that a part of the NO_3_^- ^response (happening within minutes after NO_3_^- ^provision) has been missed by previous transcriptional approaches. This early response contains candidate transcription factors such as SPL9 that can modify the characteristics of NO_3_^- ^signal propagation in gene networks. Furthermore, we demonstrate that state-space modeling can infer putative regulatory networks on sparse datasets and thereby suggest hypotheses that will help to decipher poorly understood signaling pathways.

## Materials and methods

### Plant material and treatments

*Arabidopsis *plants, ecotype Columbia or transgenic plants (pSPL9:rSPL9) [46], were grown in sterile hydroponics as adapted from [10]. In detail, sterilized seeds were sown on Nitex 03-250/47 mesh (Sefar America, Bricarcliff Manor, NY, USA) supported by a plastic platform to allow roots to grow in hydroponics inside a sterile Phytatray (Sigma-Aldrich, St Louis, MO, USA). Hydroponic media consisted of 1X Murashige and Skoog basal medium containing no nitrogen or sucrose (custom-ordered, GibcoBRL, Gaithersburg, MD, USA) supplemented with 3 mM sucrose, 0.5 mM ammonium succinate, MES buffered at pH 5.7 (0.5 g.l^-1^). Plants were grown for 14 to 16 days in day/night cycles (16/8 h; 150 μmol photons m^-2^.s^-1^; Percival Scientific Inc., Perry, IA, USA) at 22°C. Twenty-four hours before the treatments, plants were transferred toward an equivalent fresh nitrogen-free medium in continuous light to avoid gene regulation induced by light. KNO_3 _was added to the media at 1 mM, or otherwise as stated in the figures. Control plants were mock-treated by adding the same concentration of KCl. The samples corresponding to time 0 were first harvested and then the treatment (KNO_3 _or KCl was applied by transferring the plants onto the refreshed nitrogen-free complemented media (with KNO_3 _and KCL). Then, the stopwatch was started. Roots were then harvested every 3 minutes and immediately frozen in liquid nitrogen. It takes an average of 20 to 30 seconds to harvest a sample. For growth experiments, plants were directly plated on petri dishes containing agar (1%) solidified media identical to the hydroponic one described above.

### RNA preparation and RT-QPCR analysis

Total RNA extraction was performed using Trizol™ Reagent according to the manufacturer's recommendations (Invitrogen, San Diego, CA, USA). Total RNA (1 to 2 μg) was digested by DNase I (Sigma). RNA was then reverse transcribed to one-strand cDNA using Thermo™ script RT (Invitrogen) according to the manufacturer's protocol. Gene expression was determined by RT-QPCR (LightCycler; Roche Diagnostics, Basel, Switzerland) using gene-specific primers (Additional file 7) and LightCycler FastStart DNA Master SYBR Green (Roche Diagnostics). Expression levels of tested genes were normalized to expression levels of the ACT2/8 and clathrin genes as described in [17].

### Microarray hybridization

cDNA was synthesized from 2 μg total RNA using T7- Oligo(dT) promoter primer and reagents recommended by Affymetrix (Santa Clara, CA, USA). Biotin-labeled cRNA was synthesized according to the manufacturer's protocol. Labeled cRNA (8 μg) was hybridized to *Arabidopsis *ATH1 Affymetrix gene chip for 16 h at 45°C. Washing, staining and scanning were performed as recommended by Affymetrix. Image analysis and normalization to a target median intensity of 150 was performed with the Affymetrix MAS v5.0 set at default values. We analyzed the reproducibility of replicates using the correlation coefficient and visual inspection of scatter plots of pairs of replicates.

### Modeling of gene expression patterns: clustering methods

The microarray data reported in this paper have been deposited in the Gene Expression Omnibus (GEO) database [GEO:GSE20044]. Data manipulations were performed in R [51]. All filtering steps were carried out by controlling the FDR. The data set, corresponding to 26 ATH1 chips times 22,810 probes, were analyzed in two successive steps in order to increase the stringency and precision of the analysis. The intent of the first step was to narrow the focus to genes regulated by nitrate over the entire data set or in interaction with time. Thus, we ran an ANOVA (*aov() *function) over the data set where the signal of a probe i is Pi ~ μ + αN + βT + γT*N + ε, where N is the effect of the nitrate treatment, T is the effect of time, and T*N is the effect of their interaction, μ is the mean signal over the data set, and ε the unexplained variance. We sorted out probes having a significant call (*P*-value < 0.01; corresponding to 10% < FDR < 15%), for the effect of N and T*N. This first pass identified 2,159 probes corresponding to nitrogen-regulated genes. On this narrowed data set (26 ATH1 chips times 2,159 probes), we modeled the gene expression using a linear modeling approach (*lm()*) in order to determine for each gene the particular time point that showed the most marked effect of nitrate. Thus, the expression of a gene (signal of a probe) was modeled such as Pi = α_0 _+ α_1_T + α_2_N + α_3_T*N + ε, where α_0 _represents the signal at 'time 0' (before the treatment). This modeling approach forces the model to take 'time 0' as a baseline for the possible effect of NO_3_^- ^or KCl, allowing us to eventually discriminate between the KNO_3 _and KCl effects. Thus, we called a probe regulated if it has a positive call (FDR < 5%) for interaction with nitrate at a particular time point.

The clustering shown in Figures 1 and 2 was performed through MeV software [52]. The number of clusters was determined using the figure of merit (FOM) method [53], followed by a clustering using Pearson correlation as the distance, and the average as method of aggregation. Additional file 8 provides the KNO_3_/KCl ratio along the kinetic for the entire 550 regulated genes.

### Randomization test

Data manipulations were performed in R [51]. We set up a test to monitor whether the overlap between two gene lists is higher than expected by chance. The test consists of randomly selecting 10,000 gene lists of *Arabidopsis thaliana *Gene Index (AGI) numbers, out of the genome, having the same size as the observed lists. Thus, we counted the number (n) of times that the intersection size of the random lists is equal or higher than the intersection observed for the two tested gene lists. A *P*-value is thus generated equal to n/10,000. For the generation of the matrix in Figure 3, the randomization test was limited to 1,000 gene lists in order to speed up the analysis.

### State-space model

Our SSM involves noisy but observed gene expression data **y**(*t*) (black circles in Figure 4a), as well as idealized but unobserved ('latent') gene expression values **z**(*t*) (red circles in Figure 4a). As shown in Figure 4a, the relationship between consecutive latent variables **z**(*t*_*k*_) and **z**(*t*_*k*__+1_) is a Markov chain: each latent gene's expression value at time *t*_*k*__+1 _is assumed to depend only on the state of potentially all the latent gene expressions at the previous time point *t*. For each gene *i*, this relationship stems from the kinetic ODE involving the rate of mRNA change (with a kinetic time constant *τ*), mRNA degradation, and a linear function *fi *of transcription factor concentrations for that specific gene. So-called 'Brownian motion' dynamics correspond to kinetic dynamics without the mRNA degradation term. In linearized (discretized) form, the overall dynamical model *f *can be represented by an *n × m *matrix **F **where *n *is the total number of genes and *m *the number of transcription factors (*m*<*n*, and transcription factors are given indexes from 1 to *m*), plus a bias term **b **and a Gaussian error term with zero mean and fixed covariance:

$$\begin{array}{c} \tau \frac{\text{d}z_{i}(t)}{\text{d}t}+z_{i}(t)=f_{i}(\text{z}(t))+\eta_{i}(t)\\ \frac{\tau }{t_{k+1}-t_{k}}(z_{i}(t_{k+1})-z_{i}(t_{k}))+z_{i}(t_{k})=\sum_{j=1}^{N_{i}}F_{i,j}z_{j}(t_{k})+b_{i,0}+\eta_{i}(t_{k}) \end{array}$$

The observation model *h *is essentially an *n × n *identity matrix with a Gaussian error term:

$$\begin{array}{c} y_{i}(t)=h(z_{i}(t))+\epsilon_{i}(t)\\ y_{i}(t_{k})=z_{i}(t_{k})+\epsilon_{i}(t_{k}) \end{array}$$

An iterative procedure tries to learn the dynamical relationship between latent gene expression variables **z**(*t*) while maintaining the latent variables **z**(*t*) as close as possible to the observed Affimetrix measures **y**(*t*). The algorithm consists of a) minimizing the sum of quadratic errors of the dynamical and the observation models with respect to the latent variables **Z **by using gradient descent on the latent variables [25] (this is the inference step), and b) minimizing the sum of quadratic errors of the dynamical model using conjugate gradient, LARS [42] or Elastic Nets [43] optimization on the parameters of **F **(this is the learning step). On a sequence **Y **of *T *microarray measurements (including replicate sequences) over *n *genes, corresponding latent variables **Z**, under a dynamic model parameterized by transcription factor-gene influence matrix **F **and bias term **b**, and for a given hyperparameter *γ *(which controls the weight of the dynamical and observation errors), the total error term for the inference step is:

$$e_{\text{γ}}(\text{Y},\text{Z};\text{F},\text{b})=\sum_{i=1}^{n}\sum_{k=1}^{T}\left(\text{γ}||\eta_{i}(t_{k})|{|}_{2}^{2}+||\epsilon_{i}(t_{k})|{|}_{2}^{2}\right)$$

The solution of a non-SSM can be recovered by setting *γ *= 0, which amounts to having exactly **Y **= **Z**.

During the learning step, sparse gene regulation networks are obtained by penalizing dense solutions using L1-norm regularization, which amounts to adding a *λ*-weighted penalty to the dynamical error term, as in the LASSO initially described by Tibshirani [44]. Employing regularization on parameters of **F **also helps to avoid local optima in the solutions.

The learning algorithm is run for 100 consecutive epochs over all the replicate sequences (four replicate sequences here). In order to retain the optimal set of parameters of *f*, one selects the epoch where the dynamic error on the training dataset is minimal. The cross-validation data are unseen during the optimization procedure. One run of the learning procedure yields a matrix **F **of signed (positive (excitatory) or negative (inhibitory)) interactions between transcription factors and genes. Each element F_i,j _represents the action of the j-th transcription factor on the i-th gene.

### Selection of gene regulation network by bootstrapping

The algorithm described above for learning SSMs starts with random initial values for both the dynamical model (in other words, matrix **F**) and for the latent variables **Z**. We repeat the whole procedure 20 times in order to perform the following bootstrapping evaluation. Each run *k *of the algorithm might converge to a slightly different solution F^*(k)^. We then take the average transcription factor-gene interaction weights obtained from all solutions F^*(k) ^and call it F*. Table 1 reports comparative results on the average solutions.

In parallel, we also generate 1,000 random permutations P^*(k,1)^, P^*(k,2)^, ..., P^*(k,1000) ^of each matrix F*^(k)^, and then compute 1,000 average matrices P^*(1)^, P^*(2)^, ..., P^*(1000) ^of those 'scrambled' matrices (we take the averages over the 20 runs). We compare each average element **F**_i,j_* to the empirical distribution of the 1,000 permuted averages and thus obtain an empirical *P*-value. The final genetic regulation network consists of elements **F**_i,j_* that have a *P*-value < 0.001.

### Software package

We provide online [54] our own SSM software, which is based on publication [25] and implements the algorithm described above. The software runs under Matlab (The Mathworks Inc., Natick, MA, USA) and requires the LARS library, designed by Karl Skoglund (IMM, DTU), which is available as part of the BoLASSO Matlab package.

## Abbreviations

FDR: false discovery rate; LARS: least-angle regression and shrinkage; LASSO: least absolute shrinkage and selection operator; ODE: ordinary differential equation; RT-QPCR: real time quantitative PCR; SSM: state space model.

## Authors' contributions

GK, DS, and GC designed the study. GK carried out the experiments. GK performed transcriptome data analysis. GK and DS developed the randomization test to infer signal convergence. PM, DS, and YL developed the machine learning approach and the SSM algorithm. GK, PM, DS, and GC wrote the paper.

## Supplementary Material

Additional file 1 **Description of significantly regulated genes and their cluster assignment**. Click here for file

Additional file 2 **High-resolution kinetics of response of genes to NO**_**3**_^- ^**treatment**. Levels of mRNA for nitrogen-responsive genes in *Arabidopsis *roots in response to NO_3_^- ^treatment. Fourteen-day-old plants grown in the presence of ammonium succinate were treated with 1 mM KNO_3 _or KCL (as a mock treatment). Plants were collected at 0 minutes (before treatment) and 3, 6, 9, 12, 15, and 20 minutes after treatment. Transcripts were measured in RNA from roots using RT-QPCR and Affy ATH1 chips and normalized to two housekeeping genes (see Materials and methods). The data represent the mean ± standard error of three and two biological replicates for QPCR and Affymetrix measurements, respectively. Correlations between QPCR- and Affy-detected fold change results are provided in the third column. Click here for file

Additional file 3 **Gene Ontology functions over-represented in NO**_**3**_^-^**-regulated gene lists**. Click here for file

Additional file 4 **Gene Ontology functions over-represented in NO**_**3**_^- ^**clusters**. Click here for file

Additional file 5 **SPL9 over-expression modifies NO**_**3**_^- ^**kinetic responses of transcription factors**. Transcription factor mRNA levels in roots of wild type and transgenic pSPL9:rSPL9 in response to NO_3_^- ^treatment. Fourteen-day-old plants grown in ammonium succinate were treated with 1 mM KNO_3 _versus KCl. Roots were collected at 0 minutes (before treatment) and 10, 20, 40, and 60 minutes after the treatment. Sentinel transcripts were measured in roots using RT-QPCR and normalized to two housekeeping genes (see Materials and methods). The data represent the mean ± standard error of three biological replicates (three independent experiments). Differences between the two genotypes are statistically significant at *P < 0.05, **P < 0.01, and ***P < 0.001 (*t*-test). Click here for file

Additional file 6 **NO**_**3**_^- ^**presence enhances pSPL9:rSPL9 phenotypes**. Wild type and pSPL9:rSPL9 have been plated on the same background media as used for kinetic experiments (see Materials and methods) and complemented with either 0.5 mM (NH_4_)_2_-succinate or 1 mM KNO_3_. Eight days after germination, different root traits were scored (n = 13 to 24 plants). ANOVA was used to evaluate the genotype effect (Geno: wild type versus pSPL9:rSPL9); the treatment effect (Treat: nitrate versus ammonium) and the interaction of these two factors (G × T). *P*-values are provided above each plot corresponding to different root parameters. ***P < 0.001; **P < 0.01; *P < 0.05. NO_3_^- ^presence significantly enhances transgene effect for lateral root number, total lateral root length, lateral root density, and lateral root length density. The average length of lateral roots is not significantly controlled by the pSPL9:rSPL9 transgene. Click here for file

Additional file 7 **QPCR primers used in this study**. Click here for file

Additional file 8 **KNO**_**3**_**/KCl gene expression ratio of significantly regulated genes (see also raw data [GEO:GSE20044])**. Click here for file
