# Supplementary material for: Predictive network modeling of the high-resolution dynamic plant transcriptome in response to nitrate
Source: Genome Biol. 2010 Dec 23;11(12):R123. doi: 10.1186/gb-2010-11-12-r123 (PMC3046483; doi:10.1186/gb-2010-11-12-r123)
Supplement: Additional file 1 — Description of significantly regulated genes and their cluster assignment. [file gb-2010-11-12-r123-S1.pdf]

AGI	annotation	Cluster
At5g67420	LBD37,LOB domain protein 37 / lateral organ boundaries domain protein 37 (LBD37)	1
At5g45340	CYP707A3,CYP707A3 (cytochrome P450, family 707, subfamily A, polypeptide 3); oxygen binding	1
At5g39870	unknown protein	1
At5g10210	unknown protein	1
At5g24160	squalene monooxygenase 1,2 / squalene epoxidase 1,2 (SQP1,2)	1
At1g24280	G6PD3,G6PD3 (GLUCOSE-6-PHOSPHATE DEHYDROGENASE 3); glucose-6-phosphate 1-dehydrogenase	1
At3g25790	myb family transcription factor	1
At5g09800	U-box domain-containing protein	1
At1g49000	unknown protein	1
At3g07350	unknown protein	1
At4g10310	HKT1,HKT1 (HIGH-AFFINITY K+ TRANSPORTER 1); sodium ion transporter	1
At1g13300	myb family transcription factor	1
At3g62930	glutaredoxin family protein	1
At3g49940	LBD38,LOB domain protein 38 / lateral organ boundaries domain protein 38 (LBD38)	1
At2g30040	MAPKKK14,MAPKKK14 (Mitogen-activated protein kinase kinase kinase 14); kinase	1
At4g37540	LBD39,LOB domain protein 39 / lateral organ boundaries domain protein 39 (LBD39)	1
At5g19120	pepsin A	1
At4g18350	NCED2,NCED2 (NINE-CIS-EPOXYCAROTENOID DIOXYGENASE 2)	2
At4g26390	pyruvate kinase, putative	2
At2g04730	pseudogene	2
At3g62410	CP12-2	2
At1g35290	thioesterase family protein	2
At1g18485	pentatricopeptide (PPR) repeat-containing protein	2
At2g16010	transposable element gene At2g16010	3
At1g70080	terpene synthase/cyclase family protein	3
At3g50750	brassinosteroid signalling positive regulator-related	3
At2g42050	transposable element gene At2g42050	3
At5g66350	SHI,SHI (SHORT INTERNODES); transcription factor	3
At4g25100	FSD1,FSD1 (FE SUPEROXIDE DISMUTASE 1); iron superoxide dismutase	3
At5g46330	FLS2,FLS2 (FLAGELLIN-SENSITIVE 2); ATP binding / kinase/ protein binding / protein serine/threonine kinase/ transmembrane receptor protein serine/threonine kinase	3
At3g29690	transferase-related	3
At3g30800	transposable element gene At3g30800	3
At5g09360	LAC14,LAC14 (laccase 14); copper ion binding / oxidoreductase	3
At2g29940	PDR3,ATPDR3,ATPDR3/PDR3 (PLEIOTROPIC DRUG RESISTANCE 3); ATPase, coupled to transmembrane movement of substances	3
At4g30090	EMB1353,EMB1353 (EMBRYO DEFECTIVE 1353)	3
At5g52170	homeobox-leucine zipper family protein / lipid-binding START domain-containing protein	4
At2g28690	unknown protein	4
At1g75590	auxin-responsive family protein	4
At3g28510	AAA-type ATPase family protein	4

At5g28620	protein kinase C-related	4
At5g65300	unknown protein	4
At5g44140	ATPHB7,ATPHB7 (PROHIBITIN 7)	4
At4g00416	MBD3,MBD3 (methyl-CpG-binding domain 3); DNA binding	4
At4g16447	unknown protein	5
At1g64380	AP2 domain-containing transcription factor, putative	5
At5g22890	zinc finger (C2H2 type) family protein	5
At1g32170	XTR4,XTR4 (XYLOGLUCAN ENDOTRANSGLYCOSYLASE 4); hydrolase, acting on glycosyl bonds	5
At1g21910	AP2 domain-containing transcription factor family protein	5
At2g47440	DNAJ heat shock N-terminal domain-containing protein	5
At1g29160	Dof-type zinc finger domain-containing protein	5
At1g78100	F-box family protein	5
At2g37430	zinc finger (C2H2 type) family protein (ZAT11)	5
At1g29180	DC1 domain-containing protein	5
At3g02140	TMAC2,TMAC2 (TWO OR MORE ABRES-CONTAINING GENE 2)	5
At1g22640	MYB3,MYB3 (myb domain protein 3); DNA binding / transcription factor	5
At5g11930	glutaredoxin family protein	5
At1g72240	unknown protein	5
At4g37240	unknown protein	5
At3g30180	CYP85A2,BR6OX2,BR6OX2/CYP85A2 (BRASSINOSTEROID-6-OXIDASE 2); monooxygenase/ oxygen binding	5
At3g25980	mitotic spindle checkpoint protein, putative (MAD2)	5
At1g74940	senescence-associated protein-related	5
At5g25280	serine-rich protein-related	5
At2g01150	RHA2B,RHA2B (RING-H2 FINGER PROTEIN 2B); protein binding / zinc ion binding	5
At2g02630	DC1 domain-containing protein	5
At1g15100	RHA2A,RHA2A (RING-H2 finger A2A); protein binding / zinc ion binding	5
At2g19460	unknown protein	5
At1g67340	zinc finger (MYND type) family protein / F-box family protein	5
At2g27570	sulfotransferase family protein	5
At5g58530	glutaredoxin family protein	5
At1g20070	unknown protein	5
At4g10390	protein kinase family protein	5
At2g37440	endonuclease/exonuclease/phosphatase family protein	5
At3g62630	unknown protein	5
At3g60490	AP2 domain-containing transcription factor TINY, putative	5
At5g24655	unknown protein	5
At5g47060	senescence-associated protein-related	5
At1g60670	unknown protein	5
At4g09900	hydrolase, alpha/beta fold family protein	5
At3g28200	peroxidase, putative	5
At2g45430	DNA-binding protein-related	5
At4g16680	RNA helicase, putative	6
At1g69440	AGO7,AGO7 (ARGONAUTE7)	6
At4g05610	transposable element gene At4g05610	6
At4g13240	ROPGEF9,ATROPGEF9,ATROPGEF9/ROPGEF9 (KINASE PARTNER PROTEIN-LIKE); Rho guanyl-nucleotide exchange factor	6
At5g19890	peroxidase, putative	6

At5g56070	unknown protein	6
At2g44440	emsy N terminus domain-containing protein / ENT domain-containing protein	7
At5g37610	voltage-gated ion-selective channel	7
At1g05920	DNA binding	7
At1g17810	BETA-TIP,BETA-TIP (BETA-TONOPLAST INTRINSIC PROTEIN); water channel	7
AtMg01040	ORF107F,unknown protein	7
At3g07040	RPM1,RPM1 (RESISTANCE TO P. SYRINGAE PV MACULICOLA 1)	7
At1g61410	tolA protein-related	7
At1g51990	O-methyltransferase family 2 protein	7
At4g07586	transposable element gene At4g07586	7
At2g34330	unknown protein	7
At4g23320	protein kinase family protein	7
At2g06220	transposable element gene At2g06220	7
At2g45930	unknown protein	7
At4g04600	transposable element gene At4g04600	7
At2g46190	unknown protein	7
At5g55750	hydroxyproline-rich glycoprotein family protein	7
At4g36770	UDP-glucoronosyl/UDP-glucosyl transferase family protein	7
At3g21340	leucine-rich repeat protein kinase, putative	8
At5g23020	MAM-L,MAM-L (METHYLTHIOALKYMALATE SYNTHASE-LIKE); 2-isopropylmalate synthase	8
At5g23010	MAM1,MAM1 (2-isopropylmalate synthase 3); 2-isopropylmalate synthase	8
At5g18670	BMY3,BMY3 (beta-amylase 3); beta-amylase	8
At1g19030	transposable element gene At1g19030	8
At1g20390	transposable element gene At1g20390	8
At2g07740	transposable element gene At2g07740	8
At2g24660	transposable element gene At2g24660	8
At5g35820	transposable element gene At5g35820	8
At1g18100	E12A11,E12A11; phosphatidylethanolamine binding	8
At1g10810	aldo/keto reductase family protein	8
At3g25490	wall-associated kinase, putative	8
At1g15730	PRLI-interacting factor L, putative	8
At4g13880	leucine-rich repeat family protein	8
At5g56870	BGAL4,beta-galactosidase, putative / lactase, putative	8
At1g32920	unknown protein	9
At1g49230	zinc finger (C3HC4-type RING finger) family protein	9
At4g25835	AAA-type ATPase family protein	9
At5g59080	unknown protein	9
At5g67390	unknown protein	9
At4g05390	ATRFNR1,ATRFNR1 (ROOT FNR 1); oxidoreductase	9
At4g37610	BT5,BT5 (BTB and TAZ domain protein 5); protein binding / transcription regulator	9
At3g63110	ATIPT3,ATIPT3 (Arabidopsis thaliana isopentenyltransferase 3); transferase, transferring alkyl or aryl (other than methyl) groups	9
At5g15830	ATBZIP3,bZIP transcription factor family protein	9
At4g36010	pathogenesis-related thaumatin family protein	9
At2g38510	MATE efflux protein-related	9
At3g04980	DNAJ heat shock N-terminal domain-containing protein	9
At1g68880	ATBZIP,bZIP transcription factor family protein	9

At1g07150	MAPKKK13,MAPKKK13 (Mitogen-activated protein kinase kinase kinase 13); kinase	9
At1g68670	myb family transcription factor	9
At2g48080	oxidoreductase, 2OG-Fe(II) oxygenase family protein	9
At5g04840	bZIP protein	9
At2g27830	unknown protein	9
At2g15620	NIR1,NIR1 (NITRITE REDUCTASE); ferredoxin-nitrate reductase	9
At1g25550	myb family transcription factor	9
At5g35870	unknown protein	9
At4g34760	auxin-responsive family protein	9
At3g04530	PPCK2,PPCK2 (PHOSPHOENOLPYRUVATE CARBOXYLASE KINASE 2); kinase	9
At1g78050	phosphoglycerate/bisphosphoglycerate mutase family protein	9
At5g40850	UPM1,UPM1 (UROPHORPHYRIN METHYLASE 1); uroporphyrin-III C-methyltransferase	9
At2g25450	2-oxoglutarate-dependent dioxygenase, putative	10
At4g04840	methionine sulfoxide reductase domain-containing protein / SeIR domain-containing protein	10
At2g27510	ATFD3,ATFD3 (FERREDOXIN 3); electron carrier	10
At1g15380	lactoylglutathione lyase,lactoylglutathione lyase family protein / glyoxalase I family protein	10
AtCg00300	YCF9,unknown protein	10
At3g05950	germin-like protein, putative	10
At4g36350	PAP25,ATPAP25,ATPAP25/PAP25 (purple acid phosphatase 25); acid phosphatase/ protein serine/threonine phosphatase	10
At1g55120	ATFRUCT5,ATFRUCT5 (BETA-FRUCTOFURANOSIDASE 5); hydrolase, hydrolyzing O-glycosyl compounds / levanase,unknown protein	10
At1g13420	sulfotransferase family protein	10
At5g22570	WRKY38,WRKY38 (WRKY DNA-binding protein 38); transcription factor	10
At4g30450	glycine-rich protein	10
At5g04950	nicotianamine synthase, putative	10
At5g59090	ATSBT4.12,subtilase,subtilase family protein	10
At3g16150	L-asparaginase, putative / L-asparagine amidohydrolase, putative	10
At4g30460	glycine-rich protein	10
At1g70410	carbonic anhydrase, putative / carbonate dehydratase, putative	10
At2g17820	ATHK1,ATHK1 (HISTIDINE KINASE 1)	10
At1g08090	ATNRT2:1,ATNRT2:1 (Arabidopsis thaliana high affinity nitrate transporter 2.1); nitrate transporter	10
At1g54120	unknown protein	10
At5g57655	xylose isomerase family protein	10
At1g35570	transposable element gene At1g35570	10
At3g61430	PIP1A,PIP1A (plasma membrane intrinsic protein 1;1),PIP1A (plasma membrane intrinsic protein 1;1); water channel	10
At1g20160	ATSBT5.2,subtilase,subtilase family protein	10
At4g37180	myb family transcription factor	11
At1g15550	GA4,GA4 (GA REQUIRING 4); gibberellin 3-beta-dioxygenase	11
At4g36040	DNAJ heat shock N-terminal domain-containing protein (J11)	11
At4g18340	glycosyl hydrolase family 17 protein	11
At3g13730	CYP90D1,CYP90D1 (CYTOCHROME P450, FAMILY 90, SUBFAMILY D, POLYPEPTIDE 1); oxidoreductase, acting on paired donors, with incorporation or reduction of molecular oxygen, NADH or NADPH as one	11

	donor, and incorporation of one atom of oxygen / oxygen binding	
At5g54170	unknown protein	11
At4g03510	RMA1,RMA1 (Ring finger protein with Membrane Anchor 1); protein binding / ubiquitin-protein ligase/ zinc ion binding	11
At1g49160	WNK7,WNK7 (Arabidopsis WNK kinase 7); kinase	11
At3g46130	MYB111,MYB111 (myb domain protein 111),MYB111 (myb domain protein 111); DNA binding,MYB111 (myb domain protein 111); DNA binding / transcription factor	11
At1g16420	ATMC8,latex-abundant protein, putative (AMC8) / caspase family protein	11
At4g36540	BEE2,BEE2 (BR ENHANCED EXPRESSION 2); DNA binding / transcription factor	11
At1g14260	zinc finger (C3HC4-type RING finger) family protein	11
At3g18560	unknown protein	11
At5g14760	AO,AO (L-ASPARTATE OXIDASE); L-aspartate oxidase	11
At1g68500	unknown protein	11
At1g68360	zinc finger protein-related	11
At4g02380	SAG21,SAG21 (SENESCENCE-ASSOCIATED GENE 21)	11
At1g14550	anionic peroxidase, putative	12
At1g73260	trypsin and protease inhibitor family protein / Kunitz family protein	12
At5g17490	RGL3,RGL3 (RGA-LIKE 3); transcription factor	12
At1g26380	FAD-binding domain-containing protein	12
At4g13440	calcium-binding EF hand family protein	12
At1g14540	anionic peroxidase, putative	12
At1g27880	ATP-dependent DNA helicase, putative	12
At5g40990	GLIP1,GLIP1 (GDSL LIPASE1); carboxylic ester hydrolase	12
At4g39950	CYP79B2,CYP79B2 (cytochrome P450, family 79, subfamily B, polypeptide 2); oxygen binding	12
At3g49740	pentatricopeptide (PPR) repeat-containing protein	12
At3g49780	ATPSK4,ATPSK4 (PHYTOSULFOKINE 4 PRECURSOR); growth factor	12
At4g39940	AKN2,AKN2 (APS-KINASE 2); ATP binding / kinase/ transferase, transferring phosphorus-containing groups	12
At5g64120	peroxidase, putative	12
At2g43000	ANAC042,ANAC042 (Arabidopsis NAC domain containing protein 42); transcription factor	12
At5g39580	peroxidase,peroxidase, putative	12
At4g36880	CP1,cysteine proteinase, putative	12
At5g36870	ATGSL09,ATGSL09 (GLUCAN SYNTHASE-LIKE 9); 1,3-beta-glucan synthase	12
At1g65860	flavin-containing monooxygenase family protein / FMO family protein	12
At2g39370	unknown protein	13
At5g62720	integral membrane HPP family protein	13
At4g18510	CLE2,CLE2 (CLAVATA3/ESR-RELATED); receptor binding	13
At5g19260	unknown protein	13
At2g40750	WRKY54,WRKY54 (WRKY DNA-binding protein 54); transcription factor	13
At4g09620	unknown protein	13
At4g39780	AP2 domain-containing transcription factor, putative	13
At1g77760	NIA1,NIA1 (NITRATE REDUCTASE 1)	13
At4g40070	protein binding / zinc ion binding	13
At3g19030	unknown protein	13
At3g50900	unknown protein	13
At5g41670	6-phosphogluconate dehydrogenase family protein	13

At1g66140	ZFP4,ZFP4 (ZINC FINGER PROTEIN 4); nucleic acid binding / transcription factor/ zinc ion binding	13
At2g22500	mitochondrial substrate carrier family protein	13
At5g28610	unknown protein	13
At1g64190	6-phosphogluconate dehydrogenase family protein	13
At1g12740	CYP87A2,CYP87A2 (cytochrome P450, family 87, subfamily A, polypeptide 2); oxygen binding	13
At3g17510	CIPK1,CIPK1 (CBL-INTERACTING PROTEIN KINASE 1); kinase	13
At5g15330	SPX (SYG1/Pho81/XPR1) domain-containing protein	13
At1g67910	unknown protein	13
At2g24550	unknown protein	13
At5g13110	G6PD2,G6PD2 (GLUCOSE-6-PHOSPHATE DEHYDROGENASE 2); glucose-6-phosphate 1-dehydrogenase	13
At1g67920	unknown protein	13
At1g30510	ATRFNR2,ATRFNR2 (ROOT FNR 2); oxidoreductase	13
At3g57450	unknown protein	13
AtCg00420	NDHJ,unknown protein	13
At3g53150	UGT73D1,UGT73D1 (UDP-glucosyl transferase 73D1); UDP-glycosyltransferase	13
At1g08100	ATNRT2.2,ATNRT2.2 (Arabidopsis thaliana high-affinity nitrate transporter 2.2); nitrate transporter	13
At5g03380	heavy-metal-associated domain-containing protein,metal ion binding	13
At5g65210	TGA1,DNA binding / calmodulin binding / transcription factor,bZIP family transcription factor (TGA1)	13
At3g47980	integral membrane HPP family protein	13
At3g15760	unknown protein	13
At4g34750	auxin-responsive protein, putative / small auxin up RNA (SAUR_E)	13
At4g02920	unknown protein	13
At1g77400	unknown protein	13
At5g01740	unknown protein	13
At1g78090	ATTPPB,ATTPPB (TREHALOSE-6-PHOSPHATE PHOSPHATASE)	13
At4g38340	RWP-RK domain-containing protein	13
At4g32950	protein phosphatase 2C, putative / PP2C, putative	13
At1g69760	unknown protein	13
At1g79110	protein binding / zinc ion binding	13
At5g42830	transferase family protein	13
At4g18880	AT-HSFA4A,AT-HSFA4A (Arabidopsis thaliana heat shock transcription factor A4A); DNA binding / transcription factor	14
At1g21000	unknown protein,zinc-binding family protein	14
At1g17060	CYP72C1,CYP72C1 (cytochrome P450, family 72, subfamily C, polypeptide 1); oxygen binding	14
At2g31955	CNX2,CNX2 (COFACTOR OF NITRATE REDUCTASE AND XANTHINE DEHYDROGENASE 2); catalytic	14
At3g61590	HWS,HS,F-box family protein	14
At5g62900	unknown protein	14
At4g21350	PUB8,B80,B80; binding / ubiquitin-protein ligase	14
At1g12110	NRT1.1,NRT1.1 (nitrate transporter 1.1); transporter	14
At4g17550	transporter-related	14
At2g28890	PLL4,PLL4 (POLTERGEIST LIKE 4); protein phosphatase type 2C	14
At5g20885	zinc finger (C3HC4-type RING finger) family protein	14

At5g51050	mitochondrial substrate carrier family protein	14
At4g24020	RWP-RK domain-containing protein	14
At1g43710	EMB1075,EMB1075 (EMBRYO DEFECTIVE 1075); carboxy-lyase	14
At5g40470	unknown protein	14
At3g25780	AOC3,AOC3 (ALLENE OXIDE CYCLASE 3)	14
At4g27900	unknown protein	14
At1g49860	ATGSTF14,ATGSTF14 (Arabidopsis thaliana Glutathione S-transferase (class phi) 14); glutathione transferase	14
At4g37550	formamidase,formamidase, putative / formamide amidohydrolase, putative	14
At1g63940	monodehydroascorbate reductase, putative	14
At1g12820	AFB3,AFB3 (AUXIN SIGNALING F-BOX 3); auxin binding / ubiquitin-protein ligase	14
At3g18710	U-box domain-containing protein	14
At3g55980	zinc finger (CCCH-type) family protein	14
At3g06590	transcription factor,unknown protein	14
At5g51830	pfkB-type carbohydrate kinase family protein	14
At3g52950	CBS domain-containing protein / octicosapeptide/Phox/Bemp1 (PB1) domain-containing protein	14
At4g31730	GDU1,GDU1 (GLUTAMINE DUMPER 1)	14
At3g23030	IAA2,IAA2 (indoleacetic acid-induced protein 2); transcription factor	14
At3g11580	DNA-binding protein, putative	14
At1g71980	protease-associated zinc finger (C3HC4-type RING finger) family protein	14
At4g34000	DPBF5,ABF3,ABF3/DPBF5 (ABSCISIC ACID RESPONSIVE ELEMENTS-BINDING FACTOR 3); DNA binding / protein binding / transcription factor/ transcriptional activator	14
At3g28690	kinase,protein kinase, putative	14
At1g70780	unknown protein	14
At5g64550	loricrin-related	14
At1g62180	APR2,APR2 (5'ADENYLYLPHOSPHOSULFATE REDUCTASE 2)	14
At5g58900	myb family transcription factor	14
At5g65140	trehalose-6-phosphate phosphatase, putative,unknown protein	14
At5g12860	DIT1,DIT1 (DICARBOXYLATE TRANSPORTER 1); oxoglutarate:malate antiporter,unknown protein	14
At3g52360	unknown protein	14
At4g31910	transferase family protein	14
At1g16170	unknown protein	14
At5g08010	unknown protein	14
At1g64140	unknown protein	14
At3g26090	RGS1,RGS1 (REGULATOR OF G-PROTEIN SIGNALING 1); signal transducer	14
At5g04250	OTU-like cysteine protease family protein,unknown protein	14
At4g27180	ATK2,ATK2 (ARABIDOPSIS THALIANA KINESIN 2); microtubule motor	15
At4g13650	pentatricopeptide (PPR) repeat-containing protein	15
At3g60690	auxin-responsive family protein	15
At1g69040	ACR4,ACR4 (ACT REPEAT 4); amino acid binding	15
At4g24670	alliinase family protein	15
At3g58760	ankyrin protein kinase, putative	15
At1g72200	zinc finger (C3HC4-type RING finger) family protein	15
At3g60750	transketolase, putative	15
At1g02900	RALFL1,RALFL1 (RALF-LIKE 1)	15
At2g28250	kinase,protein kinase family protein	15

At5g24890	unknown protein	15
At5g62530	ALDH12A1,ALDH12A1 (Aldehyde dehydrogenase 12A1); 1-pyrroline-5-carboxylate dehydrogenase/ 3-chloroallyl aldehyde dehydrogenase	15
At1g74660	MIF1,MIF1 (MINI ZINC FINGER 1); DNA binding / transcription factor	15
At3g27210	unknown protein	15
At2g31940	oxidoreductase/ transition metal ion binding	15
At2g24790	COL3,COL3 (CONSTANS-LIKE 3); protein binding / transcription factor/ zinc ion binding,COL3 (CONSTANS-LIKE 3); zinc ion binding	15
At5g01340	mitochondrial substrate carrier family protein	15
At5g47100	CBL9,CBL9 (Calcineurin B-like protein 9); calcium ion binding	15
At5g35630	GS2,GS2 (GLUTAMINE SYNTHETASE 2),GS2 (GLUTAMINE SYNTHETASE 2); glutamate-ammonia ligase	15
At4g26130	unknown protein	15
At5g50200	WR3,WR3 (WOUND-RESPONSIVE 3),WR3 (WOUND-RESPONSIVE 3); nitrate transporter	15
At3g14940	ATPPC3,ATPPC3 (PHOSPHOENOLPYRUVATE CARBOXYLASE 3); phosphoenolpyruvate carboxylase	15
At1g56230	unknown protein	15
At1g22160	senescence-associated protein-related	15
At2g17150	RWP-RK domain-containing protein	15
At5g39590	unknown protein	15
At3g49760	ATBZIP5,bZIP transcription factor family protein	15
At2g42070	ATNUDT23,ATNUDT23 (Arabidopsis thaliana Nudix hydrolase homolog 23); hydrolase	15
At4g30190	AHA2,AHA2 (Arabidopsis H(+)-ATPase 2); ATPase	15
At3g29760	NLI interacting factor (NIF) family protein	15
At5g58690	phosphoinositide-specific phospholipase C family protein	15
At5g19010	MPK16,MPK16 (mitogen-activated protein kinase 16); MAP kinase	15
At5g53460	GLT1,GLT1 (NADH-dependent glutamate synthase 1 gene)	15
At1g64370	unknown protein	15
At2g38170	CAX1,CAX1 (CATION EXCHANGER 1); calcium:hydrogen antiporter	15
At4g23980	ARF9,ARF9 (AUXIN RESPONSE FACTOR 9),ARF9 (AUXIN RESPONSE FACTOR 9); transcription factor	15
At5g10820	integral membrane transporter family protein	15
At5g17880	CSA1,CSA1 (CONSTITUTIVE SHADE-AVOIDANCE1); ATP binding / protein binding / transmembrane receptor	15
At4g35260	IDH1,IDH1 (ISOCITRATE DEHYDROGENASE 1); isocitrate dehydrogenase (NAD+)	15
AtCg00510	PSAI,unknown protein	15
At3g07310	unknown protein	15
At5g06570	unknown protein	15
At5g58700	phosphoinositide phospholipase C,phosphoinositide-specific phospholipase C family protein	15
AtCg00180	RPOC1,unknown protein	15
At3g28050	nodulin MtN21 family protein	15
At2g18160	GBF5,ATBZIP2,GBF5 (G-box binding factor 5); DNA binding / transcription factor	15
At5g60770	ATNRT2.4,ATNRT2.4 (Arabidopsis thaliana high affinity nitrate transporter 2.4); nitrate transporter	15
At1g61930	unknown protein	15
At5g20540	BRX-LIKE4,ATBRXL4,ATBRXL4/BRX-LIKE4 (BREVIS RADIX-LIKE 4)	15

At3g47520	MDH,MDH (malate dehydrogenase); malate dehydrogenase	15
At5g13420	transaldolase, putative	15
At1g50110	branched-chain amino acid aminotransferase 6 / branched-chain amino acid transaminase 6 (BCAT6)	15
At5g10030	TGA4,TGA4 (TGACG MOTIF-BINDING FACTOR 4); DNA binding / calmodulin binding / transcription factor	15
At1g19050	ARR7,ARR7 (RESPONSE REGULATOR 7); transcription regulator/ two-component response regulator	15
At4g24620	PGI1,PGI1 (CHLOROPLASTIC PHOSPHOGLUCOSE ISOMERASE)	15
At1g73000	unknown protein	15
At1g22170	phosphoglycerate/bisphosphoglycerate mutase family protein	15
At5g59730	ATEXO70H7,ATEXO70H7 (EXOCYST SUBUNIT EXO70 FAMILY PROTEIN H7); protein binding	15
At1g13250	GATL3,GATL3 (Galacturonosyltransferase-like 3); polygalacturonate 4-alpha-galacturonosyltransferase/ transferase, transferring glycosyl groups / transferase, transferring hexosyl groups	15
At2g17060	disease resistance protein (TIR-NBS-LRR class), putative	15
At5g61420	MYB28,MYB28 (myb domain protein 28); DNA binding / transcription factor	15
At2g46870	NGA1,NGA1 (NGATHA1); transcription factor	16
At3g44960	unknown protein	16
At1g70985	hydroxyproline-rich glycoprotein family protein	16
At5g14980	esterase/lipase/thioesterase family protein	16
At1g80380	phosphoribulokinase/uridine kinase-related	16
At5g54130	unknown protein	16
At4g20000	VQ motif-containing protein	16
At4g16780	ATHB-2,ATHB-2 (Homeobox-leucine zipper protein HAT4); DNA binding / transcription factor	16
At4g18250	receptor serine/threonine kinase, putative	16
AtCg00780	RPL14,unknown protein	16
At2g36320	zinc finger (AN1-like) family protein	17
At1g27900	RNA helicase, putative	17
At2g42660	myb family transcription factor	17
At2g30860	ATGSTF9,ATGSTF9 (Arabidopsis thaliana Glutathione S-transferase (class phi) 9),ATGSTF9 (Arabidopsis thaliana Glutathione S-transferase (class phi) 9); glutathione transferase	17
At1g80460	NHO1,NHO1 (NONHOST RESISTANCE TO P. S. PHASEOLICOLA 1); carbohydrate kinase	17
At1g69450	unknown protein	17
At2g28190	CSD2,CSD2 (COPPER/ZINC SUPEROXIDE DISMUTASE 2); copper, zinc superoxide dismutase	17
At2g25980	jacalin lectin family protein	17
At1g62660	beta-fructosidase (BFRUCT3) / beta-fructofuranosidase / invertase, vacuolar	17
At2g18030	peptide methionine sulfoxide reductase family protein	17
At2g33390	unknown protein	17
At1g52760	esterase/lipase/thioesterase family protein	17
At1g64590	short-chain dehydrogenase/reductase (SDR) family protein	17
At5g36940	CAT3,CAT3 (CATIONIC AMINO ACID TRANSPORTER 3); cationic amino acid transporter	17
At1g22400	UGT85A1,ATUGT85A1,UGT85A1 (UDP-glucosyl transferase 85A1); UDP-glycosyltransferase/ transferase, transferring glycosyl groups / transferase, transferring hexosyl groups	17

At1g21920	MORN (Membrane Occupation and Recognition Nexus) repeat-containing protein /phosphatidylinositol-4-phosphate 5-kinase-related	17
At3g14050	RSH2,RSH2 (RELA-SPOT HOMOLOG); catalytic	17
At4g01450	nodulin MtN21 family protein	17
At3g57790	glycoside hydrolase family 28 protein / polygalacturonase (pectinase) family protein	17
At1g09575	unknown protein	17
At1g23760	JP630,JP630; polygalacturonase	17
At1g50350	unknown protein	17
At5g35980	kinase,protein kinase family protein	17
At3g08650	metal transporter family protein	17
At3g20740	FIE,FIE (FERTILIZATION-INDEPENDENT ENDOSPERM 1); nucleotide binding / transcription factor	17
At5g36160	aminotransferase-related	17
At1g09570	PHYA,PHYA (PHYTOCHROME A); G-protein coupled photoreceptor/ signal transducer,unknown protein	17
At5g37055	ATSWC6,zinc finger (HIT type) family protein	17
At1g63690	protease-associated (PA) domain-containing protein	17
At3g09260	PYK10,PYK10 (phosphate starvation-response 3.1); hydrolase, hydrolyzing O-glycosyl compounds	17
At3g60070	lactose permease-related	17
At3g50910	unknown protein	17
At5g66800	unknown protein	17
At3g23080	unknown protein	17
At4g25880	APUM6,APUM6 (ARABIDOPSIS PUMILIO 6); RNA binding	17
At1g19850	MP,MP (MONOPTEROS); transcription factor	17
At5g48590	unknown protein	17
At3g19190	unknown protein	17
At3g10740	ASD1,ASD1 (ALPHA-L-ARABINOFURANOSIDASE); hydrolase, acting on glycosyl bonds	17
At1g12520	CCS1,CCS1 (copper chaperone for superoxide dismutase 1); superoxide dismutase copper chaperone	17
At4g15270	glucosyltransferase-related	17
At2g24330	unknown protein	17
At2g35270	DNA-binding protein-related	17
At1g32850	ubiquitin carboxyl-terminal hydrolase family protein	17
At1g61360	S-locus lectin protein kinase family protein	17
At3g06380	ATTLP9,ATTLP9/AtTLP9 (TUBBY LIKE PROTEIN 9, TUBBY-LIKE PROTEIN 9); phosphoric diester hydrolase/ protein binding / transcription factor	17
At1g24440	protein binding / zinc ion binding	17
At3g48990	AMP-dependent synthetase and ligase family protein	17
At3g19010	oxidoreductase, 2OG-Fe(II) oxygenase family protein	17
At2g40890	CYP98A3,CYP98A3 (cytochrome P450, family 98, subfamily A, polypeptide 3); p-coumarate 3-hydroxylase	17
At2g28910	CXIP4,CXIP4 (CAX INTERACTING PROTEIN 4); nucleic acid binding / zinc ion binding	17
At3g16460	jacalin lectin family protein	17
At1g08750	GPI-anchor transamidase, putative	17
AtCg00080	PSBI,unknown protein	17
At2g19110	HMA4,HMA4 (Heavy metal ATPase 4); cadmium-transporting ATPase	17

At4g13430	aconitase family protein / aconitate hydratase family protein	17
At5g11510	MYB3R-4,MYB3R-4 (C-MYB-LIKE TRANSCRIPTION FACTOR 3R-4, myb domain protein 3R-4); DNA binding / transcription factor	17
At4g04040	MEE51,MEE51 (maternal effect embryo arrest 51); diphosphate-fructose-6-phosphate 1-phosphotransferase	17
At1g55310	SR33,SR33 (SC35-like splicing factor 33); RNA binding	17
At5g06800	myb family transcription factor	17
At3g47050	glycosyl hydrolase family 3 protein	17
At4g37150	esterase, putative	17
At5g65110	ACX2,ACX2 (ACYL-COA OXIDASE 2); acyl-CoA oxidase	17
At1g05570	CALS1,CALS1 (CALLOSE SYNTHASE 1); transferase, transferring glycosyl groups	17
At2g21960	unknown protein	17
At1g72820	mitochondrial substrate carrier family protein	17
At1g64650	unknown protein	18
At2g03520	ATUPS4,ATUPS4 (Arabidopsis thaliana ureide permease 4)	18
At2g37460	nodulin MtN21 family protein	18
At2g17630	phosphoserine aminotransferase, putative	18
At4g34050	caffeoyl-CoA 3-O-methyltransferase, putative	18
At3g17810	dihydroorotate dehydrogenase family protein / dihydroorotate oxidase family protein	18
At5g65020	ANNAT2,ANNAT2 (ANNEXIN ARABIDOPSIS 2); calcium ion binding / calcium-dependent phospholipid binding	18
At5g50110	methyltransferase-related	18
At4g14240	unknown protein	18
At1g54450	calcium-binding EF-hand family protein	18
At1g56330	SAR1,SAR1 (SECRETION-ASSOCIATED RAS); GTP binding	18
At1g53830	ATPME2,ATPME2 (Arabidopsis thaliana pectin methylesterase 2)	18
AtCg00660	RPL20,unknown protein	18
At2g45550	CYP76C4,CYP76C4 (cytochrome P450, family 76, subfamily C, polypeptide 4); oxygen binding	18
At5g04310	pectate lyase family protein	18
At3g04840	40S ribosomal protein S3A (RPS3aA)	18
At3g03250	UGP,UGP (UDP-glucose pyrophosphorylase); UTP:glucose-1-phosphate uridylyltransferase	18
At3g60210	chloroplast chaperonin 10, putative	18
At4g38490	unknown protein	18
At1g52060	unknown protein	18
At3g20370	meprin and TRAF homology domain-containing protein / MATH domain-containing protein	18
At3g58830	haloacid dehalogenase (HAD) superfamily protein	18
At4g27140	2S seed storage protein 1 / 2S albumin storage protein / NWMU1-2S albumin 1	18
At3g48930	EMB1080,EMB1080 (EMBRYO DEFECTIVE 1080); structural constituent of ribosome	18
At3g61440	ATCYSC1,ATCYSC1 (BETA-SUBSTITUTED ALA SYNTHASE 3;1),ATCYSC1 (BETA-SUBSTITUTED ALA SYNTHASE 3;1); L-3-cyanoalanine synthase/ cysteine synthase	18
At4g20460	NAD-dependent epimerase/dehydratase family protein	18
At5g38220	unknown protein	18
At2g43400	ETFQO,ETFQO (ELECTRON-TRANSFER FLAVOPROTEIN:UBIQUINONE	18

	OXIDOREDUCTASE); catalytic/ electron acceptor	
At1g51680	4CL1,4CL1 (4-COUMARATE:COA LIGASE 1),4CL1 (4-COUMARATE:COA LIGASE 1); 4-coumarate-CoA ligase	18
At3g26782	binding	18
At1g35720	ANNAT1,ANNAT1 (ANNEXIN ARABIDOPSIS 1); calcium ion binding / calcium-dependent phospholipid binding	18
At5g36270	dehydroascorbate reductase, putative	18
At4g33790	acyl CoA reductase, putative	18
At2g42200	squamosa promoter-binding protein-like 9 (SPL9)	18
At3g01120	MTO1,MTO1 (METHIONINE OVERACCUMULATION 1)	18
At2g19340	membrane protein, putative	18
At2g27130	protease inhibitor/seed storage/lipid transfer protein (LTP) family protein	18
At3g47540	chitinase, putative	18
At5g51700	PBS2,PBS2 (PPHB SUSCEPTIBLE 2)	19
At5g58950	protein kinase family protein	19
At5g47430	unknown protein	19
At2g38000	chaperone protein dnaJ-related	19
At5g07150	leucine-rich repeat family protein	19
At3g54230	nucleic acid binding	19
At5g04410	NAC2,NAC2 (Arabidopsis NAC domain containing protein 78); transcription factor	19
At4g03080	kelch repeat-containing serine/threonine phosphoesterase family protein	19
At1g55590	F-box family protein	19
At1g21050	unknown protein	19
At5g48480	unknown protein	19
At5g37710	lipase class 3 family protein / calmodulin-binding heat-shock protein, putative	19
At5g35940	jacalin lectin family protein	19
At5g04720	ADR1-L2,ADR1-L2 (ADR1-LIKE 2); ATP binding / nucleoside-triphosphatase/ nucleotide binding / protein binding	19
At4g29490	X-Pro dipeptidase	19
At5g16770	AtMYB9,AtMYB9 (myb domain protein 9); DNA binding,AtMYB9 (myb domain protein 9); DNA binding / transcription factor	19
At2g24520	AHA5,AHA5 (ARABIDOPSIS H(+)-ATPASE 5); ATPase	19
At3g13620	amino acid permease family protein	19
At3g04060	ANAC046,ANAC046 (Arabidopsis NAC domain containing protein 46); transcription factor	19
At5g58240	bis(5'-adenosyl)-triphosphatase, putative	19
At5g17300	myb family transcription factor	19
At1g51950	IAA18,IAA18 (indoleacetic acid-induced protein 18); transcription factor	19
At5g17280	unknown protein	19
At4g03190	GRH1,GRH1 (GRR1-LIKE PROTEIN 1); ubiquitin-protein ligase	19
At3g27610	unknown protein	19
At5g60100	APRR3,APRR3 (PSEUDO-RESPONSE REGULATOR 3); transcription regulator	19
At3g13030	hAT dimerisation domain-containing protein,protein dimerization	19
At4g16700	PSD1,phosphatidylserine decarboxylase	19
At1g58110	DNA binding / transcription factor,bZIP family transcription factor	19
At5g52580	unknown protein	19
At4g32680	unknown protein	19
At5g52210	ATGB1,ATGB1 (Arabidopsis thaliana GTP-binding protein 1),ATGB1	19

	(Arabidopsis thaliana GTP-binding protein 1); GTP binding	
At3g15095	unknown protein	19
At1g79060	unknown protein	19
At3g57390	AGL18,AGL18 (AGAMOUS-LIKE 18); transcription factor	19
At1g28960	ATNUDT15,ATNUDT15 (ARABIDOPSIS THALIANA NUDIX HYDROLASE HOMOLOG 15); hydrolase	19
At2g33845	DNA-binding protein-related	19
At1g70990	proline-rich family protein	19
At1g10270	GRP23,GRP23 (GLUTAMINE-RICH PROTEIN23); binding	19
At5g16440	IPP1,IPP1 (ISOPENTENYL DIPHOSPHATE ISOMERASE 1); isopentenyl-diphosphate delta-isomerase	19
At5g17640	unknown protein	19
At4g20010	PTAC9,OSB2,PTAC9 (PLASTID TRANSCRIPTIONALLY ACTIVE9); single-stranded DNA binding	19
At2g47930	ATAGP26,AGP26,AGP26/ATAGP26 (ARABINOGALACTAN PROTEINS 26)	19
At3g53620	ATPPA4,inorganic pyrophosphatase, putative (soluble) / pyrophosphate phospho-hydrolase, putative / PPase, putative	19
At4g24960	ATHVA22D,ATHVA22D (Arabidopsis thaliana HVA22 homologue D),unknown protein	19
At2g45160	scarecrow transcription factor family protein	19
At4g25390	protein kinase family protein	19
At5g64880	unknown protein	19
AtMg01370	ORF111D,unknown protein	20
At1g76340	integral membrane family protein	20
At3g05590	RPL18,RPL18 (RIBOSOMAL PROTEIN L18); structural constituent of ribosome	20
At2g01520	MLP328,major latex protein-related / MLP-related	20
At1g74520	ATHVA22A,ATHVA22A (Arabidopsis thaliana HVA22 homologue A)	20
At5g65640	BHLH093,BHLH093 (BETA HLH PROTEIN 93),BHLH093 (BETA HLH PROTEIN 93); DNA binding / transcription factor	20
At5g20290	40S ribosomal protein S8 (RPS8A)	20
At2g47320	peptidyl-prolyl cis-trans isomerase cyclophilin-type family protein	20
At3g52900	unknown protein	20
At4g09320	NDPK1,NDPK1 (nucleoside diphosphate kinase 1); ATP binding / nucleoside diphosphate kinase	20
At4g00430	TMP-C,TMP-C (plasma membrane intrinsic protein 1;4),TMP-C (plasma membrane intrinsic protein 1;4); water channel	20
At2g28510	Dof-type zinc finger domain-containing protein	20
At3g20320	TGD2,TGD2 (TRIGALACTOSYLDIACYLGLYCEROL2)	20
At4g11010	NDPK3,NDPK3 (NUCLEOSIDE DIPHOSPHATE KINASE 3); ATP binding / nucleoside diphosphate kinase	20
At1g61790	OST3/OST6 family protein	20
At2g26770	plectin-related	20
At5g48070	ATXTH20,ATXTH20 (XYLOGLUCAN ENDOTRANSGLUCOSYLASE/HYDROLASE 20); hydrolase, acting on glycosyl bonds	20
At1g72830	HAP2C,HAP2C (Heme activator protein (yeast) homolog 2C); transcription factor	20
At1g75210	5' nucleotidase family protein	20
At3g53340	CCAAT-box binding transcription factor, putative	20
At4g03210	XTH9,XTH9 (XYLOGLUCAN ENDOTRANSGLUCOSYLASE/HYDROLASE 9); hydrolase, acting on glycosyl bonds	20
At1g51070	basic helix-loop-helix (bHLH) family protein	20

At2g21050	amino acid permease, putative	20
At5g25810	TNY,TNY (TINY); DNA binding / transcription factor	20
At3g23000	CIPK7,CIPK7 (CBL-INTERACTING PROTEIN KINASE 7); kinase	20
At5g49910	cpHSC70-2,cpHSC70-2 (HEAT SHOCK PROTEIN 70-7); ATP binding / unfolded protein binding	20
At5g51550	phosphate-responsive 1 family protein	20
At3g11510	40S ribosomal protein S14 (RPS14B)	20
At2g19430	transducin family protein / WD-40 repeat family protein	20
At3g27420	unknown protein	20
At5g09380	DNA-directed RNA polymerase III RPC4 family protein	20
At1g05750	PDE247,PDE247 (PIGMENT DEFECTIVE 247); binding	20
At4g34710	ADC2,ADC2 (ARGININE DECARBOXYLASE 2)	20
At3g07340	basic helix-loop-helix (bHLH) family protein	20
At4g33250	EIF3K,EIF3K (eukaryotic translation initiation factor 3K)	20
At5g15770	ATGNA1,GCN5-related N-acetyltransferase (GNAT) family protein	20
At2g15550	transposable element gene At2g15550	20
At1g77940	60S ribosomal protein L30 (RPL30B)	20
At2g27720	60S acidic ribosomal protein P2 (RPP2A)	20

Significantly regulated genes at 3, 6, 9, 12, 15, 20 min:

>Genes significantly regulated at 3min	
At1g70080	terpene synthase/cyclase family protein
At2g16010	transposable element gene At2g16010
At2g38000	chaperone protein dnaJ-related
At3g54230	nucleic acid binding
At5g04410	NAC2,NAC2 (Arabidopsis NAC domain containing protein 78); transcription factor
At4g09320	NDPK1,NDPK1 (nucleoside diphosphate kinase 1); ATP binding / nucleoside diphosphate kinase
At3g19030	unknown protein
At3g50750	brassinosteroid signalling positive regulator-related
At4g11010	NDPK3,NDPK3 (NUCLEOSIDE DIPHOSPHATE KINASE 3); ATP binding / nucleoside diphosphate kinase
At1g52760	esterase/lipase/thioesterase family protein
At1g20390	transposable element gene At1g20390
At1g61790	OST3/OST6 family protein
At5g35820	transposable element gene At5g35820
At3g04840	40S ribosomal protein S3A (RPS3aA)
At3g27610	unknown protein
At3g60210	chloroplast chaperonin 10, putative
At3g13030	hAT dimerisation domain-containing protein,protein dimerization
At3g20370	meprin and TRAF homology domain-containing protein / MATH domain-containing protein
At5g66350	SHI,SHI (SHORT INTERNODES); transcription factor
At4g36350	PAP25,ATPAP25,ATPAP25/PAP25 (purple acid phosphatase 25); acid phosphatase/ protein serine/threonine phosphatase
At5g59080	unknown protein
At5g52580	unknown protein

At4g16700	PSD1,phosphatidylserine decarboxylase
At3g29690	transferase-related
At3g15095	unknown protein
At3g48930	EMB1080,EMB1080 (EMBRYO DEFECTIVE 1080); structural constituent of ribosome
At1g09570	PHYA,PHYA (PHYTOCHROME A); G-protein coupled photoreceptor/ signal transducer,unknown protein
At3g23000	CIPK7,CIPK7 (CBL-INTERACTING PROTEIN KINASE 7); kinase
At3g11510	40S ribosomal protein S14 (RPS14B)
At5g38220	unknown protein
At1g51680	4CL1,4CL1 (4-COUMARATE:COA LIGASE 1),4CL1 (4-COUMARATE:COA LIGASE 1); 4-coumarate-CoA ligase
At5g36270	dehydroascorbate reductase, putative
At5g48590	unknown protein
At4g20010	PTAC9,OSB2,PTAC9 (PLASTID TRANSCRIPTIONALLY ACTIVE9); single-stranded DNA binding
At2g24660	transposable element gene At2g24660
At2g15550	transposable element gene At2g15550
At2g27720	60S acidic ribosomal protein P2 (RPP2A)
At4g15270	glucosyltransferase-related
At2g42050	transposable element gene At2g42050
At3g05590	RPL18,RPL18 (RIBOSOMAL PROTEIN L18); structural constituent of ribosome
At1g32850	ubiquitin carboxyl-terminal hydrolase family protein
At5g37610	voltage-gated ion-selective channel
At2g01520	MLP328,major latex protein-related / MLP-related
At2g17630	phosphoserine aminotransferase, putative
At1g74520	ATHVA22A,ATHVA22A (Arabidopsis thaliana HVA22 homologue A)
At5g58950	protein kinase family protein
At1g27880	ATP-dependent DNA helicase, putative
At5g65640	BHLH093,BHLH093 (BETA HLH PROTEIN 93),BHLH093 (BETA HLH PROTEIN 93); DNA binding / transcription factor
At1g02900	RALFL1,RALFL1 (RALF-LIKE 1)
At3g07040	RPM1,RPM1 (RESISTANCE TO P. SYRINGAE PV MACULICOLA 1)
At5g20290	40S ribosomal protein S8 (RPS8A)
At1g54450	calcium-binding EF-hand family protein
At1g55590	F-box family protein
At1g56330	SAR1,SAR1 (SECRETION-ASSOCIATED RAS); GTP binding
At5g09360	LAC14,LAC14 (laccase 14); copper ion binding / oxidoreductase
At3g25490	wall-associated kinase, putative
At5g04720	ADR1-L2,ADR1-L2 (ADR1-LIKE 2); ATP binding / nucleoside-triphosphatase/ nucleotide binding / protein binding
At4g29490	X-Pro dipeptidase
At3g20320	TGD2,TGD2 (TRIGALACTOSYLDIACYLGLYCEROL2)
At2g24520	AHA5,AHA5 (ARABIDOPSIS H(+)-ATPASE 5); ATPase
At1g35570	transposable element gene At1g35570
At4g03190	GRH1,GRH1 (GRR1-LIKE PROTEIN 1); ubiquitin-protein ligase
At3g30800	transposable element gene At3g30800
At3g03250	UGP,UGP (UDP-glucose pyrophosphorylase); UTP:glucose-1-phosphate uridylyltransferase
At2g29940	PDR3,ATPDR3,ATPDR3/PDR3 (PLEIOTROPIC DRUG RESISTANCE 3); ATPase,

	coupled to transmembrane movement of substances
At1g19030	transposable element gene At1g19030
At1g52060	unknown protein
At5g46330	FLS2,FLS2 (FLAGELLIN-SENSITIVE 2); ATP binding / kinase/ protein binding / protein serine/threonine kinase/ transmembrane receptor protein serine/threonine kinase
At1g58110	DNA binding / transcription factor,bZIP family transcription factor
At1g51070	basic helix-loop-helix (bHLH) family protein
At4g27140	2S seed storage protein 1 / 2S albumin storage protein / NWMU1-2S albumin 1
At1g28960	ATNUDT15,ATNUDT15 (ARABIDOPSIS THALIANA NUDIX HYDROLASE HOMOLOG 15); hydrolase
At3g61440	ATCYSC1,ATCYSC1 (BETA-SUBSTITUTED ALA SYNTHASE 3;1),ATCYSC1 (BETA-SUBSTITUTED ALA SYNTHASE 3;1); L-3-cyanoalanine synthase/ cysteine synthase
At1g10270	GRP23,GRP23 (GLUTAMINE-RICH PROTEIN23); binding
At5g51550	phosphate-responsive 1 family protein
At2g19430	transducin family protein / WD-40 repeat family protein
At1g35720	ANNAT1,ANNAT1 (ANNEXIN ARABIDOPSIS 1); calcium ion binding / calcium-dependent phospholipid binding
At4g33250	EIF3K,EIF3K (eukaryotic translation initiation factor 3K)
At2g27130	protease inhibitor/seed storage/lipid transfer protein (LTP) family protein
At2g19340	membrane protein, putative
At5g15770	ATGNA1,GCN5-related N-acetyltransferase (GNAT) family protein
At1g77940	60S ribosomal protein L30 (RPL30B)
At4g30090	EMB1353,EMB1353 (EMBRYO DEFECTIVE 1353)

>Genes significantly regulated at 6min	
At2g37460	nodulin MtN21 family protein
At5g51700	PBS2,PBS2 (PPHB SUSCEPTIBLE 2)
At1g55120	ATFRUCT5,ATFRUCT5 (BETA-FRUCTOFURANOSIDASE 5); hydrolase, hydrolyzing O-glycosyl compounds / levanase,unknown protein
At3g17810	dihydroorotate dehydrogenase family protein / dihydroorotate oxidase family protein
At4g34050	caffeoyl-CoA 3-O-methyltransferase, putative
At3g25790	myb family transcription factor
At2g30860	ATGSTF9,ATGSTF9 (Arabidopsis thaliana Glutathione S-transferase (class phi) 9),ATGSTF9 (Arabidopsis thaliana Glutathione S-transferase (class phi) 9); glutathione transferase
At2g47320	peptidyl-prolyl cis-trans isomerase cyclophilin-type family protein
At4g13240	ROPGEF9,ATROPGEF9,ATROPGEF9/ROPGEF9 (KINASE PARTNER PROTEIN-LIKE); Rho guanyl-nucleotide exchange factor
At4g39780	AP2 domain-containing transcription factor, putative
At1g53830	ATPME2,ATPME2 (Arabidopsis thaliana pectin methylesterase 2)
At2g25980	jacalin lectin family protein
At5g35940	jacalin lectin family protein
At2g37430	zinc finger (C2H2 type) family protein (ZAT11)
At4g00430	TMP-C,TMP-C (plasma membrane intrinsic protein 1;4),TMP-C (plasma membrane intrinsic protein 1;4); water channel
At3g44960	unknown protein

At3g19030	unknown protein
At3g13620	amino acid permease family protein
At4g04600	transposable element gene At4g04600
At5g16770	AtMYB9,AtMYB9 (myb domain protein 9); DNA binding,AtMYB9 (myb domain protein 9); DNA binding / transcription factor
At2g27830	unknown protein
At1g35290	thioesterase family protein
At1g64590	short-chain dehydrogenase/reductase (SDR) family protein
At5g35820	transposable element gene At5g35820
At5g11930	glutaredoxin family protein
At5g17280	unknown protein
At5g55750	hydroxyproline-rich glycoprotein family protein
At5g36940	CAT3,CAT3 (CATIONIC AMINO ACID TRANSPORTER 3); cationic amino acid transporter
At1g68360	zinc finger protein-related
At1g22400	UGT85A1,ATUGT85A1,UGT85A1 (UDP-glucosyl transferase 85A1); UDP-glycosyltransferase/ transferase, transferring glycosyl groups / transferase, transferring hexosyl groups
At3g25980	mitotic spindle checkpoint protein, putative (MAD2)
At1g32920	unknown protein
At3g57790	glycoside hydrolase family 28 protein / polygalacturonase (pectinase) family protein
At1g09575	unknown protein
At3g20370	meprin and TRAF homology domain-containing protein / MATH domain-containing protein
At4g03210	XTH9,XTH9 (XYLOGLUCAN ENDOTRANSGLUCOSYLASE/HYDROLASE 9); hydrolase, acting on glycosyl bonds
At5g10210	unknown protein
At1g50350	unknown protein
At1g17810	BETA-TIP,BETA-TIP (BETA-TONOPLAST INTRINSIC PROTEIN); water channel
At3g20740	FIE,FIE (FERTILIZATION-INDEPENDENT ENDOSPERM 1); nucleotide binding / transcription factor
At3g29690	transferase-related
At2g21050	amino acid permease, putative
At3g57390	AGL18,AGL18 (AGAMOUS-LIKE 18); transcription factor
At5g17880	CSA1,CSA1 (CONSTITUTIVE SHADE-AVOIDANCE1); ATP binding / protein binding / transmembrane receptor
At3g23000	CIPK7,CIPK7 (CBL-INTERACTING PROTEIN KINASE 7); kinase
At4g07586	transposable element gene At4g07586
At3g60070	lactose permease-related
At1g51680	4CL1,4CL1 (4-COUMARATE:COA LIGASE 1),4CL1 (4-COUMARATE:COA LIGASE 1); 4-coumarate-CoA ligase
At2g43400	ETFQO,ETFQO (ELECTRON-TRANSFER FLAVOPROTEIN:UBIQUINONE OXIDOREDUCTASE); catalytic/ electron acceptor
At5g09380	DNA-directed RNA polymerase III RPC4 family protein
At5g38220	unknown protein
At5g66800	unknown protein
At1g70985	hydroxyproline-rich glycoprotein family protein
At5g36270	dehydroascorbate reductase, putative
At3g23080	unknown protein

At1g60670	unknown protein
At1g54120	unknown protein
At2g24660	transposable element gene At2g24660
At2g42200	squamosa promoter-binding protein-like 9 (SPL9)
At2g45160	scarecrow transcription factor family protein
At3g01120	MTO1,MTO1 (METHIONINE OVERACCUMULATION 1)
At3g10740	ASD1,ASD1 (ALPHA-L-ARABINOFURANOSIDASE); hydrolase, acting on glycosyl bonds
At1g12520	CCS1,CCS1 (copper chaperone for superoxide dismutase 1); superoxide dismutase copper chaperone
At5g56070	unknown protein
At1g64650	unknown protein
At3g21340	leucine-rich repeat protein kinase, putative
At2g24330	unknown protein
AtMg01370	ORF111D,unknown protein
At1g05920	DNA binding
At5g37610	voltage-gated ion-selective channel
At4g37610	BT5,BT5 (BTB and TAZ domain protein 5); protein binding / transcription regulator
At4g03080	kelch repeat-containing serine/threonine phosphoesterase family protein
At4g14240	unknown protein
At3g25490	wall-associated kinase, putative
At5g37710	lipase class 3 family protein / calmodulin-binding heat-shock protein, putative
At2g28510	Dof-type zinc finger domain-containing protein
At2g24520	AHA5,AHA5 (ARABIDOPSIS H(+)-ATPASE 5); ATPase
At1g51950	IAA18,IAA18 (indoleacetic acid-induced protein 18); transcription factor
At3g16460	jacalin lectin family protein
At1g08750	GPI-anchor transamidase, putative
At3g61430	PIP1A,PIP1A (plasma membrane intrinsic protein 1;1),PIP1A (plasma membrane intrinsic protein 1;1); water channel
AtCg00080	PSBI,unknown protein
At5g11510	MYB3R-4,MYB3R-4 (C-MYB-LIKE TRANSCRIPTION FACTOR 3R-4, myb domain protein 3R-4); DNA binding / transcription factor
At3g53340	CCAAT-box binding transcription factor, putative
At1g52060	unknown protein
At4g38490	unknown protein
At3g49740	pentatricopeptide (PPR) repeat-containing protein
At3g61440	ATCYSC1,ATCYSC1 (BETA-SUBSTITUTED ALA SYNTHASE 3;1),ATCYSC1 (BETA-SUBSTITUTED ALA SYNTHASE 3;1); L-3-cyanoalanine synthase/ cysteine synthase
At2g46870	NGA1,NGA1 (NGATHA1); transcription factor
At4g37150	esterase, putative
At5g17640	unknown protein
At3g62630	unknown protein
AtCg00180	RPOC1,unknown protein
At2g07740	transposable element gene At2g07740
At1g05570	CALS1,CALS1 (CALLOSE SYNTHASE 1); transferase, transferring glycosyl groups
At2g21960	unknown protein

At4g25390	protein kinase family protein
At4g13880	leucine-rich repeat family protein
At5g36870	ATGSL09,ATGSL09 (GLUCAN SYNTHASE-LIKE 9); 1,3-beta-glucan synthase
At1g65860	flavin-containing monooxygenase family protein / FMO family protein
At2g45430	DNA-binding protein-related
At1g27900	RNA helicase, putative
At4g13440	calcium-binding EF hand family protein
At2g42660	myb family transcription factor
At5g09800	U-box domain-containing protein
At5g07150	leucine-rich repeat family protein
At5g50110	methyltransferase-related
At4g05610	transposable element gene At4g05610
At5g04410	NAC2,NAC2 (Arabidopsis NAC domain containing protein 78); transcription factor
At1g18100	E12A11,E12A11; phosphatidylethanolamine binding
At1g51990	O-methyltransferase family 2 protein
At5g62530	ALDH12A1,ALDH12A1 (Aldehyde dehydrogenase 12A1); 1-pyrroline-5-carboxylate dehydrogenase/ 3-chloroallyl aldehyde dehydrogenase
At2g24790	COL3,COL3 (CONSTANS-LIKE 3); protein binding / transcription factor/ zinc ion binding,COL3 (CONSTANS-LIKE 3); zinc ion binding
At4g30450	glycine-rich protein
At2g34330	unknown protein
At1g62660	beta-fructosidase (BFRUCT3) / beta-fructofuranosidase / invertase, vacuolar
At4g30460	glycine-rich protein
At1g20390	transposable element gene At1g20390
At1g52760	esterase/lipase/thioesterase family protein
At3g02140	TMAC2,TMAC2 (TWO OR MORE ABRES-CONTAINING GENE 2)
At2g46190	unknown protein
At5g04310	pectate lyase family protein
At1g21920	MORN (Membrane Occupation and Recognition Nexus) repeat-containing protein /phosphatidylinositol-4-phosphate 5-kinase-related
At3g13030	hAT dimerisation domain-containing protein,protein dimerization
At4g01450	nodulin MtN21 family protein
At2g01150	RHA2B,RHA2B (RING-H2 FINGER PROTEIN 2B); protein binding / zinc ion binding
AtCg00300	YCF9,unknown protein
At4g36350	PAP25,ATPAP25,ATPAP25/PAP25 (purple acid phosphatase 25); acid phosphatase/ protein serine/threonine phosphatase
At1g64370	unknown protein
At5g35980	kinase,protein kinase family protein
At1g79060	unknown protein
At3g58830	haloacid dehalogenase (HAD) superfamily protein
At5g36160	aminotransferase-related
At5g10820	integral membrane transporter family protein
At2g33845	DNA-binding protein-related
At1g70990	proline-rich family protein
At1g63690	protease-associated (PA) domain-containing protein
At1g61410	tolA protein-related
At4g37540	LBD39,LOB domain protein 39 / lateral organ boundaries domain protein 39 (LBD39)

At5g37055	ATSWC6,zinc finger (HIT type) family protein
At3g09260	PYK10,PYK10 (phosphate starvation-response 3.1); hydrolase, hydrolyzing O-glycosyl compounds
At5g16440	IPP1,IPP1 (ISOPENTENYL DIPHOSPHATE ISOMERASE 1); isopentenyl-diphosphate delta-isomerase
At5g49910	cpHSC70-2,cpHSC70-2 (HEAT SHOCK PROTEIN 70-7); ATP binding / unfolded protein binding
At2g37440	endonuclease/exonuclease/phosphatase family protein
At1g19850	MP,MP (MONOPTEROS); transcription factor
At5g48590	unknown protein
At1g25550	myb family transcription factor
AtCg00780	RPL14,unknown protein
At4g13650	pentatricopeptide (PPR) repeat-containing protein
At2g44440	emsy N terminus domain-containing protein / ENT domain-containing protein
At2g03520	ATUPS4,ATUPS4 (Arabidopsis thaliana ureide permease 4)
At1g32850	ubiquitin carboxyl-terminal hydrolase family protein
At3g05950	germin-like protein, putative
At5g47430	unknown protein
At3g07040	RPM1,RPM1 (RESISTANCE TO P. SYRINGAE PV MACULICOLA 1)
At1g61360	S-locus lectin protein kinase family protein
At3g06380	ATTLP9,ATTLP9/AtTLP9 (TUBBY LIKE PROTEIN 9, TUBBY-LIKE PROTEIN 9); phosphoric diester hydrolase/ protein binding / transcription factor
At4g23320	protein kinase family protein
At2g06220	transposable element gene At2g06220
At3g48990	AMP-dependent synthetase and ligase family protein
At2g40890	CYP98A3,CYP98A3 (cytochrome P450, family 98, subfamily A, polypeptide 3); p-coumarate 3-hydroxylase
At5g14980	esterase/lipase/thioesterase family protein
At5g17300	myb family transcription factor
At5g58240	bis(5'-adenosyl)-triphosphatase, putative
At4g36770	UDP-glucoronosyl/UDP-glucosyl transferase family protein
At1g15730	PRLI-interacting factor L, putative
At5g60100	APRR3,APRR3 (PSEUDO-RESPONSE REGULATOR 3); transcription regulator
At4g34000	DPBF5,ABF3,ABF3/DPBF5 (ABSCISIC ACID RESPONSIVE ELEMENTS-BINDING FACTOR 3); DNA binding / protein binding / transcription factor/ transcriptional activator
At3g57450	unknown protein
At5g52210	ATGB1,ATGB1 (Arabidopsis thaliana GTP-binding protein 1),ATGB1 (Arabidopsis thaliana GTP-binding protein 1); GTP binding
AtMg01040	ORF107F,unknown protein
At3g63110	ATIPT3,ATIPT3 (Arabidopsis thaliana isopentenyltransferase 3); transferase, transferring alkyl or aryl (other than methyl) groups
At1g10810	aldo/keto reductase family protein
At1g20070	unknown protein
AtCg00510	PSAI,unknown protein
At3g26782	binding
At1g07150	MAPKKK13,MAPKKK13 (Mitogen-activated protein kinase kinase kinase 13); kinase
At5g65110	ACX2,ACX2 (ACYL-COA OXIDASE 2); acyl-CoA oxidase

At5g67420	LBD37,LOB domain protein 37 / lateral organ boundaries domain protein 37 (LBD37)
At2g45930	unknown protein
At1g35720	ANNAT1,ANNAT1 (ANNEXIN ARABIDOPSIS 1); calcium ion binding / calcium-dependent phospholipid binding
At1g78090	ATTPPB,ATTPPB (TREHALOSE-6-PHOSPHATE PHOSPHATASE)
At4g25100	FSD1,FSD1 (FE SUPEROXIDE DISMUTASE 1); iron superoxide dismutase
At2g47930	ATAGP26,AGP26,AGP26/ATAGP26 (ARABINOGALACTAN PROTEINS 26)
At4g33790	acyl CoA reductase, putative
At3g53620	ATPPA4,inorganic pyrophosphatase, putative (soluble) / pyrophosphate phospho-hydrolase, putative / PPase, putative
At1g73000	unknown protein
At2g19340	membrane protein, putative
At2g27130	protease inhibitor/seed storage/lipid transfer protein (LTP) family protein
At3g49940	LBD38,LOB domain protein 38 / lateral organ boundaries domain protein 38 (LBD38)
At5g42830	transferase family protein
At5g64880	unknown protein

>Genes significantly regulated at 9min	
At3g60690	auxin-responsive family protein
At5g17490	RGL3,RGL3 (RGA-LIKE 3); transcription factor
At4g05390	ATRFNR1,ATRFNR1 (ROOT FNR 1); oxidoreductase
At5g19260	unknown protein
At4g37610	BT5,BT5 (BTB and TAZ domain protein 5); protein binding / transcription regulator
At3g25790	myb family transcription factor
At5g09800	U-box domain-containing protein
At1g14540	anionic peroxidase, putative
At1g27880	ATP-dependent DNA helicase, putative
At3g07350	unknown protein
At2g30040	MAPKKK14,MAPKKK14 (Mitogen-activated protein kinase kinase kinase 14); kinase
At4g40070	protein binding / zinc ion binding
At3g04980	DNAJ heat shock N-terminal domain-containing protein
At3g16150	L-asparaginase, putative / L-asparagine amidohydrolase, putative
At3g50900	unknown protein
At3g28510	AAA-type ATPase family protein
At3g18710	U-box domain-containing protein
At5g41670	6-phosphogluconate dehydrogenase family protein
At5g28610	unknown protein
At2g43000	ANAC042,ANAC042 (Arabidopsis NAC domain containing protein 42); transcription factor
At4g34760	auxin-responsive family protein
At5g39580	peroxidase,peroxidase, putative
At1g64190	6-phosphogluconate dehydrogenase family protein
At1g13300	myb family transcription factor
At4g36880	CP1,cysteine proteinase, putative
At1g68360	zinc finger protein-related
At4g37240	unknown protein

At5g40850	UPM1,UPM1 (UROPHORPHYRIN METHYLASE 1); uroporphyrin-III C-methyltransferase
At1g14550	anionic peroxidase, putative
At1g32920	unknown protein
At1g49230	zinc finger (C3HC4-type RING finger) family protein
At5g45340	CYP707A3,CYP707A3 (cytochrome P450, family 707, subfamily A, polypeptide 3); oxygen binding
At5g13110	G6PD2,G6PD2 (GLUCOSE-6-PHOSPHATE DEHYDROGENASE 2); glucose-6-phosphate 1-dehydrogenase
At4g04840	methionine sulfoxide reductase domain-containing protein / SeIR domain-containing protein
At1g26380	FAD-binding domain-containing protein
At5g10210	unknown protein
At1g24280	G6PD3,G6PD3 (GLUCOSE-6-PHOSPHATE DEHYDROGENASE 3); glucose-6-phosphate 1-dehydrogenase
At4g39950	CYP79B2,CYP79B2 (cytochrome P450, family 79, subfamily B, polypeptide 2); oxygen binding
At5g40990	GLIP1,GLIP1 (GDSL LIPASE1); carboxylic ester hydrolase
At4g37540	LBD39,LOB domain protein 39 / lateral organ boundaries domain protein 39 (LBD39)
At4g39940	AKN2,AKN2 (APS-KINASE 2); ATP binding / kinase/ transferase, transferring phosphorus-containing groups
At5g01740	unknown protein
At5g67420	LBD37,LOB domain protein 37 / lateral organ boundaries domain protein 37 (LBD37)
At5g54170	unknown protein
At5g04840	bZIP protein
At1g25550	myb family transcription factor
At1g49000	unknown protein
At1g69760	unknown protein
At2g15620	NIR1,NIR1 (NITRITE REDUCTASE); ferredoxin-nitrate reductase
At3g49940	LBD38,LOB domain protein 38 / lateral organ boundaries domain protein 38 (LBD38)
At3g47540	chitinase, putative
At3g18560	unknown protein
At1g78050	phosphoglycerate/bisphosphoglycerate mutase family protein
At5g19120	pepsin A
At5g42830	transferase family protein

>Genes significantly regulated at 12min	
At2g36320	zinc finger (AN1-like) family protein
At5g62720	integral membrane HPP family protein
At1g64380	AP2 domain-containing transcription factor, putative
At5g65300	unknown protein
At3g25790	myb family transcription factor
At1g69450	unknown protein
At3g07350	unknown protein
At4g36040	DNAJ heat shock N-terminal domain-containing protein (J11)
At4g39780	AP2 domain-containing transcription factor, putative
At2g28190	CSD2,CSD2 (COPPER/ZINC SUPEROXIDE DISMUTASE 2); copper, zinc superoxide

	dismutase
At3g19030	unknown protein
At2g18030	peptide methionine sulfoxide reductase family protein
At2g33390	unknown protein
At1g63940	monodehydroascorbate reductase, putative
At5g41670	6-phosphogluconate dehydrogenase family protein
At2g22500	mitochondrial substrate carrier family protein
At2g27830	unknown protein
At5g28610	unknown protein
At5g11930	glutaredoxin family protein
At1g64190	6-phosphogluconate dehydrogenase family protein
At3g04530	PPCK2,PPCK2 (PHOSPHOENOLPYRUVATE CARBOXYLASE KINASE 2); kinase
At1g32920	unknown protein
At2g24550	unknown protein
At5g45340	CYP707A3,CYP707A3 (cytochrome P450, family 707, subfamily A, polypeptide 3); oxygen binding
At1g15100	RHA2A,RHA2A (RING-H2 finger A2A); protein binding / zinc ion binding
At5g10210	unknown protein
At3g08650	metal transporter family protein
At1g15550	GA4,GA4 (GA REQUIRING 4); gibberellin 3-beta-dioxygenase
At4g10310	HKT1,HKT1 (HIGH-AFFINITY K+ TRANSPORTER 1); sodium ion transporter
At1g62180	APR2,APR2 (5'ADENYLYLPHOSPHOSULFATE REDUCTASE 2)
At4g18340	glycosyl hydrolase family 17 protein
At4g03510	RMA1,RMA1 (Ring finger protein with Membrane Anchor 1); protein binding / ubiquitin-protein ligase/ zinc ion binding
At4g25880	APUM6,APUM6 (ARABIDOPSIS PUMILIO 6); RNA binding
At1g08090	ATNRT2:1,ATNRT2:1 (Arabidopsis thaliana high affinity nitrate transporter 2.1); nitrate transporter
At1g60670	unknown protein
At1g69760	unknown protein
At1g19050	ARR7,ARR7 (RESPONSE REGULATOR 7); transcription regulator/ two-component response regulator
At5g10030	TGA4,TGA4 (TGACG MOTIF-BINDING FACTOR 4); DNA binding / calmodulin binding / transcription factor
At3g62930	glutaredoxin family protein
At4g09900	hydrolase, alpha/beta fold family protein
At4g02380	SAG21,SAG21 (SENESCENCE-ASSOCIATED GENE 21)
At2g39370	unknown protein
At2g27510	ATFD3,ATFD3 (FERREDOXIN 3); electron carrier
At5g19260	unknown protein
At4g05390	ATRFNR1,ATRFNR1 (ROOT FNR 1); oxidoreductase
At4g37610	BT5,BT5 (BTB and TAZ domain protein 5); protein binding / transcription regulator
At4g00416	MBD3,MBD3 (methyl-CpG-binding domain 3); DNA binding
At1g72200	zinc finger (C3HC4-type RING finger) family protein
At4g40070	protein binding / zinc ion binding
At4g36010	pathogenesis-related thaumatin family protein
At2g38510	MATE efflux protein-related
At5g40470	unknown protein
At2g04730	pseudogene
At3g62410	CP12-2

At3g20320	TGD2,TGD2 (TRIGALACTOSYLDIACYLGLYCEROL2)
At2g45550	CYP76C4,CYP76C4 (cytochrome P450, family 76, subfamily C, polypeptide 4); oxygen binding
At1g12820	AFB3,AFB3 (AUXIN SIGNALING F-BOX 3); auxin binding / ubiquitin-protein ligase
At1g66140	ZFP4,ZFP4 (ZINC FINGER PROTEIN 4); nucleic acid binding / transcription factor/ zinc ion binding
At2g28910	CXIP4,CXIP4 (CAX INTERACTING PROTEIN 4); nucleic acid binding / zinc ion binding
At3g52950	CBS domain-containing protein / octicosapeptide/Phox/Bemp1 (PB1) domain-containing protein
At4g30190	AHA2,AHA2 (Arabidopsis H(+)-ATPase 2); ATPase
At1g13300	myb family transcription factor
At5g40850	UPM1,UPM1 (UROPHORPHYRIN METHYLASE 1); uroporphyrin-III C-methyltransferase
At5g15330	SPX (SYG1/Pho81/XPR1) domain-containing protein
At1g75210	5' nucleotidase family protein
At5g13110	G6PD2,G6PD2 (GLUCOSE-6-PHOSPHATE DEHYDROGENASE 2); glucose-6-phosphate 1-dehydrogenase
At1g24280	G6PD3,G6PD3 (GLUCOSE-6-PHOSPHATE DEHYDROGENASE 3); glucose-6-phosphate 1-dehydrogenase
At1g55310	SR33,SR33 (SC35-like splicing factor 33); RNA binding
At5g03380	heavy-metal-associated domain-containing protein,metal ion binding
At3g61440	ATCYSC1,ATCYSC1 (BETA-SUBSTITUTED ALA SYNTHASE 3;1),ATCYSC1 (BETA-SUBSTITUTED ALA SYNTHASE 3;1); L-3-cyanoalanine synthase/ cysteine synthase
At3g47050	glycosyl hydrolase family 3 protein
At1g16170	unknown protein
At3g62630	unknown protein
At4g38340	RWP-RK domain-containing protein
At5g39870	unknown protein
At5g04840	bZIP protein
At3g46130	MYB111,MYB111 (myb domain protein 111),MYB111 (myb domain protein 111); DNA binding,MYB111 (myb domain protein 111); DNA binding / transcription factor
At2g15620	NIR1,NIR1 (NITRITE REDUCTASE); ferredoxin-nitrate reductase
At3g18560	unknown protein
At1g21000	unknown protein,zinc-binding family protein
At3g60690	auxin-responsive family protein
At5g28620	protein kinase C-related
At5g44140	ATPHB7,ATPHB7 (PROHIBITIN 7)
At1g80460	NHO1,NHO1 (NONHOST RESISTANCE TO P. S. PHASEOLICOLA 1); carbohydrate kinase
At4g24670	alliinase family protein
At5g09800	U-box domain-containing protein
At3g28510	AAA-type ATPase family protein
At5g35630	GS2,GS2 (GLUTAMINE SYNTHETASE 2),GS2 (GLUTAMINE SYNTHETASE 2); glutamate-ammonia ligase
At1g52760	esterase/lipase/thioesterase family protein
At4g34760	auxin-responsive family protein
At4g37240	unknown protein
At3g14050	RSH2,RSH2 (RELA-SPOT HOMOLOG); catalytic
At1g67920	unknown protein

At1g30510	ATRFNR2,ATRFNR2 (ROOT FNR 2); oxidoreductase
At1g64370	unknown protein
At4g32680	unknown protein
At4g37180	myb family transcription factor
At5g58530	glutaredoxin family protein
At3g15760	unknown protein
At4g37540	LBD39,LOB domain protein 39 / lateral organ boundaries domain protein 39 (LBD39)
At5g15830	ATBZIP3,bZIP transcription factor family protein
At1g68880	ATBZIP,bZIP transcription factor family protein
At5g01740	unknown protein
At5g54170	unknown protein
At2g18160	GBF5,ATBZIP2,GBF5 (G-box binding factor 5); DNA binding / transcription factor
At1g61930	unknown protein
At4g32950	protein phosphatase 2C, putative / PP2C, putative
At3g19190	unknown protein
At1g25550	myb family transcription factor
At1g49000	unknown protein
At5g57655	xylose isomerase family protein
At5g19120	pepsin A
At2g35270	DNA-binding protein-related
At5g67390	unknown protein
At5g58950	protein kinase family protein
At5g24160	squalene monooxygenase 1,2 / squalene epoxidase 1,2 (SQP1,2)
At4g09620	unknown protein
At2g30040	MAPKKK14,MAPKKK14 (Mitogen-activated protein kinase kinase kinase 14); kinase
At2g31940	oxidoreductase/ transition metal ion binding
At1g24440	protein binding / zinc ion binding
At1g43710	EMB1075,EMB1075 (EMBRYO DEFECTIVE 1075); carboxy-lyase
At2g40890	CYP98A3,CYP98A3 (cytochrome P450, family 98, subfamily A, polypeptide 3); p-coumarate 3-hydroxylase
At1g49860	ATGSTF14,ATGSTF14 (Arabidopsis thaliana Glutathione S-transferase (class phi) 14); glutathione transferase
At3g19010	oxidoreductase, 2OG-Fe(II) oxygenase family protein
At3g50900	unknown protein
At3g18710	U-box domain-containing protein
At3g49760	ATBZIP5,bZIP transcription factor family protein
At1g72240	unknown protein
At3g17510	CIPK1,CIPK1 (CBL-INTERACTING PROTEIN KINASE 1); kinase
At4g13430	aconitase family protein / aconitate hydratase family protein
At1g49230	zinc finger (C3HC4-type RING finger) family protein
At4g25835	AAA-type ATPase family protein
At2g02630	DC1 domain-containing protein
At3g57450	unknown protein
At3g53150	UGT73D1,UGT73D1 (UDP-glucosyl transferase 73D1); UDP-glycosyltransferase
At2g19460	unknown protein
At5g65210	TGA1,DNA binding / calmodulin binding / transcription factor,bZIP family transcription factor (TGA1)
At5g06800	myb family transcription factor
At5g12860	DIT1,DIT1 (DICARBOXYLATE TRANSPORTER 1); oxoglutarate:malate

	antiporter,unknown protein
At4g02920	unknown protein
At4g31910	transferase family protein
At5g67420	LBD37,LOB domain protein 37 / lateral organ boundaries domain protein 37 (LBD37)
At1g68670	myb family transcription factor
At4g20000	VQ motif-containing protein
At2g47930	ATAGP26,AGP26,AGP26/ATAGP26 (ARABINOGALACTAN PROTEINS 26)
At4g24960	ATHVA22D,ATHVA22D (Arabidopsis thaliana HVA22 homologue D),unknown protein
At3g49940	LBD38,LOB domain protein 38 / lateral organ boundaries domain protein 38 (LBD38)
At1g78050	phosphoglycerate/bisphosphoglycerate mutase family protein
At1g72820	mitochondrial substrate carrier family protein

>Genes significantly regulated at 15min	
At5g23010	MAM1,MAM1 (2-isopropylmalate synthase 3); 2-isopropylmalate synthase
At5g23020	MAM-L,MAM-L (METHYLTHIOALKYMALATE SYNTHASE-LIKE); 2-isopropylmalate synthase
At5g62720	integral membrane HPP family protein
At3g25790	myb family transcription factor
At2g40750	WRKY54,WRKY54 (WRKY DNA-binding protein 54); transcription factor
At3g60750	transketolase, putative
At1g69450	unknown protein
At3g07350	unknown protein
At4g39780	AP2 domain-containing transcription factor, putative
At4g36040	DNAJ heat shock N-terminal domain-containing protein (J11)
At1g77760	NIA1,NIA1 (NITRATE REDUCTASE 1)
At2g28190	CSD2,CSD2 (COPPER/ZINC SUPEROXIDE DISMUTASE 2); copper, zinc superoxide dismutase
At3g19030	unknown protein
At3g16150	L-asparaginase, putative / L-asparagine amidohydrolase, putative
At1g80380	phosphoribulokinase/uridine kinase-related
At1g63940	monodehydroascorbate reductase, putative
At5g41670	6-phosphogluconate dehydrogenase family protein
At2g22500	mitochondrial substrate carrier family protein
At2g27830	unknown protein
At5g64120	peroxidase, putative
At5g28610	unknown protein
At4g31730	GDU1,GDU1 (GLUTAMINE DUMPER 1)
At5g11930	glutaredoxin family protein
At1g64190	6-phosphogluconate dehydrogenase family protein
At3g04530	PPCK2,PPCK2 (PHOSPHOENOLPYRUVATE CARBOXYLASE KINASE 2); kinase
At3g25980	mitotic spindle checkpoint protein, putative (MAD2)
At1g32920	unknown protein
At2g24550	unknown protein
At5g45340	CYP707A3,CYP707A3 (cytochrome P450, family 707, subfamily A, polypeptide 3); oxygen binding
At1g73260	trypsin and protease inhibitor family protein / Kunitz family protein

At1g15100	RHA2A,RHA2A (RING-H2 finger A2A); protein binding / zinc ion binding
At5g10210	unknown protein
At5g59080	unknown protein
At1g15550	GA4,GA4 (GA REQUIRING 4); gibberellin 3-beta-dioxygenase
At2g27570	sulfotransferase family protein
At1g62180	APR2,APR2 (5'ADENYLYLPHOSPHOSULFATE REDUCTASE 2)
At4g10310	HKT1,HKT1 (HIGH-AFFINITY K+ TRANSPORTER 1); sodium ion transporter
At4g18340	glycosyl hydrolase family 17 protein
At4g10390	protein kinase family protein
At1g77400	unknown protein
At5g08010	unknown protein
At4g03510	RMA1,RMA1 (Ring finger protein with Membrane Anchor 1); protein binding / ubiquitin-protein ligase/ zinc ion binding
At5g60770	ATNRT2.4,ATNRT2.4 (Arabidopsis thaliana high affinity nitrate transporter 2.4); nitrate transporter
At1g64140	unknown protein
At1g49160	WNK7,WNK7 (Arabidopsis WNK kinase 7); kinase
At1g08090	ATNRT2:1,ATNRT2:1 (Arabidopsis thaliana high affinity nitrate transporter 2.1); nitrate transporter
At1g16420	ATMC8,latex-abundant protein, putative (AMC8) / caspase family protein
At5g47060	senescence-associated protein-related
At1g69760	unknown protein
At4g24620	PGI1,PGI1 (CHLOROPLASTIC PHOSPHOGLUCOSE ISOMERASE)
At5g10030	TGA4,TGA4 (TGACG MOTIF-BINDING FACTOR 4); DNA binding / calmodulin binding / transcription factor
At3g62930	glutaredoxin family protein
At1g18485	pentatricopeptide (PPR) repeat-containing protein
At3g28200	peroxidase, putative
At5g56870	BGAL4,beta-galactosidase, putative / lactase, putative
At4g02380	SAG21,SAG21 (SENESCENCE-ASSOCIATED GENE 21)
At2g25450	2-oxoglutarate-dependent dioxygenase, putative
At2g27510	ATFD3,ATFD3 (FERREDOXIN 3); electron carrier
At5g19260	unknown protein
At4g05390	ATRFNR1,ATRFNR1 (ROOT FNR 1); oxidoreductase
At4g37610	BT5,BT5 (BTB and TAZ domain protein 5); protein binding / transcription regulator
At5g65020	ANNAT2,ANNAT2 (ANNEXIN ARABIDOPSIS 2); calcium ion binding / calcium-dependent phospholipid binding
At1g72200	zinc finger (C3HC4-type RING finger) family protein
At5g24890	unknown protein
At2g38510	MATE efflux protein-related
At4g36010	pathogenesis-related thaumatin family protein
At4g40070	protein binding / zinc ion binding
At3g62410	CP12-2
At1g12820	AFB3,AFB3 (AUXIN SIGNALING F-BOX 3); auxin binding / ubiquitin-protein ligase
At1g66140	ZFP4,ZFP4 (ZINC FINGER PROTEIN 4); nucleic acid binding / transcription factor/ zinc ion binding
At1g51950	IAA18,IAA18 (indoleacetic acid-induced protein 18); transcription factor
At4g16680	RNA helicase, putative
At4g18350	NCED2,NCED2 (NINE-CIS-EPOXYCAROTENOID DIOXYGENASE 2)

At3g52950	CBS domain-containing protein / octicosapeptide/Phox/Bemp1 (PB1) domain-containing protein
At1g13300	myb family transcription factor
At3g61430	PIP1A,PIP1A (plasma membrane intrinsic protein 1;1),PIP1A (plasma membrane intrinsic protein 1;1); water channel
At3g23030	IAA2,IAA2 (indoleacetic acid-induced protein 2); transcription factor
At2g19110	HMA4,HMA4 (Heavy metal ATPase 4); cadmium-transporting ATPase
At5g15330	SPX (SYG1/Pho81/XPR1) domain-containing protein
At5g40850	UPM1,UPM1 (UROPHORPHYRIN METHYLASE 1); uroporphyrin-III C-methyltransferase
At1g74940	senescence-associated protein-related
At3g29760	NLI interacting factor (NIF) family protein
At5g13110	G6PD2,G6PD2 (GLUCOSE-6-PHOSPHATE DEHYDROGENASE 2); glucose-6-phosphate 1-dehydrogenase
At1g19030	transposable element gene At1g19030
At5g19010	MPK16,MPK16 (mitogen-activated protein kinase 16); MAP kinase
At4g04040	MEE51,MEE51 (maternal effect embryo arrest 51); diphosphate-fructose-6-phosphate 1-phosphotransferase
AtCg00420	NDHJ,unknown protein
At1g24280	G6PD3,G6PD3 (GLUCOSE-6-PHOSPHATE DEHYDROGENASE 3); glucose-6-phosphate 1-dehydrogenase
At5g03380	heavy-metal-associated domain-containing protein,metal ion binding
At3g62630	unknown protein
At4g38340	RWP-RK domain-containing protein
At2g48080	oxidoreductase, 2OG-Fe(II) oxygenase family protein
At5g04840	bZIP protein
At3g46130	MYB111,MYB111 (myb domain protein 111),MYB111 (myb domain protein 111); DNA binding,MYB111 (myb domain protein 111); DNA binding / transcription factor
At2g15620	NIR1,NIR1 (NITRITE REDUCTASE); ferredoxin-nitrate reductase
At2g07740	transposable element gene At2g07740
At3g18560	unknown protein
At1g21000	unknown protein,zinc-binding family protein
At3g60690	auxin-responsive family protein
At5g18670	BMY3,BMY3 (beta-amylase 3); beta-amylase
At1g15380	lactoylglutathione lyase,lactoylglutathione lyase family protein / glyoxalase I family protein
At1g80460	NHO1,NHO1 (NONHOST RESISTANCE TO P. S. PHASEOLICOLA 1); carbohydrate kinase
At5g09800	U-box domain-containing protein
At1g12110	NRT1.1,NRT1.1 (nitrate transporter 1.1); transporter
At1g21910	AP2 domain-containing transcription factor family protein
At5g20885	zinc finger (C3HC4-type RING finger) family protein
At5g22570	WRKY38,WRKY38 (WRKY DNA-binding protein 38); transcription factor
At4g24020	RWP-RK domain-containing protein
At3g13730	CYP90D1,CYP90D1 (CYTOCHROME P450, FAMILY 90, SUBFAMILY D, POLYPEPTIDE 1); oxidoreductase, acting on paired donors, with incorporation or reduction of molecular oxygen, NADH or NADPH as one donor, and incorporation of one atom of oxygen / oxygen binding
At4g30460	glycine-rich protein
At5g35630	GS2,GS2 (GLUTAMINE SYNTHETASE 2),GS2 (GLUTAMINE SYNTHETASE 2);

	glutamate-ammonia ligase
At3g55980	zinc finger (CCCH-type) family protein
At4g26390	pyruvate kinase, putative
At5g39590	unknown protein
At4g34760	auxin-responsive family protein
At5g48070	ATXTH20,ATXTH20 (XYLOGLUCAN ENDOTRANSGLUCOSYLASE/HYDROLASE 20); hydrolase, acting on glycosyl bonds
At5g14760	AO,AO (L-ASPARTATE OXIDASE); L-aspartate oxidase
At4g37240	unknown protein
At5g19890	peroxidase, putative
At1g23760	JP630,JP630; polygalacturonase
At4g04840	methionine sulfoxide reductase domain-containing protein / SeIR domain-containing protein
At1g30510	ATRFNR2,ATRFNR2 (ROOT FNR 2); oxidoreductase
At1g69440	AGO7,AGO7 (ARGONAUTE7)
At1g67340	zinc finger (MYND type) family protein / F-box family protein
At4g37180	myb family transcription factor
At3g47980	integral membrane HPP family protein
At5g15830	ATBZIP3,bZIP transcription factor family protein
At4g37540	LBD39,LOB domain protein 39 / lateral organ boundaries domain protein 39 (LBD39)
At3g15760	unknown protein
At3g07310	unknown protein
At5g58700	phosphoinositide phospholipase C,phosphoinositide-specific phospholipase C family protein
At1g68880	ATBZIP,bZIP transcription factor family protein
At5g01740	unknown protein
At5g59090	ATSBT4.12,subtilase,subtilase family protein
At5g54170	unknown protein
At2g18160	GBF5,ATBZIP2,GBF5 (G-box binding factor 5); DNA binding / transcription factor
At4g32950	protein phosphatase 2C, putative / PP2C, putative
At5g24655	unknown protein
At1g25550	myb family transcription factor
At1g49000	unknown protein
At5g57655	xylose isomerase family protein
At5g19120	pepsin A
AtCg00780	RPL14,unknown protein
At1g76340	integral membrane family protein
At3g05950	germin-like protein, putative
At5g24160	squalene monooxygenase 1,2 / squalene epoxidase 1,2 (SQP1,2)
At5g67390	unknown protein
At4g09620	unknown protein
At2g30040	MAPKKK14,MAPKKK14 (Mitogen-activated protein kinase kinase kinase 14); kinase
At5g48480	unknown protein
At3g04980	DNAJ heat shock N-terminal domain-containing protein
AtCg00660	RPL20,unknown protein
At3g49780	ATPSK4,ATPSK4 (PHYTOSULFOKINE 4 PRECURSOR); growth factor
At1g43710	EMB1075,EMB1075 (EMBRYO DEFECTIVE 1075); carboxy-lyase
At1g78100	F-box family protein

At1g70410	carbonic anhydrase, putative / carbonate dehydratase, putative
At3g50900	unknown protein
At4g37550	formamidase,formamidase, putative / formamide amidohydrolase, putative
At3g18710	U-box domain-containing protein
At2g17820	ATHK1,ATHK1 (HISTIDINE KINASE 1)
At3g06590	transcription factor,unknown protein
At1g29180	DC1 domain-containing protein
At5g50200	WR3,WR3 (WOUND-RESPONSIVE 3),WR3 (WOUND-RESPONSIVE 3); nitrate transporter
At1g72240	unknown protein
At3g17510	CIPK1,CIPK1 (CBL-INTERACTING PROTEIN KINASE 1); kinase
At1g20160	ATSBT5.2,subtilase,subtilase family protein
At1g67910	unknown protein
At4g34000	DPBF5,ABF3,ABF3/DPBF5 (ABSCISIC ACID RESPONSIVE ELEMENTS-BINDING FACTOR 3); DNA binding / protein binding / transcription factor/ transcriptional activator
At1g49230	zinc finger (C3HC4-type RING finger) family protein
At4g25835	AAA-type ATPase family protein
At5g52210	ATGB1,ATGB1 (Arabidopsis thaliana GTP-binding protein 1),ATGB1 (Arabidopsis thaliana GTP-binding protein 1); GTP binding
At3g53150	UGT73D1,UGT73D1 (UDP-glucosyl transferase 73D1); UDP-glycosyltransferase
At2g19460	unknown protein
At5g65210	TGA1,DNA binding / calmodulin binding / transcription factor,bZIP family transcription factor (TGA1)
At5g12860	DIT1,DIT1 (DICARBOXYLATE TRANSPORTER 1); oxoglutarate:malate antiporter,unknown protein
At4g02920	unknown protein
At4g31910	transferase family protein
At4g20460	NAD-dependent epimerase/dehydratase family protein
At3g27420	unknown protein
At5g67420	LBD37,LOB domain protein 37 / lateral organ boundaries domain protein 37 (LBD37)
At1g68670	myb family transcription factor
At1g78090	ATTPPB,ATTPPB (TREHALOSE-6-PHOSPHATE PHOSPHATASE)
At1g05750	PDE247,PDE247 (PIGMENT DEFECTIVE 247); binding
At5g13420	transaldolase, putative
At4g34710	ADC2,ADC2 (ARGININE DECARBOXYLASE 2)
At3g53620	ATPPA4,inorganic pyrophosphatase, putative (soluble) / pyrophosphate phospho-hydrolase, putative / PPase, putative
At3g49940	LBD38,LOB domain protein 38 / lateral organ boundaries domain protein 38 (LBD38)
At1g78050	phosphoglycerate/bisphosphoglycerate mutase family protein
At5g42830	transferase family protein

>Genes significantly regulated at 20min	
At5g62720	integral membrane HPP family protein
At1g69040	ACR4,ACR4 (ACT REPEAT 4); amino acid binding
At3g61590	HWS,HS,F-box family protein
At3g25790	myb family transcription factor

At2g40750	WRKY54,WRKY54 (WRKY DNA-binding protein 54); transcription factor
At3g58760	ankyrin protein kinase, putative
At3g60750	transketolase, putative
At2g28890	PLL4,PLL4 (POLTERGEIST LIKE 4); protein phosphatase type 2C
At2g47440	DNAJ heat shock N-terminal domain-containing protein
At3g07350	unknown protein
At1g21050	unknown protein
At4g36040	DNAJ heat shock N-terminal domain-containing protein (J11)
At4g39780	AP2 domain-containing transcription factor, putative
At1g77760	NIA1,NIA1 (NITRATE REDUCTASE 1)
At3g19030	unknown protein
At2g28690	unknown protein
At3g16150	L-asparaginase, putative / L-asparagine amidohydrolase, putative
At4g27900	unknown protein
At3g04060	ANAC046,ANAC046 (Arabidopsis NAC domain containing protein 46); transcription factor
At1g63940	monodehydroascorbate reductase, putative
At1g80380	phosphoribulokinase/uridine kinase-related
At5g41670	6-phosphogluconate dehydrogenase family protein
At2g22500	mitochondrial substrate carrier family protein
At2g27830	unknown protein
At2g17150	RWP-RK domain-containing protein
At5g28610	unknown protein
At4g31730	GDU1,GDU1 (GLUTAMINE DUMPER 1)
At1g12740	CYP87A2,CYP87A2 (cytochrome P450, family 87, subfamily A, polypeptide 2); oxygen binding
At1g64190	6-phosphogluconate dehydrogenase family protein
At3g04530	PPCK2,PPCK2 (PHOSPHOENOLPYRUVATE CARBOXYLASE KINASE 2); kinase
At3g11580	DNA-binding protein, putative
At1g68360	zinc finger protein-related
At1g71980	protease-associated zinc finger (C3HC4-type RING finger) family protein
At1g32920	unknown protein
At2g24550	unknown protein
At5g45340	CYP707A3,CYP707A3 (cytochrome P450, family 707, subfamily A, polypeptide 3); oxygen binding
At5g53460	GLT1,GLT1 (NADH-dependent glutamate synthase 1 gene)
At5g10210	unknown protein
At1g15550	GA4,GA4 (GA REQUIRING 4); gibberellin 3-beta-dioxygenase
At1g08100	ATNRT2.2,ATNRT2.2 (Arabidopsis thaliana high-affinity nitrate transporter 2.2); nitrate transporter
At1g70780	unknown protein
At5g25810	TNY,TNY (TINY); DNA binding / transcription factor
At1g62180	APR2,APR2 (5'ADENYLYLPHOSPHOSULFATE REDUCTASE 2)
At4g23980	ARF9,ARF9 (AUXIN RESPONSE FACTOR 9),ARF9 (AUXIN RESPONSE FACTOR 9); transcription factor
At5g65140	trehalose-6-phosphate phosphatase, putative,unknown protein
At4g18340	glycosyl hydrolase family 17 protein
At3g52360	unknown protein
At1g77400	unknown protein
At5g52170	homeobox-leucine zipper family protein / lipid-binding START domain-containing

	protein
At3g28050	nodulin MtN21 family protein
At5g08010	unknown protein
At4g03510	RMA1,RMA1 (Ring finger protein with Membrane Anchor 1); protein binding / ubiquitin-protein ligase/ zinc ion binding
At5g60770	ATNRT2.4,ATNRT2.4 (Arabidopsis thaliana high affinity nitrate transporter 2.4); nitrate transporter
At1g64140	unknown protein
At5g20540	BRX-LIKE4,ATBRXL4,ATBRXL4/BRX-LIKE4 (BREVIS RADIX-LIKE 4)
At1g49160	WNK7,WNK7 (Arabidopsis WNK kinase 7); kinase
At1g08090	ATNRT2:1,ATNRT2:1 (Arabidopsis thaliana high affinity nitrate transporter 2.1); nitrate transporter
At1g16420	ATMC8,latex-abundant protein, putative (AMC8) / caspase family protein
At5g47060	senescence-associated protein-related
At1g19050	ARR7,ARR7 (RESPONSE REGULATOR 7); transcription regulator/ two-component response regulator
At1g69760	unknown protein
At4g24620	PGI1,PGI1 (CHLOROPLASTIC PHOSPHOGLUCOSE ISOMERASE)
At5g10030	TGA4,TGA4 (TGACG MOTIF-BINDING FACTOR 4); DNA binding / calmodulin binding / transcription factor
At1g22170	phosphoglycerate/bisphosphoglycerate mutase family protein
At3g62930	glutaredoxin family protein
At5g04250	OTU-like cysteine protease family protein,unknown protein
At4g02380	SAG21,SAG21 (SENESCENCE-ASSOCIATED GENE 21)
At2g39370	unknown protein
At1g17060	CYP72C1,CYP72C1 (cytochrome P450, family 72, subfamily C, polypeptide 1); oxygen binding
At4g18510	CLE2,CLE2 (CLAVATA3/ESR-RELATED); receptor binding
At2g27510	ATFD3,ATFD3 (FERREDOXIN 3); electron carrier
At5g22890	zinc finger (C2H2 type) family protein
At4g05390	ATRFNR1,ATRFNR1 (ROOT FNR 1); oxidoreductase
At5g19260	unknown protein
At5g54130	
At4g00416	MBD3,MBD3 (methyl-CpG-binding domain 3); DNA binding
At4g37610	BT5,BT5 (BTB and TAZ domain protein 5); protein binding / transcription regulator
At1g72200	zinc finger (C3HC4-type RING finger) family protein
At2g28250	kinase,protein kinase family protein
At4g17550	transporter-related
At5g24890	unknown protein
At1g74660	MIF1,MIF1 (MINI ZINC FINGER 1); DNA binding / transcription factor
At2g38510	MATE efflux protein-related
At4g36010	pathogenesis-related thaumatin family protein
At4g40070	protein binding / zinc ion binding
At3g27210	unknown protein
At5g01340	mitochondrial substrate carrier family protein
At5g40470	unknown protein
At1g12820	AFB3,AFB3 (AUXIN SIGNALING F-BOX 3); auxin binding / ubiquitin-protein ligase
At1g66140	ZFP4,ZFP4 (ZINC FINGER PROTEIN 4); nucleic acid binding / transcription factor/ zinc ion binding
At3g52950	CBS domain-containing protein / octicosapeptide/Phox/Bemp1 (PB1) domain-

	containing protein
At2g26770	plectin-related
At5g35870	unknown protein
At1g13300	myb family transcription factor
At3g23030	IAA2,IAA2 (indoleacetic acid-induced protein 2); transcription factor
At3g30180	CYP85A2,BR6OX2,BR6OX2/CYP85A2 (BRASSINOSTEROID-6-OXIDASE 2); monooxygenase/ oxygen binding
At5g15330	SPX (SYG1/Pho81/XPR1) domain-containing protein
At5g40850	UPM1,UPM1 (UROPHORPHYRIN METHYLASE 1); uroporphyrin-III C-methyltransferase
At1g74940	senescence-associated protein-related
At3g29760	NLI interacting factor (NIF) family protein
At5g13110	G6PD2,G6PD2 (GLUCOSE-6-PHOSPHATE DEHYDROGENASE 2); glucose-6-phosphate 1-dehydrogenase
At5g19010	MPK16,MPK16 (mitogen-activated protein kinase 16); MAP kinase
At1g24280	G6PD3,G6PD3 (GLUCOSE-6-PHOSPHATE DEHYDROGENASE 3); glucose-6-phosphate 1-dehydrogenase
At5g03380	heavy-metal-associated domain-containing protein,metal ion binding
At5g58900	myb family transcription factor
At4g18250	receptor serine/threonine kinase, putative
At4g35260	IDH1,IDH1 (ISOCITRATE DEHYDROGENASE 1); isocitrate dehydrogenase (NAD+)
At5g04950	nicotianamine synthase, putative
At1g16170	unknown protein
At3g62630	unknown protein
At4g38340	RWP-RK domain-containing protein
At3g60490	AP2 domain-containing transcription factor TINY, putative
At2g48080	oxidoreductase, 2OG-Fe(II) oxygenase family protein
At5g04840	bZIP protein
At1g50110	branched-chain amino acid aminotransferase 6 / branched-chain amino acid transaminase 6 (BCAT6)
At3g46130	MYB111,MYB111 (myb domain protein 111),MYB111 (myb domain protein 111); DNA binding,MYB111 (myb domain protein 111); DNA binding / transcription factor
At2g15620	NIR1,NIR1 (NITRITE REDUCTASE); ferredoxin-nitrate reductase
At3g07340	basic helix-loop-helix (bHLH) family protein
At5g59730	ATEXO70H7,ATEXO70H7 (EXOCYST SUBUNIT EXO70 FAMILY PROTEIN H7); protein binding
At3g18560	unknown protein
At2g17060	disease resistance protein (TIR-NBS-LRR class), putative
At4g27180	ATK2,ATK2 (ARABIDOPSIS THALIANA KINESIN 2); microtubule motor
At4g18880	AT-HSFA4A,AT-HSFA4A (Arabidopsis thaliana heat shock transcription factor A4A); DNA binding / transcription factor
At1g21000	unknown protein,zinc-binding family protein
At3g60690	auxin-responsive family protein
At4g16447	unknown protein
At5g62900	unknown protein
At4g24670	alliinase family protein
At5g09800	U-box domain-containing protein
At1g12110	NRT1.1,NRT1.1 (nitrate transporter 1.1); transporter
At5g20885	zinc finger (C3HC4-type RING finger) family protein

At2g24790	COL3,COL3 (CONSTANS-LIKE 3); protein binding / transcription factor/ zinc ion binding,COL3 (CONSTANS-LIKE 3); zinc ion binding
At1g29160	Dof-type zinc finger domain-containing protein
At4g24020	RWP-RK domain-containing protein
At5g51050	mitochondrial substrate carrier family protein
At3g25780	AOC3,AOC3 (ALLENE OXIDE CYCLASE 3)
At3g13730	CYP90D1,CYP90D1 (CYTOCHROME P450, FAMILY 90, SUBFAMILY D, POLYPEPTIDE 1); oxidoreductase, acting on paired donors, with incorporation or reduction of molecular oxygen, NADH or NADPH as one donor, and incorporation of one atom of oxygen / oxygen binding
At5g47100	CBL9,CBL9 (Calcineurin B-like protein 9); calcium ion binding
At3g28510	AAA-type ATPase family protein
At5g35630	GS2,GS2 (GLUTAMINE SYNTHETASE 2),GS2 (GLUTAMINE SYNTHETASE 2); glutamate-ammonia ligase
At3g55980	zinc finger (CCCH-type) family protein
At4g26130	unknown protein
At3g14940	ATPPC3,ATPPC3 (PHOSPHOENOLPYRUVATE CARBOXYLASE 3); phosphoenolpyruvate carboxylase
At1g56230	unknown protein
At5g51830	pfkB-type carbohydrate kinase family protein
At4g34760	auxin-responsive family protein
At4g36540	BEE2,BEE2 (BR ENHANCED EXPRESSION 2); DNA binding / transcription factor
At5g14760	AO,AO (L-ASPARTATE OXIDASE); L-aspartate oxidase
At4g37240	unknown protein
At5g25280	serine-rich protein-related
At1g67920	unknown protein
At1g30510	ATRFNR2,ATRFNR2 (ROOT FNR 2); oxidoreductase
At1g67340	zinc finger (MYND type) family protein / F-box family protein
At4g37180	myb family transcription factor
At5g10820	integral membrane transporter family protein
At3g47980	integral membrane HPP family protein
At3g15760	unknown protein
At4g37540	LBD39,LOB domain protein 39 / lateral organ boundaries domain protein 39 (LBD39)
At5g15830	ATBZIP3,bZIP transcription factor family protein
At4g34750	auxin-responsive protein, putative / small auxin up RNA (SAUR_E)
At3g50910	unknown protein
At5g58700	phosphoinositide phospholipase C,phosphoinositide-specific phospholipase C family protein
At1g68880	ATBZIP,bZIP transcription factor family protein
At5g01740	unknown protein
At5g54170	unknown protein
At2g18160	GBF5,ATBZIP2,GBF5 (G-box binding factor 5); DNA binding / transcription factor
At1g61930	unknown protein
At4g32950	protein phosphatase 2C, putative / PP2C, putative
At5g24655	unknown protein
At1g25550	myb family transcription factor
At1g49000	unknown protein
At1g14260	zinc finger (C3HC4-type RING finger) family protein
At1g79110	protein binding / zinc ion binding

At1g13250	GATL3,GATL3 (Galacturonosyltransferase-like 3); polygalacturonate 4-alpha-galacturonosyltransferase/ transferase, transferring glycosyl groups / transferase, transferring hexosyl groups
At5g19120	pepsin A
At5g61420	MYB28,MYB28 (myb domain protein 28); DNA binding / transcription factor
At2g35270	DNA-binding protein-related
At2g31955	CNX2,CNX2 (COFACTOR OF NITRATE REDUCTASE AND XANTHINE DEHYDROGENASE 2); catalytic
At5g67390	unknown protein
At1g32170	XTR4,XTR4 (XYLOGLUCAN ENDOTRANSGLYCOSYLASE 4); hydrolase, acting on glycosyl bonds
At4g21350	PUB8,B80,B80; binding / ubiquitin-protein ligase
At4g09620	unknown protein
At1g13420	sulfotransferase family protein
At3g52900	unknown protein
At2g30040	MAPKKK14,MAPKKK14 (Mitogen-activated protein kinase kinase kinase 14); kinase
At2g31940	oxidoreductase/ transition metal ion binding
At1g43710	EMB1075,EMB1075 (EMBRYO DEFECTIVE 1075); carboxy-lyase
At1g75590	auxin-responsive family protein
At1g49860	ATGSTF14,ATGSTF14 (Arabidopsis thaliana Glutathione S-transferase (class phi) 14); glutathione transferase
At1g70410	carbonic anhydrase, putative / carbonate dehydratase, putative
At3g50900	unknown protein
At3g18710	U-box domain-containing protein
At2g17820	ATHK1,ATHK1 (HISTIDINE KINASE 1)
At3g06590	transcription factor,unknown protein
At1g22160	senescence-associated protein-related
At5g50200	WR3,WR3 (WOUND-RESPONSIVE 3),WR3 (WOUND-RESPONSIVE 3); nitrate transporter
At3g49760	ATBZIP5,bZIP transcription factor family protein
At2g42070	ATNUDT23,ATNUDT23 (Arabidopsis thaliana Nudix hydrolase homolog 23); hydrolase
At1g22640	MYB3,MYB3 (myb domain protein 3); DNA binding / transcription factor
At1g72240	unknown protein
At1g72830	HAP2C,HAP2C (Heme activator protein (yeast) homolog 2C); transcription factor
At3g17510	CIPK1,CIPK1 (CBL-INTERACTING PROTEIN KINASE 1); kinase
At1g49230	zinc finger (C3HC4-type RING finger) family protein
At4g34000	DPBF5,ABF3,ABF3/DPBF5 (ABSCISIC ACID RESPONSIVE ELEMENTS-BINDING FACTOR 3); DNA binding / protein binding / transcription factor/ transcriptional activator
At5g58690	phosphoinositide-specific phospholipase C family protein
At4g25835	AAA-type ATPase family protein
At3g57450	unknown protein
At3g28690	kinase,protein kinase, putative
At2g38170	CAX1,CAX1 (CATION EXCHANGER 1); calcium:hydrogen antiporter
At3g53150	UGT73D1,UGT73D1 (UDP-glucosyl transferase 73D1); UDP-glycosyltransferase
At5g64550	loricrin-related
At5g65210	TGA1,DNA binding / calmodulin binding / transcription factor,bZIP family transcription factor (TGA1)
At3g63110	ATIPT3,ATIPT3 (Arabidopsis thaliana isopentenyltransferase 3); transferase,

	transferring alkyl or aryl (other than methyl) groups
At5g12860	DIT1,DIT1 (DICARBOXYLATE TRANSPORTER 1); oxoglutarate:malate antiporter,unknown protein
At4g02920	unknown protein
At5g06570	unknown protein
At4g31910	transferase family protein
At5g67420	LBD37,LOB domain protein 37 / lateral organ boundaries domain protein 37 (LBD37)
At1g68670	myb family transcription factor
At1g78090	ATTPPB,ATTPPB (TREHALOSE-6-PHOSPHATE PHOSPHATASE)
At3g47520	MDH,MDH (malate dehydrogenase); malate dehydrogenase
At5g13420	transaldolase, putative
At3g26090	RGS1,RGS1 (REGULATOR OF G-PROTEIN SIGNALING 1); signal transducer
At4g16780	ATHB-2,ATHB-2 (Homeobox-leucine zipper protein HAT4); DNA binding / transcription factor
At3g49940	LBD38,LOB domain protein 38 / lateral organ boundaries domain protein 38 (LBD38)
At1g78050	phosphoglycerate/bisphosphoglycerate mutase family protein
At1g68500	unknown protein
At5g42830	transferase family protein
