# Supplementary figures and images for: Predictive network modeling of the high-resolution dynamic plant transcriptome in response to nitrate
Source: Genome Biol. 2010 Dec 23;11(12):R123. doi: 10.1186/gb-2010-11-12-r123 (PMC3046483; doi:10.1186/gb-2010-11-12-r123)

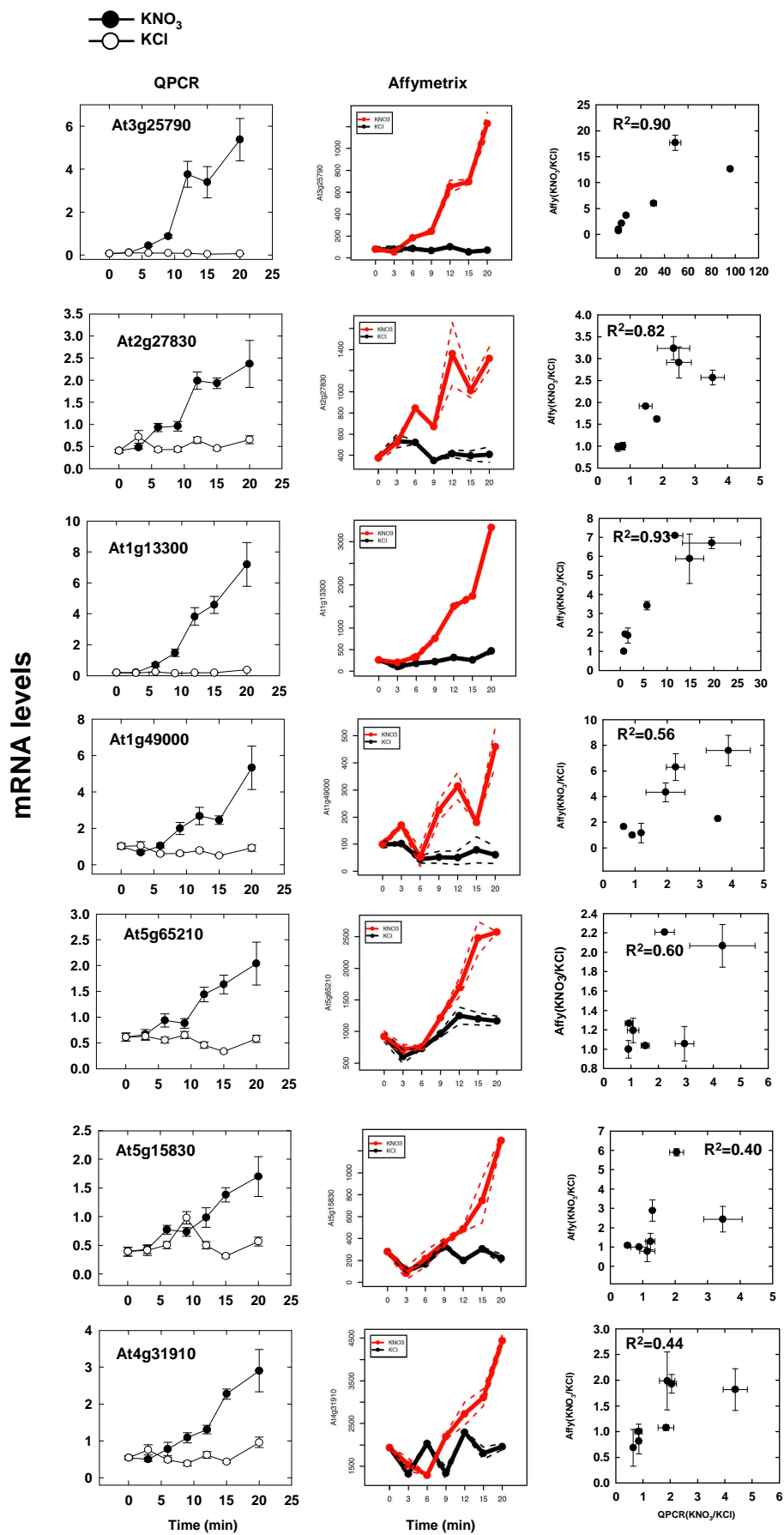

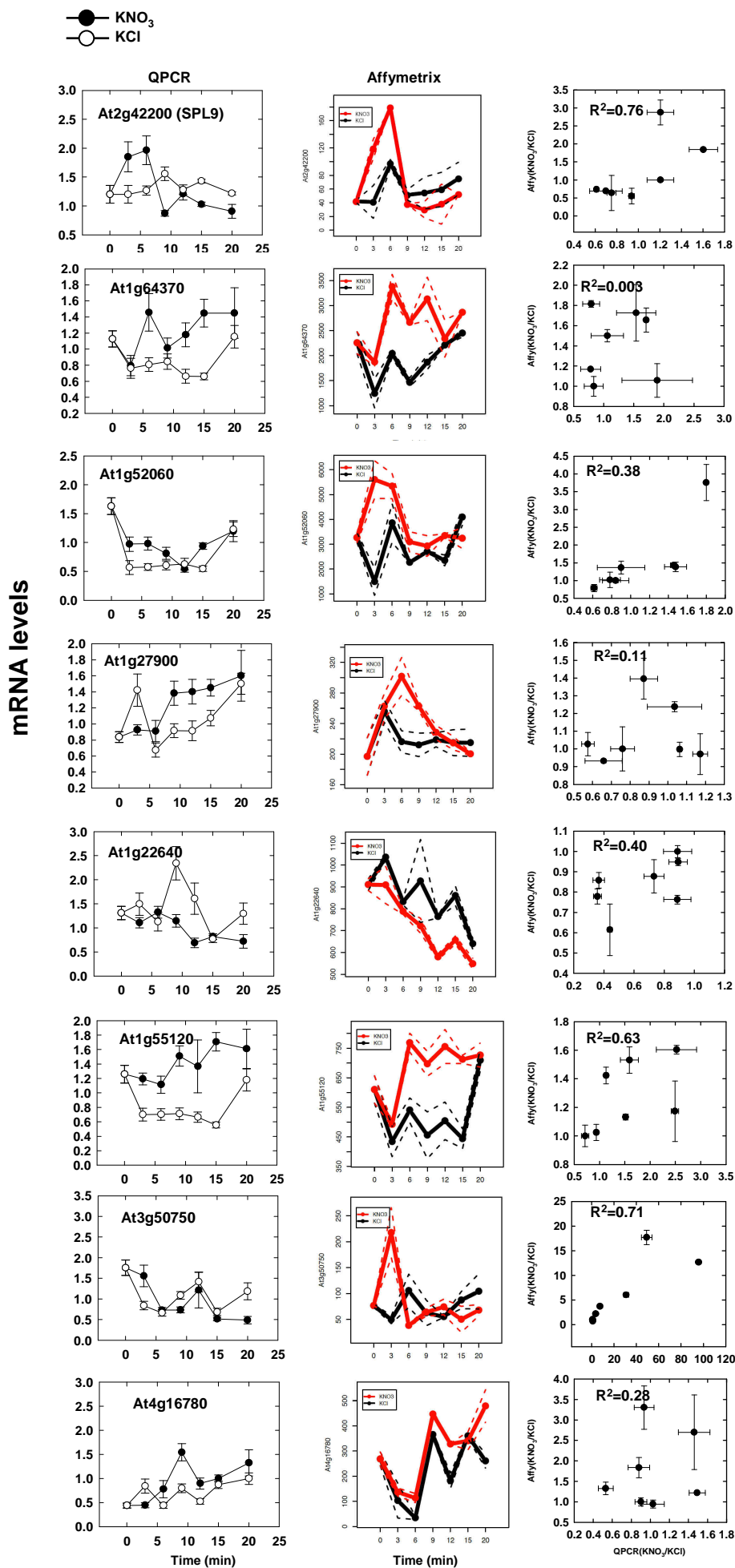

Supplement: Additional file 2 — High-resolution kinetics of response of genes to NO3- treatment. Levels of mRNA for nitrogen-responsive genes in Arabidopsis roots in response to NO3- treatment. Fourteen-day-old plants grown in the presence of ammonium succinate were treated with 1 mM KNO3 or KCL (as a mock treatment). Plants were collected at 0 minutes (before treatment) and 3, 6, 9, 12, 15, and 20 minutes after treatment. Transcripts were measured in RNA from roots using RT-QPCR and Affy ATH1 chips and normalized to two housekeeping genes (see Materials and methods). The data represent the mean ± standard error of three and two biological replicates for QPCR and Affymetrix measurements, respectively. Correlations between QPCR- and Affy-detected fold change results are provided in the third column. [file gb-2010-11-12-r123-S2.pdf]

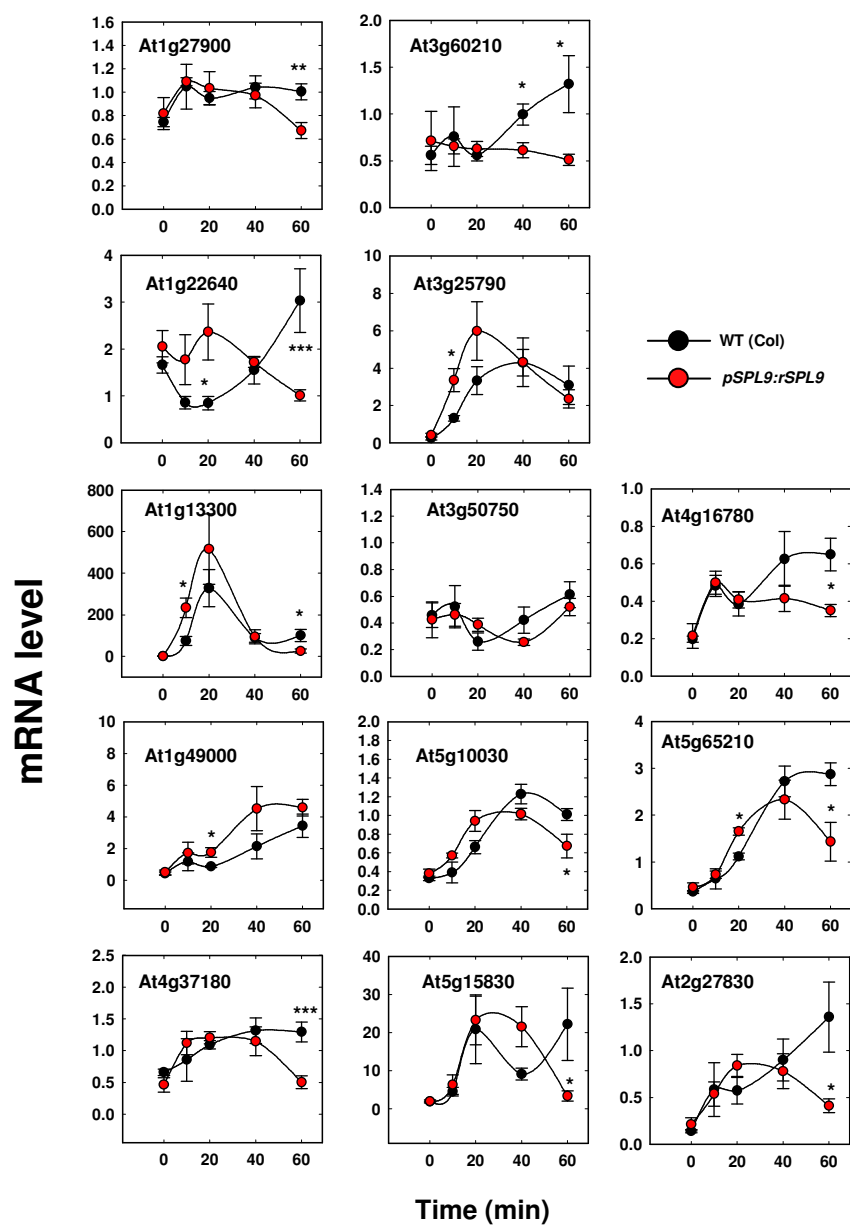

Supplement: Additional file 5 — SPL9 over-expression modifies NO3- kinetic responses of transcription factors. Transcription factor mRNA levels in roots of wild type and transgenic pSPL9:rSPL9 in response to NO3- treatment. Fourteen-day-old plants grown in ammonium succinate were treated with 1 mM KNO3 versus KCl. Roots were collected at 0 minutes (before treatment) and 10, 20, 40, and 60 minutes after the treatment. Sentinel transcripts were measured in roots using RT-QPCR and normalized to two housekeeping genes (see Materials and methods). The data represent the mean ± standard error of three biological replicates (three independent experiments). Differences between the two genotypes are statistically significant at *P < 0.05, **P < 0.01, and ***P < 0.001 (t-test). [file gb-2010-11-12-r123-S5.pdf]

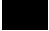 WT  
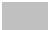 *pSPL9:rSPL9*

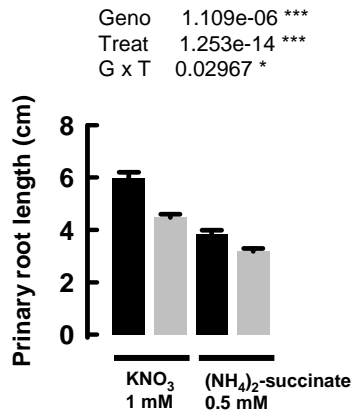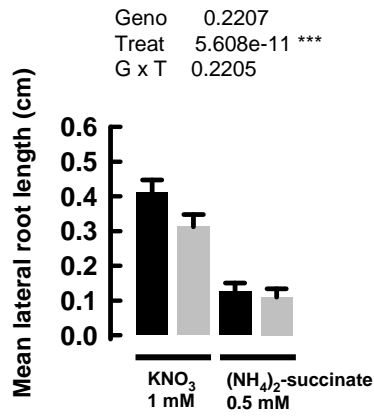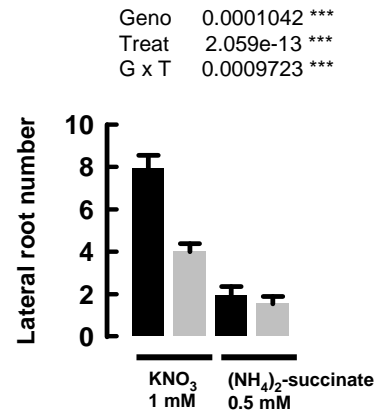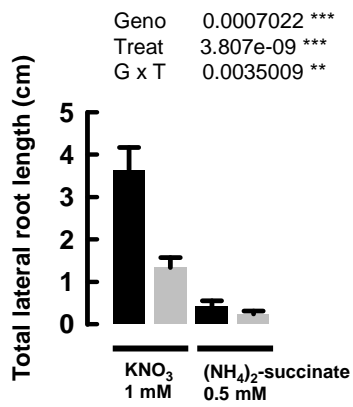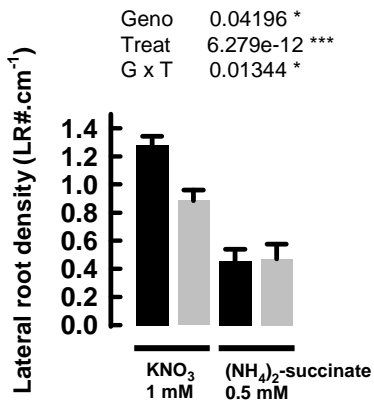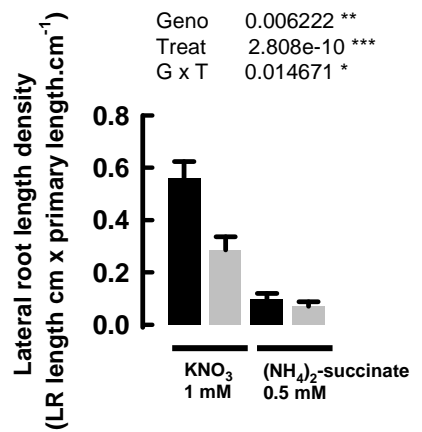

Supplement: Additional file 6 — NO3- presence enhances pSPL9:rSPL9 phenotypes. Wild type and pSPL9:rSPL9 have been plated on the same background media as used for kinetic experiments (see Materials and methods) and complemented with either 0.5 mM (NH4)2-succinate or 1 mM KNO3. Eight days after germination, different root traits were scored (n = 13 to 24 plants). ANOVA was used to evaluate the genotype effect (Geno: wild type versus pSPL9:rSPL9); the treatment effect (Treat: nitrate versus ammonium) and the interaction of these two factors (G × T). P-values are provided above each plot corresponding to different root parameters. ***P < 0.001; **P < 0.01; *P < 0.05. NO3- presence significantly enhances transgene effect for lateral root number, total lateral root length, lateral root density, and lateral root length density. The average length of lateral roots is not significantly controlled by the pSPL9:rSPL9 transgene. [file gb-2010-11-12-r123-S6.pdf]
