# Supplementary material for: Predictive network modeling of the high-resolution dynamic plant transcriptome in response to nitrate
Source: Genome Biol. 2010 Dec 23;11(12):R123. doi: 10.1186/gb-2010-11-12-r123 (PMC3046483; doi:10.1186/gb-2010-11-12-r123)
Supplement: Additional file 3 — Gene Ontology functions over-represented in NO3--regulated gene lists. [file gb-2010-11-12-r123-S3.pdf]

Term	Observed Frequency	Expected Frequency	p-value
**20min Induced**			
nitrate transport (5)	2.4%	<0.1%	7.80e-07
chemoperception and response (23)	11.0%	2.8%	3.43e-06
transcriptional control (38)	18.2%	7.6%	5.34e-05
mRNA synthesis (38)	18.2%	7.7%	7.42e-05
cellular sensing and response to external stimulus (28)	13.4%	4.7%	8.61e-05
ENERGY (15)	7.2%	1.5%	9.92e-05
INTERACTION WITH THE ENVIRONMENT (29)	13.9%	5.3%	0.00026
RNA synthesis (38)	18.2%	8.2%	0.00038
plant hormonal regulation (16)	7.7%	2.0%	0.00054
pentose-phosphate pathway oxidative branch (4)	1.9%	0.1%	0.00054
SYSTEMIC INTERACTION WITH THE ENVIRONMENT (17)	8.1%	2.4%	0.00191
plant / fungal specific systemic sensing and response (16)	7.7%	2.2%	0.00205
pentose-phosphate pathway non oxidative branch (3)	1.4%	<0.1%	0.00312
pentose-phosphate pathway (5)	2.4%	0.2%	0.00340
DNA binding (25)	12.0%	5.0%	0.00763
**15min Induced**			
chemoperception and response (19)	11.6%	2.8%	2.33e-05
nitrate transport (4)	2.4%	<0.1%	3.12e-05
pentose-phosphate pathway (6)	3.7%	0.2%	3.94e-05
pentose-phosphate pathway oxidative branch (4)	2.4%	0.1%	0.00019
cellular sensing and response to external stimulus (23)	14.0%	4.7%	0.00036
chloroplast (38)	23.2%	12.2%	0.00838
METABOLISM (49)	29.9%	17.6%	0.00989
**12min Induced**			
pentose-phosphate pathway oxidative branch (4)	3.3%	0.1%	4.43e-05
pentose-phosphate pathway (5)	4.1%	0.2%	0.00017
chemoperception and response (13)	10.6%	2.8%	0.00375
**9min Induced**			
pentose-phosphate pathway oxidative branch (4)	7.4%	0.1%	1.17e-06
pentose-phosphate pathway (4)	7.4%	0.2%	0.00013
pentose-phosphate pathway non oxidative branch (2)	3.7%	<0.1%	0.00692
**3min Induced**			
ribosomal proteins (7)	11.1%	0.6%	6.58e-06
ribosome biogenesis (7)	11.1%	1.4%	0.00181
**12min Depressed**			
oxygen and radical detoxification (4)	15.4%	0.9%	0.00281
CELL RESCUE DEFENSE AND VIRULENCE (7)	26.9%	4.9%	0.00580
