# Supplementary material for: Predictive network modeling of the high-resolution dynamic plant transcriptome in response to nitrate
Source: Genome Biol. 2010 Dec 23;11(12):R123. doi: 10.1186/gb-2010-11-12-r123 (PMC3046483; doi:10.1186/gb-2010-11-12-r123)
Supplement: Additional file 4 — Gene Ontology functions over-represented in NO3- clusters. [file gb-2010-11-12-r123-S4.pdf]

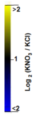

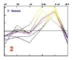

Term	Observed Frequency	Expected Frequency	p-value	Genes
**Cluster #2**				
ENERGY	3 out of 6 genes, 50.0%	415 out of 27532 genes, 1.5%	**0.00046**	At1g35290|At3g62410|At4g26390
chloroplast	4 out of 6 genes, 66.7%	3363 out of 27532 genes, 12.2%	**0.01901**	At1g35290|At1g18485|At3g62410|At4g18350
plastid	4 out of 6 genes, 66.7%	3386 out of 27532 genes, 12.3%	**0.01951**	At1g35290|At1g18485|At3g62410|At4g18350

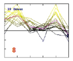

Term	Observed Frequency	Expected Frequency	p-value	Genes
**Cluster #8**				
metabolism of glycosinolates and derivatives	2 out of 15 genes, 13.3%	14 out of 27532 genes, 0.1%	**0.00045**	At5g23010|At5g23020
metabolism of leucine	2 out of 15 genes, 13.3%	23 out of 27532 genes, 0.1%	**0.00125**	At5g23010|At5g23020
metabolism of the pyruvate family (alanine, is	2 out of 15 genes, 13.3%	41 out of 27532 genes, 0.1%	**0.00403**	At5g23010|At5g23020
metabolism of secondary products derived fro	2 out of 15 genes, 13.3%	43 out of 27532 genes, 0.2%	**0.00444**	At5g23010|At5g23020

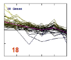

Term	Observed Frequency	Expected Frequency	p-value	Genes
**Cluster #18**				
unannotated	2 out of 38 genes, 5.3%	out of 27532 genes, 0.0%	**0**	AtCg00660|At3g26782
lipid binding	4 out of 38 genes, 10.5%	141 out of 27532 genes, 0.5%	**0.00199**	At1g35720|At4g27140|At5g65020|At2g27130
metabolism of cysteine	2 out of 38 genes, 5.3%	18 out of 27532 genes, 0.1%	**0.01315**	At3g61440|At3g01120
metabolism of the cysteine - aromatic group	3 out of 38 genes, 7.9%	133 out of 27532 genes, 0.5%	**0.03861**	At2g17630|At3g61440|At3g01120

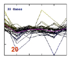

Term	Observed Frequency	Expected Frequency	p-value	Genes
**Cluster #20**				
ribosomal proteins	5 out of 39 genes, 12.8%	166 out of 27532 genes, 0.6%	**0.00017**	At2g27720|At5g20290|At3g05590|At1g77940|At3g11510
pyrimidine nucleotide/nucleoside/nucleobase	3 out of 39 genes, 7.7%	71 out of 27532 genes, 0.3%	**0.0066**	At1g75210|At4g09320|At4g11010
ribosome biogenesis	5 out of 39 genes, 12.8%	388 out of 27532 genes, 1.4%	**0.00988**	At2g27720|At5g20290|At3g05590|At1g77940|At3g11510

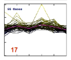

Term	Observed Frequency	Expected Frequency	p-value	Genes
**Cluster #17**				
C-compound and carbohydrate metabolism	14 out of 66 genes, 21.2%	1631 out of 27532 genes, 5.9%	**0.00162**	At4g04040|At3g06380|At1g22400|At1g05570|At4g15270|At3g57790|At1g80460|At1g52760|At3g09260|At1g62660|At1g23760|At3g47050|At5g36160|At3g10740
superoxide metabolism	2 out of 66 genes, 3.0%	14 out of 27532 genes, 0.1%	**0.03134**	At1g12520|At2g28190
METABOLISM	23 out of 66 genes, 34.8%	4848 out of 27532 genes, 17.6%	**0.03656**	At3g48990|At1g22400|At5g35980|At2g30860|At3g57790|At1g80460|At3g14050|At3g47050|At2g40890|At5g36160|At3g10740|At4g04040|At3g06380|At1g05570|At4g15270|At1g61360|At3g19010|At1g52760|At1g09570|At3g09260|At1g23760|At1g62660|At5g65110

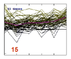

Term	Observed Frequency	Expected Frequency	p-value	Genes
**Cluster #15**				
ENERGY	7 out of 61 genes, 11.5%	415 out of 27532 genes, 1.5%	**0.00309**	At4g30190|At4g35260|At3g14940|At4g27180|At4g24620|At3g47520|At5g13420
chemoperception and response	9 out of 61 genes, 14.8%	770 out of 27532 genes, 2.8%	**0.004**	At1g74660|At1g19050|At3g14940|At2g38170|At5g50200|At3g60690|At5g62530|At5g61420|At1g69040
metabolism of glutamate	3 out of 61 genes, 4.9%	34 out of 27532 genes, 0.1%	**0.00501**	At5g53460|At5g35630|At5g62530
tricarboxylic-acid pathway (citrate cycle, Kreb	3 out of 61 genes, 4.9%	34 out of 27532 genes, 0.1%	**0.00501**	At4g35260|At3g14940|At3g47520
INTERACTION WITH THE ENVIRONMENT	12 out of 61 genes, 19.7%	1454 out of 27532 genes, 5.3%	**0.00598**	At5g10030|At1g74660|At1g19050|At3g14940|At2g38170|At5g50200|At3g60690|At5g62530|At5g17880|At5g61420|At4g27180|At1g69040
activity of intercellular mediators	3 out of 61 genes, 4.9%	38 out of 27532 genes, 0.1%	**0.00702**	At1g74660|At1g19050|At1g69040
cytokines (interleukines, colony stimulating fa	3 out of 61 genes, 4.9%	38 out of 27532 genes, 0.1%	**0.00702**	At1g74660|At1g19050|At1g69040
biosynthesis of glutamate	2 out of 61 genes, 3.3%	7 out of 27532 genes, 0.0%	**0.00855**	At5g53460|At5g62530
cytokinin response	3 out of 61 genes, 4.9%	41 out of 27532 genes, 0.1%	**0.00883**	At1g74660|At1g19050|At1g69040
cellular sensing and response to external stim	11 out of 61 genes, 18.0%	1297 out of 27532 genes, 4.7%	**0.00986**	At5g10030|At1g74660|At1g19050|At3g14940|At2g38170|At5g50200|At3g60690|At5g62530|At5g17880|At5g61420|At1g69040
nitrate transport	2 out of 61 genes, 3.3%	10 out of 27532 genes, 0.0%	**0.01825**	At5g50200|At5g60770
assimilation of ammonia, metabolism of the g	3 out of 61 genes, 4.9%	57 out of 27532 genes, 0.2%	**0.02363**	At5g53460|At5g35630|At5g62530

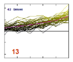

Term	Observed Frequency	Expected Frequency	p-value	Genes
**Cluster #13**				
pentose-phosphate pathway oxidative branch	3 out of 42 genes, 7.1%	15 out of 27532 genes, 0.1%	**6.82E-05**	At5g41670|At1g64190|At5g13110
pentose-phosphate pathway	3 out of 42 genes, 7.1%	46 out of 27532 genes, 0.2%	**0.0022**	At5g41670|At1g64190|At5g13110
pentose-phosphate pathway non oxidative bra	2 out of 42 genes, 4.8%	8 out of 27532 genes, 0.0%	**0.0029**	At5g41670|At1g64190

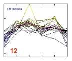

Term	Observed Frequency	Expected Frequency	p-value	Genes
**Cluster #12**				
peroxidase reaction	4 out of 18 genes, 22.2%	93 out of 27532 genes, 0.3%	**1.25E-05**	At5g39580|At5g64120|At1g14550|At1g14540
oxygen and radical detoxification	4 out of 18 genes, 22.2%	259 out of 27532 genes, 0.9%	**0.00073**	At5g39580|At5g64120|At1g14550|At1g14540
detoxification	4 out of 18 genes, 22.2%	264 out of 27532 genes, 1.0%	**0.00079**	At5g39580|At5g64120|At1g14550|At1g14540
response to biotic stimulus	3 out of 18 genes, 16.7%	221 out of 27532 genes, 0.8%	**0.01333**	At5g39580|At5g64120|At5g40990
plant defense response	2 out of 18 genes, 11.1%	82 out of 27532 genes, 0.3%	**0.04549**	At5g40990|At4g39950
