# Supplementary material for: Predictive network modeling of the high-resolution dynamic plant transcriptome in response to nitrate
Source: Genome Biol. 2010 Dec 23;11(12):R123. doi: 10.1186/gb-2010-11-12-r123 (PMC3046483; doi:10.1186/gb-2010-11-12-r123)
Supplement: Additional file 7 — QPCR primers used in this study. [file gb-2010-11-12-r123-S7.pdf]

gene Name	Forward Primer 5'->3'	Reverse Primer 5'->3'
At1g13300	atatagactgcatacaagaaggc	tagtccgattgtggtacccataaa
At1g22640	cttgctcggtaacaaatggtc	aagcttcctcttgatatgagtg
At1g27900	cggaatgtttgtgcagtagatatg	tactttgaaagagggagccg
At1g49000	cctttggcagagagagatattga	gcacttcgtttaatagccgtc
At1g52060	gatggtcctaattatgatggcg	ttgtcacaacttgtccgtct
At1g55120	gggttgggttaatgaatcgtc	cactgtagcaattcctttcctg
At1g64370	gcacaagaagaagaacaaggatg	tacttaatttagtcactcttagaagcgt
At2g27830	tcttcattactcgcagtttagtcttg	taaaccctccggtctctttcc
At3g25790	gtgagaccaccgggatttat	tcttgatctatcttccgaaatcttcct
At3g50750	gagctgacgctaggtcacaa	tacgatttaaccggaaccaatct
At3g60210	tccaatggcttcgagtttcattac	gaattccgagaagggtctgc
At4g16780	ctgcgacgaggatatctcac	gcatatctggtccatgaaact
At4g37180	aggagaagtctgatggacgta	agttggtaaagattatatcattcactatgc
At5g10030	tcctttgacggatcaacaactt	tatgttgcagtttctccatacct
At5g15830	tcttcgtcatcaacgagagga	ctgtgagagaagctcatctaagt
At5g65210	gaaccaacgtaggtttgagttat	ctctatttattcagagtagtcctctgt
CIPK23	gcacctatggggtttga	gtcccgtggtaaggtt
NIA1	gcctatgattcagtttgcg	agctctgataaacaacaactat
NIA2	cagtttgcggttcagc	tcgtgaatacaacgaacac
NIR	tgttcgtgtcacggag	aaacccaaaattatgggca
NRT1.1	gcacattggcattaggcttt	ctcaatccccacctcagcta
NRT2.1	aacaagggctaacgtggatg	ctgcttctcctgctcattcc
NRT3.1/NAR2.1	ggccatgaagttgcctatg	tcttggccttcctcttctca
SPL9	tggcagatcaatccaatgaatgtattt	caattcccttgtagctctctagttt
